# Cyclodextrin-Templated Porphyrin Nanorings**

**DOI:** 10.1002/anie.201402917

**Published:** 2014-06-10

**Authors:** Pengpeng Liu, Patrik Neuhaus, Dmitry V Kondratuk, T Silviu Balaban, Harry L Anderson

**Affiliations:** Department of Chemistry, University of Oxford, Chemistry Research Laboratory Oxford OX1 3TA (UK); Aix Marseille Université, ISM2, CNRS UMR 7313 Service 442, Ave Escadrille Normandie-Niemen, 13397 Marseille CEDEX 20 (France)

**Keywords:** cooperativity, cyclodextrins, flexibility, preorganization, templated synthesis

## Abstract

α- and β-Cyclodextrins have been used as scaffolds for the synthesis of six- and seven-legged templates by functionalizing every primary CH_2_OH with a 4-pyridyl moiety. Although these templates are flexible, they are very effective for directing the synthesis of macrocyclic porphyrin oligomers consisting of six or seven porphyrin units. The transfer of chirality from the cyclodextrin templates to their nanoring hosts is evident from NMR and circular dichroism spectroscopy. Surprisingly, the mean effective molarity for binding the flexible α-cyclodextrin-based template within the six-porphyrin nanoring (74 m) is almost as high as for the previously studied rigid hexadentate template (180 m). The discovery that flexible templates are effective in this system, and the availability of a template with a prime number of binding sites, open up many possibilities for the template-directed synthesis of larger macrocycles.

Floppy or rigid? The question of how much preorganization to build into a host–guest system can be a difficult dilemma.[1] The introduction of flexibility into a multivalent ligand generally reduces its affinity for a complementary receptor, but the energy cost of restricting conformational freedom can be surprisingly small.[2] Recently we have shown that rigid radial oligo-pyridine templates can be used to direct the synthesis of complementary zinc porphyrin nanorings,[3–5] and that the nanoring-template complexes can have huge stability constants (up to 10^36^ m^−1^).[3, 4, 6] Herein we report an investigation of flexible chiral templates based on cyclodextrin cores.

Cyclodextrins (CDs) are readily available cyclic oligomers of glucose with high molecular symmetries (*C*_6_, *C*_7_, and *C*_8_ for α-, β-, and γ-CD, respectively). We envisaged that the less-reactive secondary 2-OH and 3-OH groups of cyclodextrin molecules could be protected by methylation, while the primary 6-OH groups could be linked to 4-pyridyl moieties to form novel templates for nanoring synthesis.[7] Molecular mechanics calculations led to the design of T6* and T7* as suitable templates for 6- and 7-porphyrin nanorings, respectively (Figure 1). These two templates were synthesized from α- and β-CD using ester-coupling chemistry (see the Supporting Information for details).

**Figure 1:**
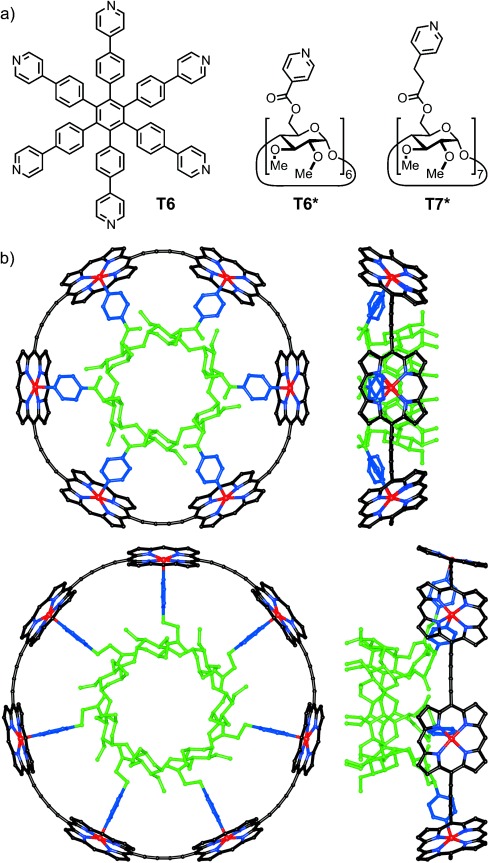
a) Rigid **T6** and flexible T6* and T7* cyclodextrin templates. b) Geometries of ***c***-P6⋅T6* and ***c***-P7⋅T7* from small-angle X-ray scattering and molecular mechanics calculations (MM+ force field, orthogonal views, *meso*-aryl groups and hydrogen atoms are omitted for clarity).

We first tested the ability of T6* to act as a template for the synthesis of ***c-*****P6** from three molecules of a linear porphyrin dimer, **P2**, under standard palladium-catalyzed oxidative coupling conditions. This reaction was remarkably effective and gave ***c***-P6⋅T6* in 59 % yield. Under the same conditions, **P2** couples in the presence of the rigid **T6** template to give ***c*****-P6⋅T6** in 62 % yield. Similarly, coupling the porphyrin monomer, **P1**, in the presence of T6* gave ***c***-P6⋅T6* in 22 % yield, together with ***c***-P12⋅(T6*)_2_ in 1.4 % yield (Scheme 1), whereas the analogous reaction of **T6** gives ***c*****-P6⋅T6** in 21 % yield and ***c*****-P12⋅(T6)_2_** in about 4 % yield. It is surprising that **T6** and T6* are equally good templates for these reactions. Coupling of **P1** in the presence of T7* gave the new 7-porphyrin nanoring complex ***c***-P7⋅T7* in 4.7 % yield. Addition of excess pyridine to ***c***-P6⋅T6* and ***c***-P7⋅T7* results in quantitative displacement of the templates to yield the free nanorings ***c*****-P6** and ***c*****-P7**. In both cases, re-addition of the template to the nanoring immediately regenerates the 1:1 complex.

**Scheme 1:**
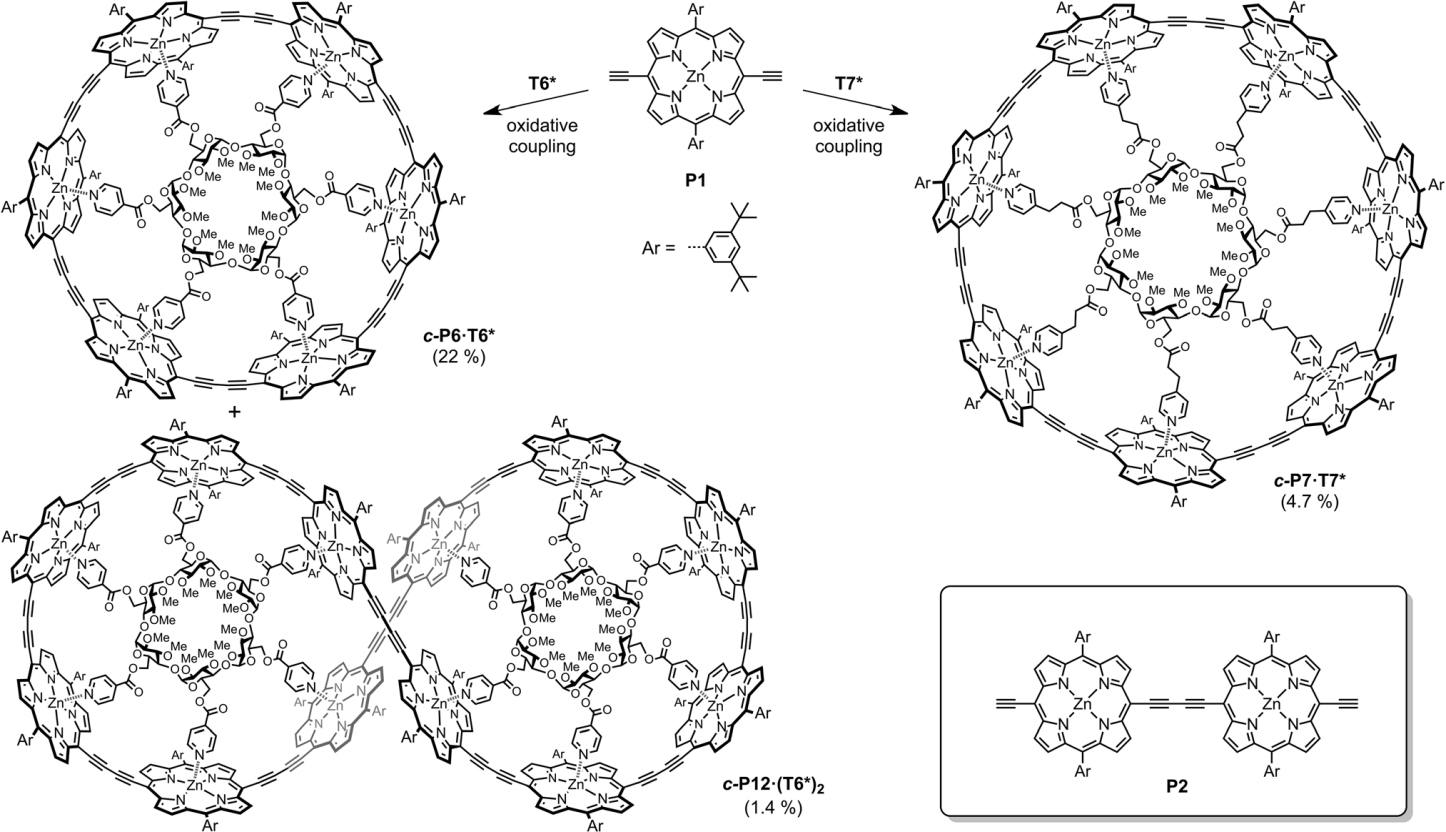
Syntheses of ***c***-P6⋅T6*, ***c***-P12⋅(T6*)_2_, and ***c***-P7⋅T7*. Inset: the structure of **P2**, which was also used as a precursor to ***c***-P6⋅T6*.

The ^1^H NMR spectra of ***c-***P6⋅T6* and ***c-***P7⋅T7* show that the cyclodextrins impose a chiral environment onto the porphyrin nanorings. Thus, whereas ***c-*****P6⋅T6** exhibits two β-pyrrole doublets, all eight β-pyrrole environments in each porphyrin unit of ***c-***P6⋅T6* and ***c-***P7⋅T7* are diastereotopic. Similarly, the three aryl resonances in ***c-*****P6⋅T6** split into six signals in ***c-***P6⋅T6* and ***c-***P7⋅T7* (Figure 2). The chirality of the conjugated π-systems is also evident from their circular dichroism spectra (Figure 3). The Cotton effect in ***c-***P12⋅(T6*)_2_ is about 20-fold stronger than in ***c-***P6⋅T6*, because in ***c-***P6⋅T6* the ***c-*****P6** unit is only slightly distorted away from its relaxed *D*_6*h*_ geometry, whereas the figure-of-eight ***c-*****P12** π-system has inherently chiral *D*_2_ symmetry, so that the chiral T6* template only needs to bias the equilibrium between the two enantiomeric conformations of the figure-of-eight.[8] The calculated geometries of ***c-***P6⋅T6* and ***c-***P7⋅T7* (Figure 1) were confirmed by small-angle X-ray scattering (SAXS) data from solutions in toluene.

**Figure 2:**
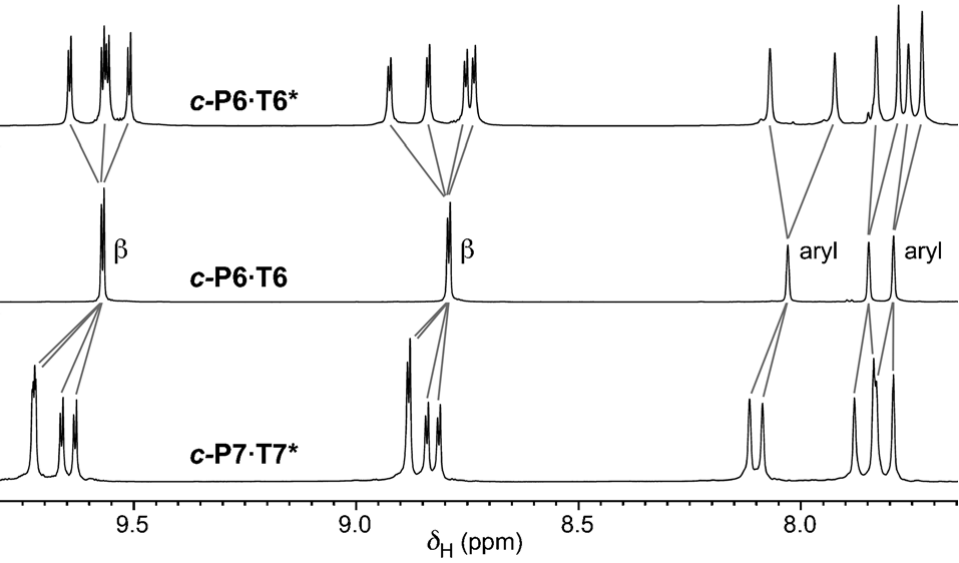
Partial ^1^H NMR spectra of ***c***-P6⋅T6*, ***c*****-P6⋅T6**, and ***c***-P7⋅T7* (CDCl_3_, 298 K, 700 MHz).

**Figure 3:**
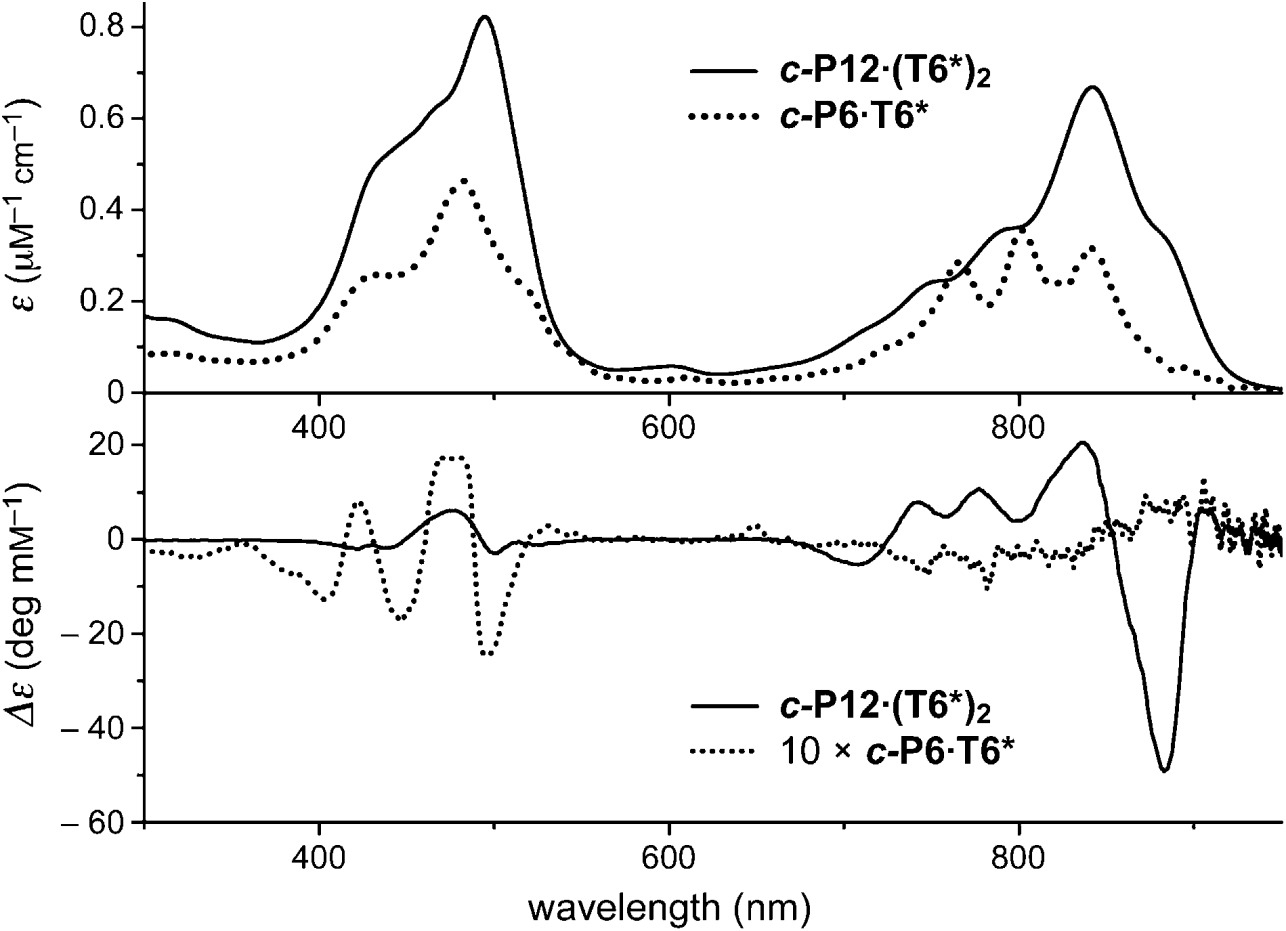
Extinction coefficient (*ε*) and molar circular dichroism (Δε) spectra of ***c***-P6⋅T6* and ***c***-P12⋅(T6*)_2_ in toluene (298 K; the Δε of ***c***-P6⋅T6* is multiplied by a factor of 10 for clarity).

The equilibrium constants, *K*_f_, for formation of the 1:1 complexes ***c-***P6⋅T6* and ***c-***P7⋅T7* provide a measure of how well the cyclodextrin templates fit their corresponding nanorings. These association constants are too high to measure by direct titration, but they can be measured by competition experiments, by displacing the templates with monodentate ligands, such as pyridine or quinuclidine. Simulation analysis of the binding isotherms for titration of ***c-*****P6⋅T6**, ***c-***P6⋅T6*, and ***c-***P7⋅T7* with quinuclidine (Figure 4) gave log *K*_f_=35.9±0.2, 29.0±0.2, and 32.0±0.8, respectively. Almost identical values were obtained by titration of ***c-***P6⋅T6* and ***c-***P7⋅T7* with pyridine (log *K*_f_=29.2±0.4 and 31.9±0.3, respectively) whereas pyridine does not bind strongly enough to dissociate ***c-*****P6⋅T6**. At first sight, the observation that ***c-*****P6⋅T6** is more stable than ***c-***P6⋅T6* by a factor of more than 10^6^ might be viewed as a consequence of the more flexible, less preorganized, conformational ensemble of the cyclodextrin-based template. However most of this difference in stability results from the electron-withdrawing effect of the *para*-CO_2_R substituent in T6*.[9]

**Figure 4:**
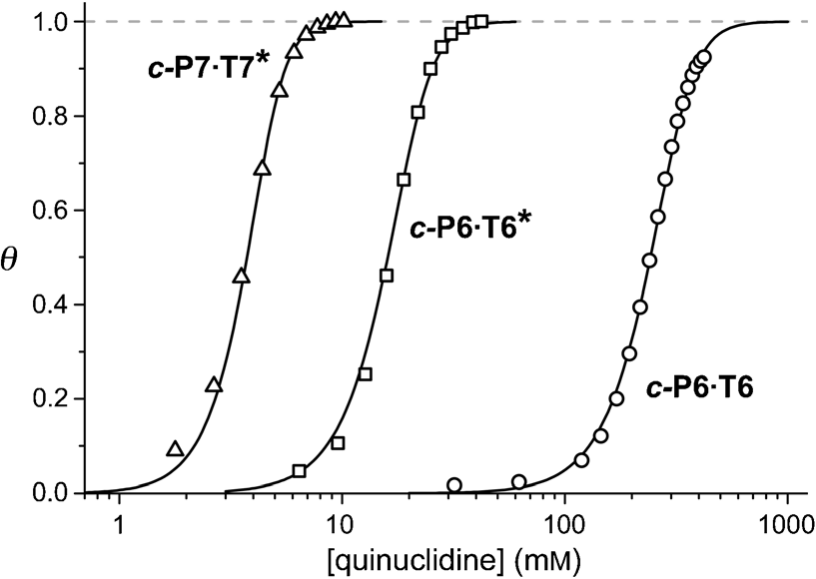
Displacement isotherms for titrating ***c***-P7⋅T7* (▵), ***c***-P6⋅T6* (□), and ***c*****-P6⋅T6** (○) with quinuclidine, all with an initial concentration of the nanoring complex of 0.34 μm in chloroform at 298 K. (*θ* is the mole fraction of the nanoring from which the template has been displaced.)

The complementarity of the templates for their nanorings can be compared without being distracted by variations in the Lewis basicity of the single-site interactions by calculating effective molarities using Equation (1), where *K*_f_ is the formation constant of the template-nanoring complex, *K*_σ_ is a statistical factor, *K*_1_ is the microscopic binding constant of the corresponding reference ligand (**L1**, **L2** or **L3**; Figure 5)with the nanoring (***c*****-P6** or ***c*****-P7**), *n* is the number of binding sites (6 or 7) and ${\overline {{\rm{EM}}} }$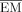 is the geometric mean of (*n*−1) individual effective molarities.[6, 10, 11]

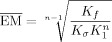
(1)

**Figure 5:**
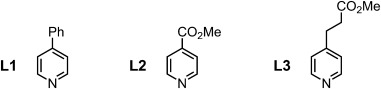
Structures of ligands **L1**, **L2**, and **L3** used as single-site reference compounds for estimating effective molarities for **T6**, T6*, and T7*, respectively.

This approach gives mean effective molarities of ${\overline {{\rm{EM}}} = }$
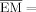
180±20 for ***c*****-P6⋅T6**, 74±20 for ***c***-P6⋅T6*, and 0.7±0.1 m for ***c***-P7⋅T7*. It is remarkable that ***c-*****P6⋅T6** and ***c-***P6⋅T6* have such similar effective molarities, and that the flexibility in T6* reduces its effective molarity by less than a factor of three. The similar ${\overline {{\rm{EM}}} }$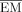 values of **T6** and T6* probably accounts for their remarkably similar abilities to direct the synthesis of ***c-*****P6**. The lower effective molarity measured in ***c***-P7⋅T7* is close to the value previously measured for an 8-porphyrin nanoring complex, ***c*****-P8⋅T8** (${\overline {{\rm{EM}}} = }$
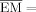
4 m),[3] and probably reflects the increase in flexibility with increasing molecular size, as well as the flexible –(CH_2_)_2_– links in T7*. The lower cooperativity in ***c***-P7⋅T7* must contribute to the lower yield in the template-directed synthesis of ***c*****-P7**.

In summary, we have synthesized flexible chiral cyclodextrin-based templates, and found that they are as effective as rigid templates for directing the synthesis of porphyrin nanorings. The discovery that strict preorganization is not necessary will make it easier to design templates for directing the formation of new macrocycles. The ability to hold a cyclodextrin at the center of a porphyrin nanoring opens up possibilities for creating many new photoactive supramolecular architectures by exploiting the recognition behavior of the cyclodextrin cavity.[12] Furthermore, the availability of a template, T7* with a prime number of coordination sites should make it possible to create very large macrocycles via Vernier templating.[5]

## Supporting Information

As a service to our authors and readers, this journal provides supporting information supplied by the authors. Such materials are peer reviewed and may be re-organized for online delivery, but are not copy-edited or typeset. Technical support issues arising from supporting information (other than missing files) should be addressed to the authors.

miscellaneous_information
