# Supplementary material for: Cyclodextrin-Templated Porphyrin Nanorings
Source: Angew Chem Int Ed Engl. 2014 Jun 10;53(30):7770–3. doi: 10.1002/anie.201402917 (PMC4298808; doi:10.1002/anie.201402917)
Supplement: Supplementary file 1 — miscellaneous_information [file anie0053-7770-SD1.pdf]

Angewandte Chemie
Eine Zeitschrift der Gesellschaft Deutscher Chemiker

Supporting Information
© Wiley-VCH 2014
69451 Weinheim, Germany

Cyclodextrin-Templated Porphyrin Nanorings**

*Pengpeng Liu, Patrik Neuhaus, Dmitry V. Kondratuk, T. Silviu Balaban, and Harry L. Anderson**

anie_201402917_sm_miscellaneous_information.pdf

Table of Contents

A. General Methods **S2**

B. Synthetic Procedures **S2**

C. Spectra Confirming Identity of New Compounds **S7**

D. Circular Dichroism Spectroscopy **S27**

E. Small-Angle X-ray Scattering Analysis of *c*-P6·T6* and *c*-P7·T7* **S27**

F. UV-Vis Titrations **S29**

*F1. Titrations of Monodentate Ligands with Porphyrin Monomer **P1'*** S29
*F2. Titrations of Monodentate Ligands with Nanorings **c-P6** and **c-P7*** S33
F3. Denaturation Titrations S39
F4. Calculation of Effective Molarities S43

G. References **S45**

A. General Methods

Dry toluene and THF were obtained by passing the solvents through columns of alumina, under nitrogen. Diisopropylamine (DIPA) was distilled from CaH_2 and kept over activated molecular sieves (3 Å, 8–12 mesh). Unless specified otherwise, all other solvents were used as commercially supplied.

Flash chromatography was carried out on silica gel 60 under positive pressure. Analytical thin-layer chromatography was carried out on aluminum-backed silica gel 60 F254 plates. Visualization was achieved using UV light, iodine dip or $KMnO_4$ dip, when necessary.

All UV-vis spectra were recorded in solution using a Perkin-Lambda 20 spectrometer (1 cm path length quartz cell). Unless stated otherwise, $^{1}H/^{13}C$ NMR spectra were recorded at 298 K using Bruker AV400 (400/100 MHz), Bruker AV500 (500/125 MHz) and Bruker AV700 (700/175 MHz) instruments. ^{1}H, ^{19}F and ^{13}C NMR spectra are reported in ppm; coupling constants are given in Hertz, to the nearest 0.1 Hz.

Electronic circular dichroism spectra were recorded on a JASCO 815 instrument from 950 to 250/280 nm, and were measured in a quartz cuvette from Hellma with 1 mm pathlength. The spectra of the samples were recorded in toluene solution after subtraction from the baseline and were an average of two or four scans.

ESI mass spectra were carried out either using Fisons Platform or Micromass LCT spectrometer. MALDI-TOF mass spectra were carried out using Waters MALDI Micro MX spectrometer.

B. Synthetic Procedures

Porphyrin monomer **P1**[S1], dimer **P2**[S1], hexamer ***c*-P6**[S2], ***c*-P6·T6**[S2], per-2,3-di-*O*-methyl-α-cyclodextrin[S3,S4] (precursor for **T6***), per-2,3-di-O-methyl-β-cyclodextrin[S3,S4] (precursor for **T7***) and isonicotinic acid pentafluorophenyl ester (precursor for **T6***)[S5] were synthesized using published procedures.

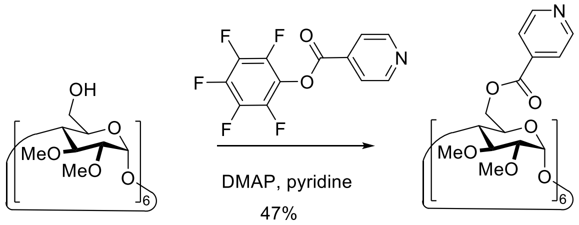

T6*

<u>Per-2,3-di-*O*-methyl-6-*O*-(*para*-pyridinyl)carboxyl-α-cyclodextrin(T6*).</u>

Per-2,3-di-*O*-methyl-α-cyclodextrin[S3,S4] (120 mg, 0.106 mmol), 4-dimethylaminopyridine (230 mg, 1.88 mmol) and isonicotinic acid pentafluorophenyl ester (750 mg, 2.59 mmol) were dried under vacuum for over 2 h, anhydrous pyridine (7.5 mL) was added. The mixture was stirred at 90 °C for 24 h, at which point MALDI-MS analysis indicated the completion of the reaction. The reaction mixture was cooled and the solvent was evaporated under vacuum. The residue was dissolved in chloroform (120 mL) and washed with water (120 mL × 2). The organic solution was evaporated to dryness. Column chromatography (chloroform/MeOH, 20/1, v/v) of the residue gave the product **T6*** as white powder (87 mg, 47%), $R_f = 0.05$. ^{1}H NMR (400 MHz, $CDCl_3$): δ = 8.76 (d, J = 5.8 Hz, 12H), 7.79 (d, J = 5.8 Hz, 12H), 4.98 (d, J = 3.3 Hz, 6H), 4.67 (d, J = 11.2 Hz, 6H), 4.48 (dd, J = 12.2, 3.7 Hz, 6H), 4.08 (d, J = 6.7 Hz, 6H), 3.68 (m, 18H), 3.65–3.58 (m, 6H), 3.52 (s, 18H), 3.18 (dd, J = 9.5, 3.2 Hz, 6H); ^{13}C NMR (100 MHz, $CDCl_3$): δ = 164.64, 150.99, 136.89, 122.96, 100.53, 82.69, 82.06, 81.53, 70.20, 64.41, 62.09, 58.75; MALDI-TOF MS+: *m/z* 1794.09 ($[M+Na]^+$, $C_{84}H_{102}N_6O_{36}Na$ requires: 1793.62).

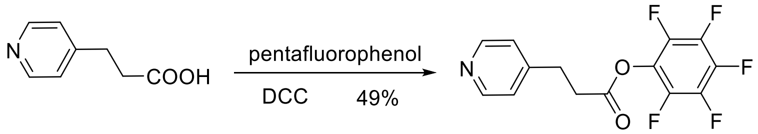

4-Pyridinyl propanoic acid pentafluorophenol ester. 4-Pyridinyl propanoic acid (2.81 g, 18.8 mmol), pentafluorophenol (3.66 g, 19.9 mmol) and *N*,*N'*-dicyclohexylcarbodiimide (DCC, 4.63 g, 22.4 mmol) were added into a 250 mL flask. The flask was evacuated and filled with N_2. THF (80 mL) was added under N_2 and the reaction mixture was stirred under N_2 protection at room temperature for 24 h. The solid generated was filtered off and the THF solvent in the filtrate was evaporated under vacuum. The resulting residue was crystallized from petroleum ether to give the product as white solid (2.9 g, 49%). ^{1}H NMR (400 MHz, $CDCl_3$) δ = 8.58 (d, *J* = 6.0 Hz, 2H), 7.24 (d, *J* = 6.0 Hz, 2H), 3.17–3.10 (m, 2H), 3.10–3.03 (m, 2H); ^{19}F NMR (377 MHz, $CDCl_3$) δ = –152.62 (d, *J* = 17.1 Hz, 2F), –157.49 (t, *J* = 21.7 Hz, 1F), –161.98 (dd, *J* = 21.7, 17.1 Hz, 2F); ESI-HDMS+: *m/z* 318.0534 ($[M+H]^+$, $C_{14}H_9F_5NO_2$ requires: 318.0548).

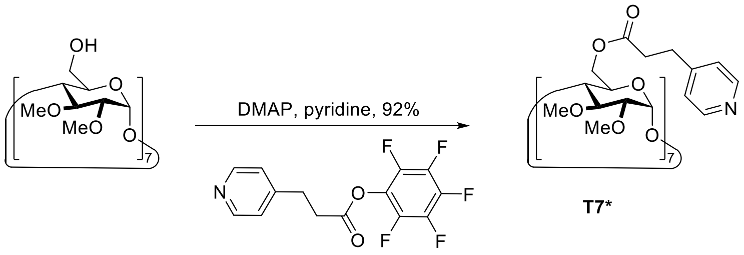

Per-2,3-di-*O*-methyl-6-*O*-(4-pyridinylethyl)carboxyl-β-cyclodextrin (T7*).

Per-2,3-di-O-methyl-β-cyclodextrin[S3,S4] (87.7 mg, 0.066 mmol), 4-pyridinyl propanoic acid pentafluorophenol ester (670 mg, 2.11 mmol), 4-dimethylamino pyridine (162 mg, 1.32 mmol) were dried under vacuum for over 2 h, then pyridine (7.0 mL) was added. The mixture was stirred at 90°C for 5 h. MALDI-MS analysis indicated the completion of the reaction. The reaction mixture was cooled and the solvent was evaporated under vacuum. The residue was dissolved in chloroform (100 mL) and washed with water (100 mL × 2). The organic solution was evaporated to dryness. Column chromatography (chloroform/MeOH, 20/1, gradually increasing the polarity to 20/1.6, v/v) washed off the excess pentafluorophenol ester. The column was then eluted with solvent of increasing polarity (chloroform/MeOH, 1/1, v/v) to give the product, along with impurities which do not dissolve in pure chloroform. The product was finally extracted from the residue using chloroform and evaporation of the solvent gave the product **T7*** as light yellowish solid (137 mg, 92.1%). ^{1}H NMR (400 MHz, $CDCl_3$) δ = 8.39 (d, *J* = 5.9 Hz, 14H), 7.05 (d, *J* = 5.7 Hz, 14H), 4.86 (d, *J* = 3.5 Hz, 7H), 4.36 (d, *J* = 11.6 Hz, 7H), 4.20 (dd, *J* = 12.1, 3.7 Hz, 7H), 3.78 (d, *J* = 7.4 Hz, 7H), 3.55 (s, 21H), 3.44 (s, 21H), 3.43–3.37 (m, 14H), 3.05 (dd, *J* = 9.0, 3.3 Hz, 7H), 2.88–2.78 (m, 14H), 2.71–2.49 (m, 14H); ^{13}C NMR (100 MHz, $CDCl_3$) δ = 171.79, 149.75, 149.33, 123.70 , 99.09, 81.54, 81.47, 80.38, 69.56, 63.22, 61.32, 58.83, 34.00, 29.81; MALDI-TOF MS+: *m/z* 2263.90 ($[M]^+$, $C_{112}H_{147}N_7O_{42}$ requires: 2263.97).

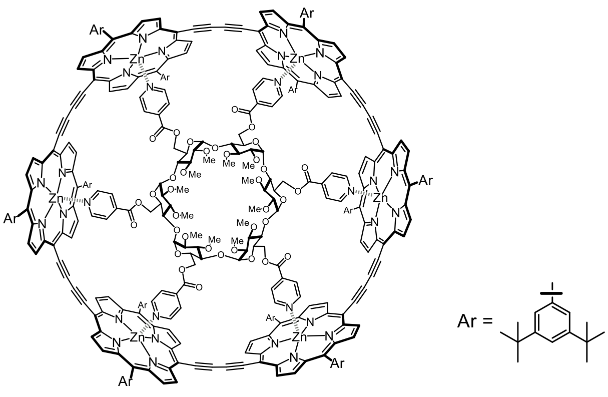

c*-P6·T6

Preparation of *c*-P6·T6*

a) **T6*** (5.5 mg, 3.13 μmol) and **P2** (20 mg, 12.5 μmol) were dissolved in chloroform (15 mL) and diisopropylamine (DIPA, 0.20 mL). The solution was sonicated for 2 h and cooled to room temperature. A catalyst solution was prepared by dissolving dichlorobis(triphenyl-phosphine)-palladium(II) (2.9 mg, 4.0 μmol), CuI (7.2 mg, 37 μmol) and 1,4-benzoquinone (5.4 mg, 50 μmol) in chloroform (15 mL) and DIPA (0.2 mL). The catalyst solution was added to the mixture and stirred under air at room temperature overnight. The reaction mixture was passed through a plug of alumina using chloroform as eluent. The crude mixture was separated on size exclusion column (Biobeads SX-1, 200–400 mesh) using toluene as eluent to give the product ***c*-P6·T6*** as reddish-brown solid (12 mg, 59%).

b) **T6*** (5.5 mg, 3.13 μmol) and **P1** (20 mg, 25 μmol) were dissolved in chloroform (15 mL) and diisopropylamine (DIPA, 0.20 mL). The solution was sonicated for 2 h and cooled to room temperature. A catalyst solution was prepared by dissolving dichlorobis(triphenyl-phosphine)-palladium(II) (2.9 mg, 4.0 μmol), CuI (7.2 mg, 37 μmol) and 1,4-benzoquinone (10.8 mg, 100 μmol) in chloroform (15 mL) and DIPA (0.2 mL). The catalyst solution was added to the mixture and the reaction was stirred under air at room temperature and UV-vis monitoring during the reaction process (0.5 h interval) indicated the reaction to completion at 6 h. The reaction mixture was passed through a plug of alumina using chloroform as eluent. The crude mixture was separated on size exclusion column (Biobeads SX-1, 200–400 mesh) using toluene as eluent. Two major bands were collected from the column: the first band was collected for the following ***c*-P12·(T6*)$_2$** synthesis; the second band was collected, dried and precipitated using chloroform/methanol to give the product ***c*-P6·T6*** as reddish-brown solid (4.5 mg, 22%).

^{1}H NMR (700 MHz, $CDCl_3$): δ = 9.64 (d, J = 4.2 Hz, 6H), 9.56 (d, J = 4.2 Hz, 6H), 9.55 (d, J = 4.2 Hz, 6H), 9.51 (d, J = 4.2 Hz, 6H), 8.92 (d, J = 4.2 Hz, 6H), 8.84 (d, J = 4.2 Hz, 6H), 8.75 (d, J = 4.2 Hz, 6H), 8.73 (d, J = 4.2 Hz, 6H), 8.07 (s, 6H), 7.92 (s, 6H), 7.83 (s, 6H), 7.78 (s, 6H), 7.76 (s, 6H), 7.73 (s, 6H), 5.45 (d, J = 7.3 Hz, 12H), 3.37 (d, J = 2.8 Hz, 6H), 3.15 (d, J = 10.0 Hz, 6H), 3.06 (s, 18H), 2.86 (s, 18H), 2.78 (d, J = 11.0 Hz, 6H), 2.62–2.59 (m, 12H), 2.37 (d, J = 7.3 Hz, 12H), 2.32 (d, J = 10.0 Hz, 6H), 2.17 (dd, J = 10.0 Hz, J = 2.8 Hz, 6H), 1.51 (s, 108H), 1.50 (s, 54H), 1.46 (s, 54H); MALDI-TOF MS+: *m/z* 4775 ([M-**T6***]$^+$, $C_{312}H_{300}N_{24}Zn_6$ requires: 4778), 6544 ([M]$^{+\bullet}$, $C_{396}H_{402}N_{30}Zn_6O_{36}$ requires: 6549); UV-vis ($CHCl_3$): λ_{max} (ε) 483 (5.0×10^5), 772 (3.2×10^5), 808 (4.1×10^5), 848 (3.5×10^5).

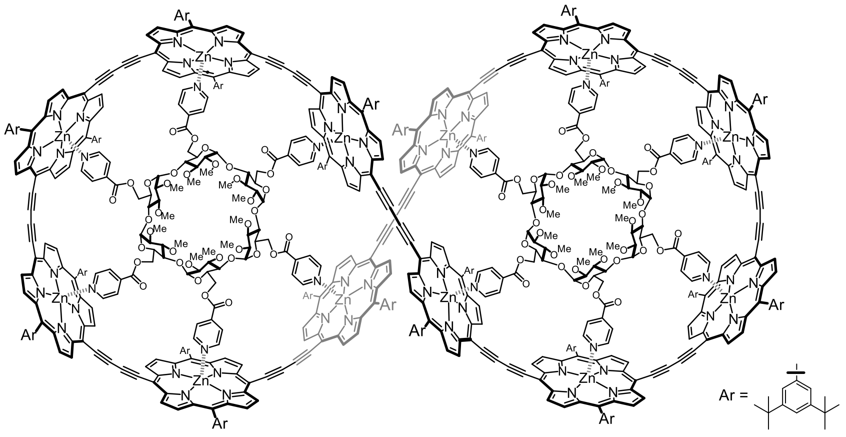

c-P12·(T6*)₂

Preparation of *c*-P12·(T6*)$_2$. The mixture collected in the procedure (b) in the ***c*-P6·T6*** synthesis was separated on size exclusion column (Biobeads SX-1, 200–400 mesh) using chloroform/pyridine (10/1, v/v) to remove the template. Recycling GPC (Shimadzu recycling GPC system equipped with LC-20 AD pump, SPD-20A UV detector and a set of JAIGEL 3H (20 x 600 mm) and JAIGEL 4H (20 x 600 mm) columns in toluene/1% pyridine as eluent, flow rate 3.5 mL/min) gave the template-free ***c*-P12** intermediate (0.40 mg, 2.0%). ***c*-P12** (0.40 mg, 0.042 μmol)and **T6*** (0.20 mg, 0.11 μmol) were dissolved in chloroform (2 mL) and plugged through a short basic alumina column. The solvent was evaporated and the residue was precipitated using chloroform/methanol to give the product ***c*-P12·(T6*)$_2$** as black solid (0.39 mg, 70% from ***c*-P12**, 1.4% from **P1**).

^{1}H NMR: see Figure S15; MALDI-TOF MS+: *m/z* 11318 ([M-**T6***]$^+$, $C_{708}H_{702}N_{54}Zn_{12}O_{36}$ requires: 11327), 9543 ([M-2**T6***]$^+$, $C_{624}H_{600}N_{48}Zn_{12}$ requires: 9555). UV-vis ($CHCl_3$): λ_{max} (ε) 496 (8.1×10^5), 763 (3.1×10^5), 800 (3.8×10^5), 838 (4.3×10^5), 874 (6.7×10^5).

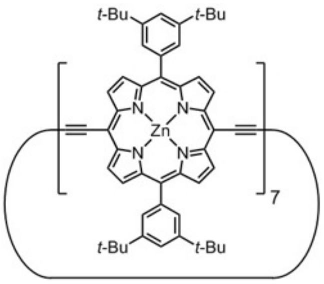

***c*-P7**

Preparation of *c*-P7. **T7***(11.4 mg, 5.0 μmol) and **P1** (40 mg, 50 μmol) were dissolved in chloroform (60 mL) and DIPA (1.0 mL). The solution was sonicated for 1 h. A catalyst mixture consisting of dichlorobis(triphenyl-phosphine)-palladium(II) (5.8 mg, 8.0 μmol), CuI (15 mg, 75 μmol) and 1,4-benzoquinone (22 mg, 0.20 mmol) was added. The reaction was stirred under air at room temperature overnight, then the mixture was passed through a short plug of alumina using chloroform/pyridine (8/1, v/v) as eluent. The crude mixture was separated on size exclusion column (Biobeads SX-1, 200–400 mesh) using chloroform/pyridine (8/1, v/v) to remove the template. Recycling GPC (Shimadzu recycling GPC system equipped with LC-20 AD pump, SPD-20A UV detector and a set of JAIGEL 3H (20 x 600 mm) and JAIGEL 4H (20 x 600 mm) columns in toluene/1% pyridine as eluent, flow rate 3.5 mL/min) gave the product ***c*-P7*** as dark brown solid (2.1 mg, 5.2%).

^{1}H NMR (400 MHz, $CDCl_3$): δ = 9.63 (d, *J* = 4.4 Hz, 28H), 8.78 (d, *J* = 4.4 Hz, 28H), 7.89 (d, *J* = 2.0 Hz, 28H), 7.70 (t, *J* = 2.0 Hz, 14H), 1.44 (s, 252H); MALDI-TOF MS+: *m/z* 5571, ([M]$^+$, $C_{364}H_{350}N_{28}Zn_7$ requires: 5575); UV-vis ($CHCl_3$): λ_{max} (ε) 458 (5.4×10^5), 494 (5.2×10^5), 772 (2.8×10^5).

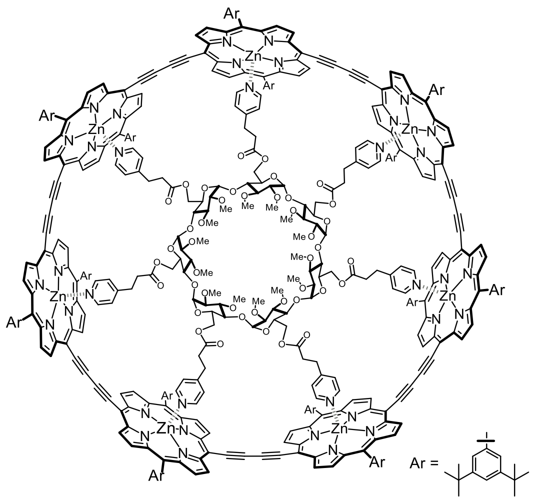

c*-P7·T7

Preparation of *c*-P7·T7*. **T7*** (5.0 mg, 2.2 μmol) and ***c*-P7** (3.5 mg, 0.63 μmol) were mixed in chloroform and the solution was passed through a short basic alumina column using chloroform as the eluent. The compound was precipitated by dissolving in chloroform and adding methanol to give the product ***c*-P7·T7*** as dark brown solid (4.4 mg, 90% from ***c*-P7**, 4.7% from **P1**).

^{1}H NMR (700 MHz, $CDCl_3$): δ = 9.73 (d, *J* = 1.8 Hz, 7H), 9.72 (d, *J* = 1.8 Hz, 7H), 9.66 (d, *J* = 4.2 Hz, 7H), 9.63 (d, *J* = 4.2 Hz, 7H), 8.88 (d, *J* = 4.2 Hz, 14H), 8.84 (d, *J* = 4.2 Hz, 7H), 8.81 (d, *J* = 4.2 Hz, 7H), 8.11 (s, 7H), 8.08 (s, 7H), 7.88 (s, 7H), 7.84 (s, 14H), 7.79 (s, 7H), 4.99 (d, *J* = 7.2 Hz, 14H), 4.04 (d, *J* = 3.0 Hz, 7H), 3.29 (d, *J* = 11.5 Hz, 7H), 3.21 (s, 28H), 3.05 (s, 21H), 2.91 (d, *J* = 7.2 Hz, 7H), 2.74–2.70 (m, 14H), 2.44–2.41 (m, 21H), 1.58–1.52 (m, 252H), 1.34–1.31 (m, 14H), 1.06 (t, *J* = 8.0 Hz, 14H); MALDI-TOF MS+: *m/z* 5569 ([M-**T7**]$^+$, $C_{364}H_{350}N_{28}Zn_7$ requires: 5575), 7829 ([M]$^{+\bullet}$, $C_{476}H_{497}N_{35}O_{42}Zn_7$ requires: 7837); UV-vis ($CHCl_3$): λ_{max} (ε) 464 (5.0×10^5), 509 (5.7×10^5), 767 (2.7×10^5), 807 (4.1×10^5), 853 (7.0 ×10^5).

C. Spectra Confirming Identity of New Compounds

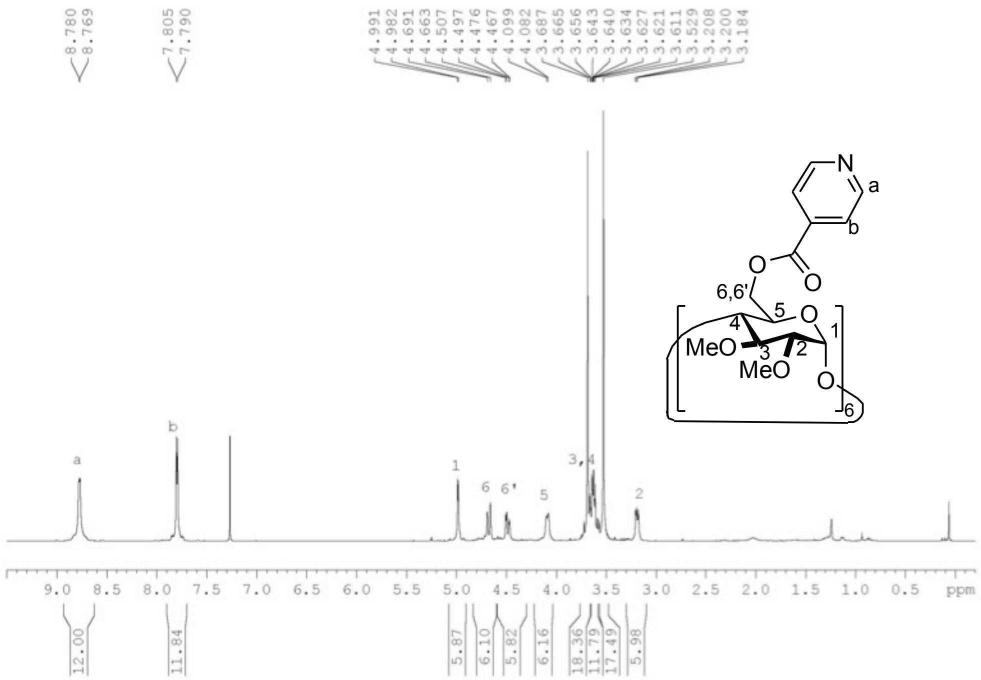

Fig. S1 The ^{1}H-NMR spectrum of **T6*** (400 MHz, $CDCl_3$).

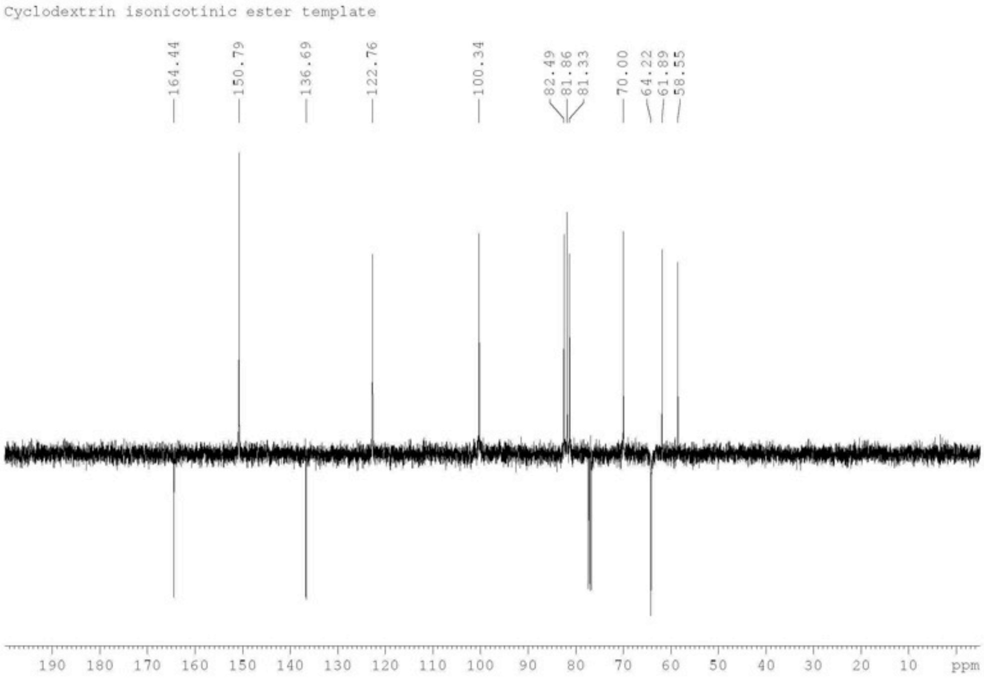

Fig. S2 The ^{13}C-NMR DEPT spectrum of **T6*** (100 MHz, $CDCl_3$).

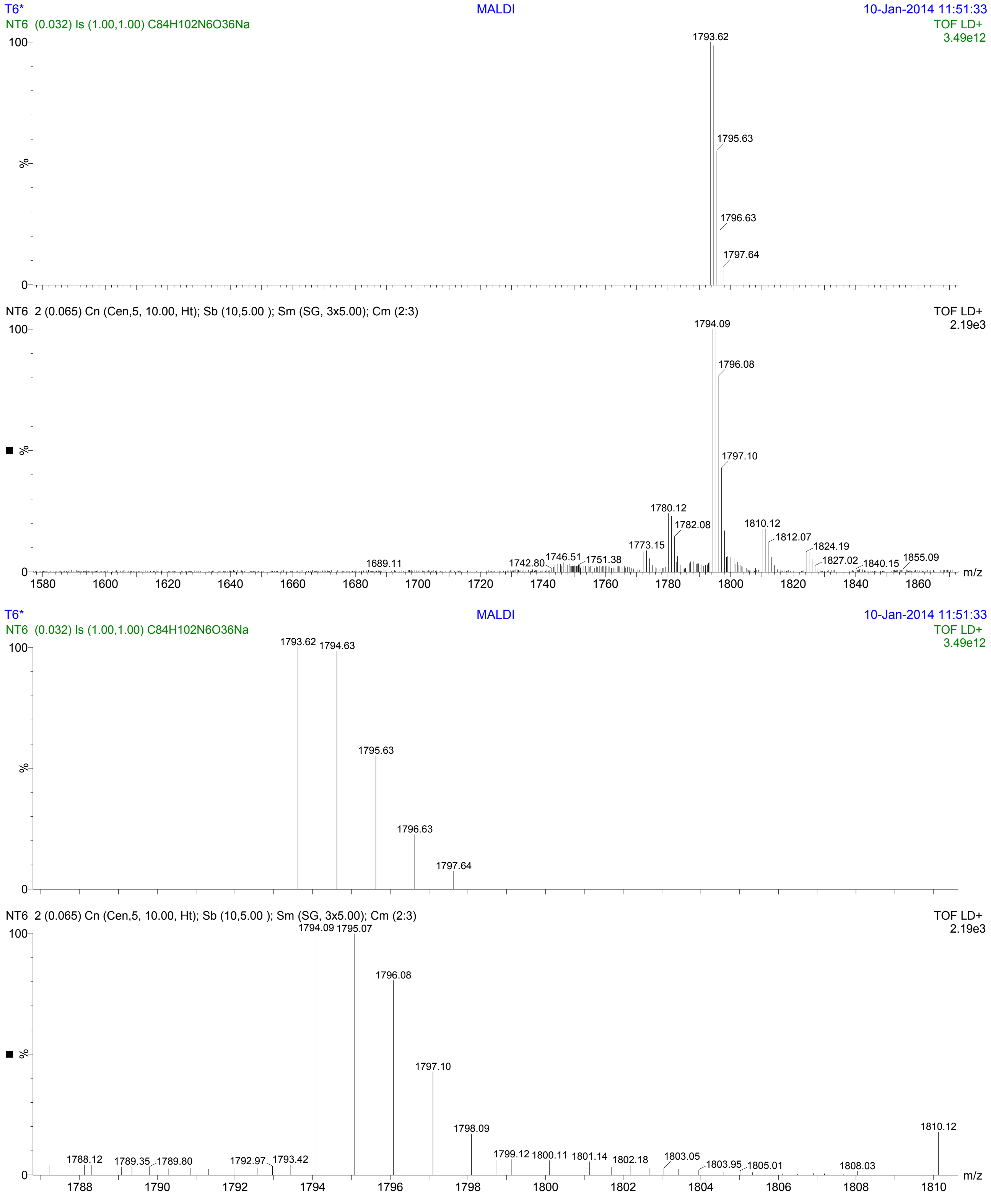

Fig. S3 The MALDI-MS spectrum of **T6*** (matrix: DCTB).

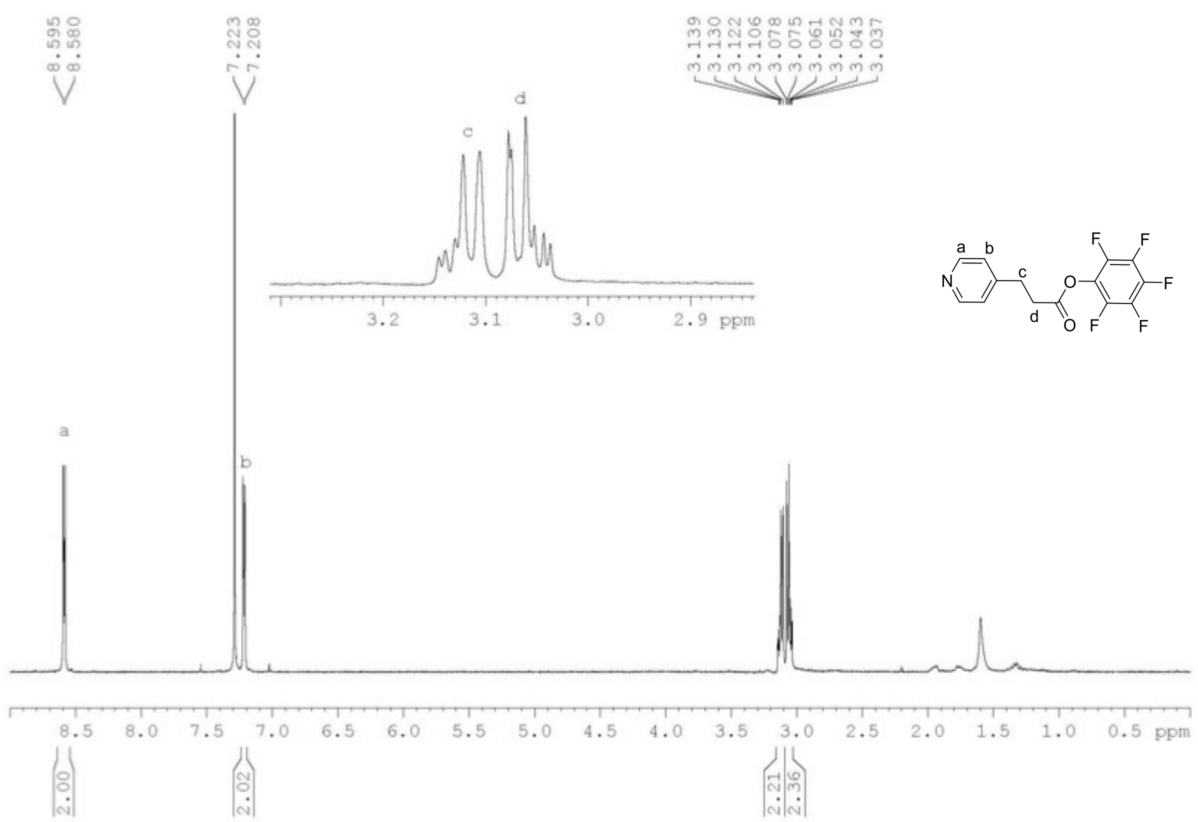

Fig. S4 The ^{1}H-NMR spectrum of 4-pyridinyl propanoic acid pentafluorophenol ester (400 MHz, $CDCl_3$).

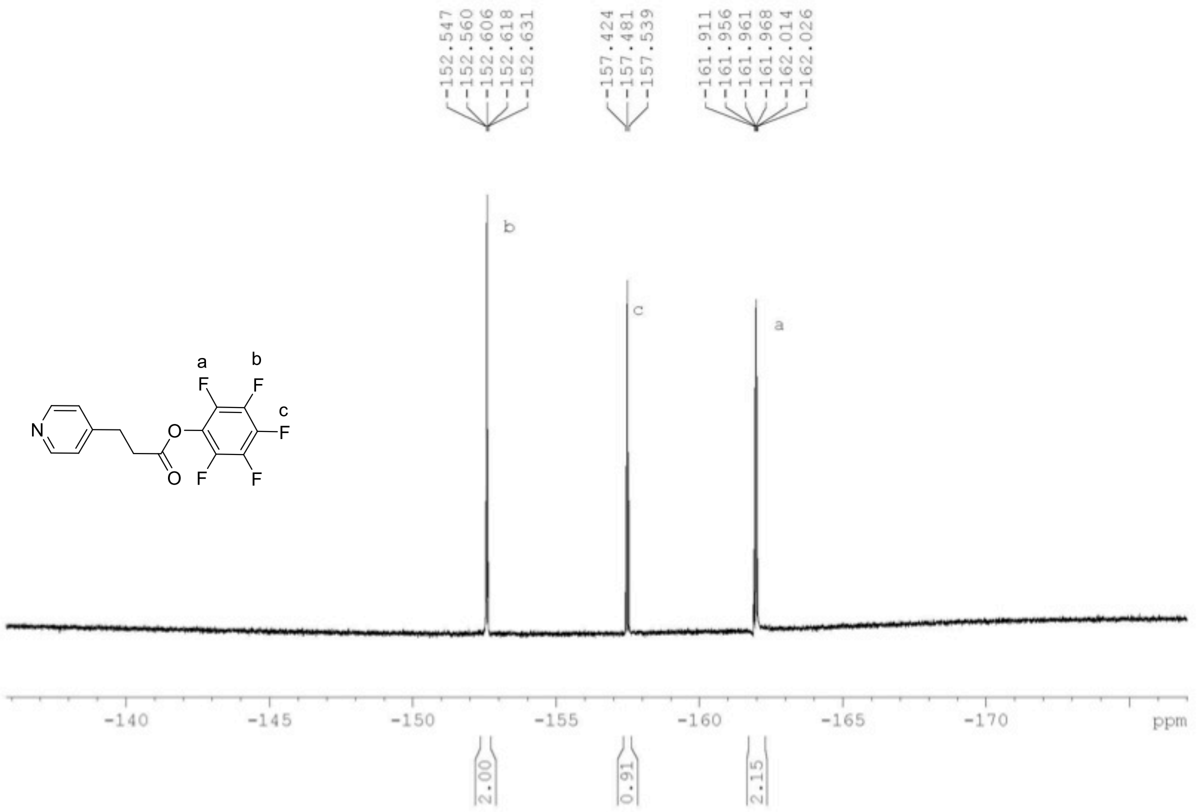

Fig. S5 The ^{19}F-NMR spectrum of 4-pyridinyl propanoic acid pentafluorophenol ester (377 MHz, $CDCl_3$).

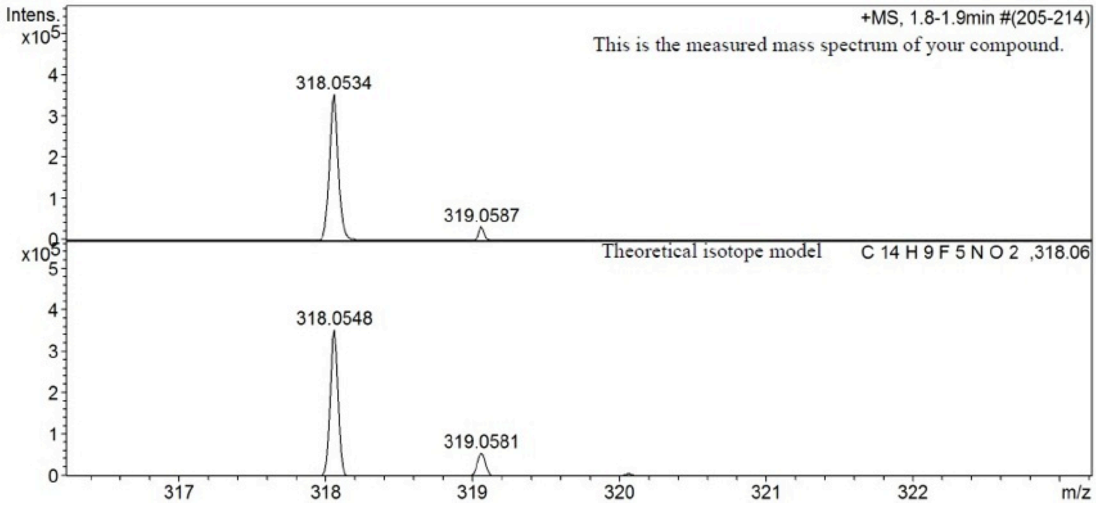

Fig. S6 The ESI-HDMS spectrum of 4-pyridinyl propanoic acid pentafluorophenol ester.

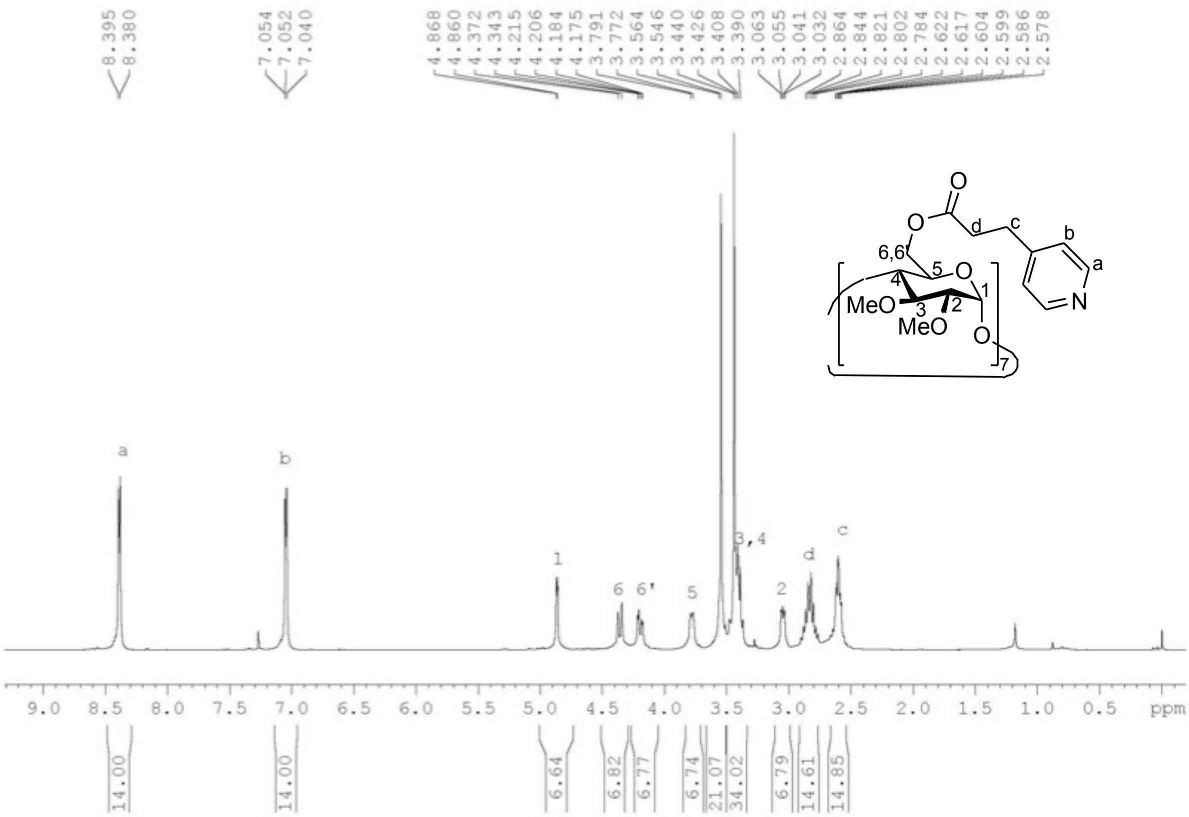

Fig. S7 The ^{1}H-NMR spectrum of **T7*** (400 MHz, $CDCl_3$).

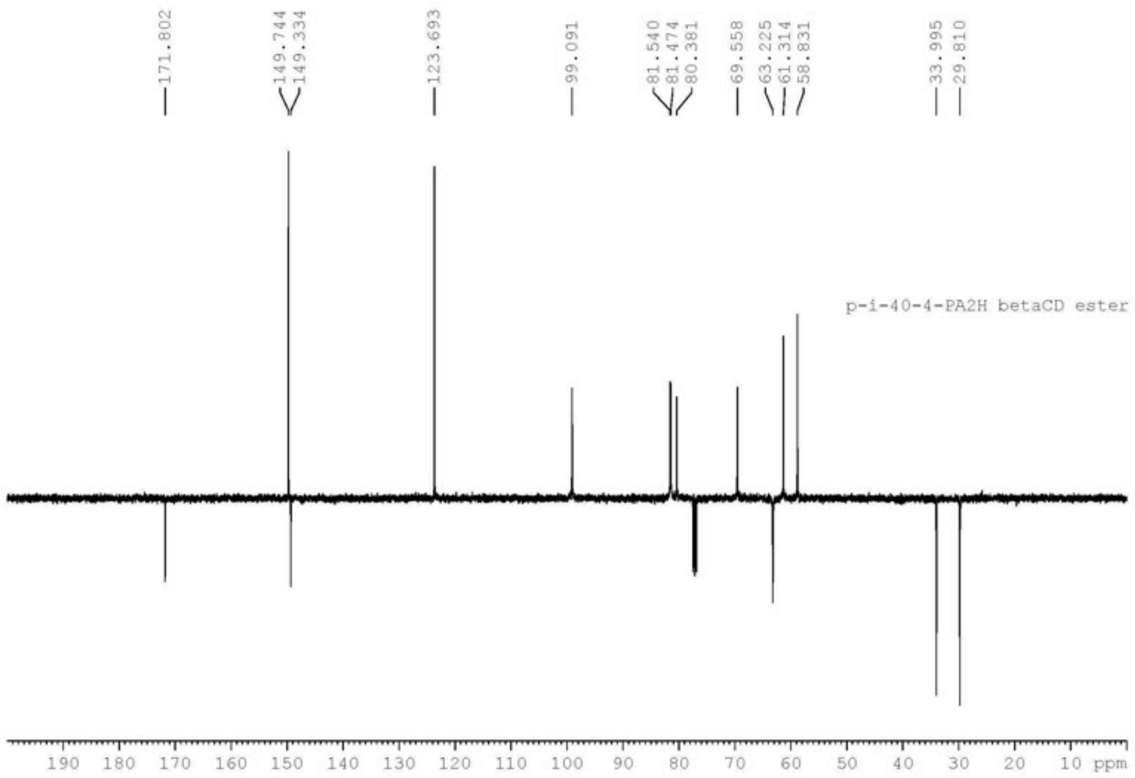

Fig. S8 The ^{13}C-NMR DEPT spectrum of **T7*** (100 MHz, $CDCl_3$).

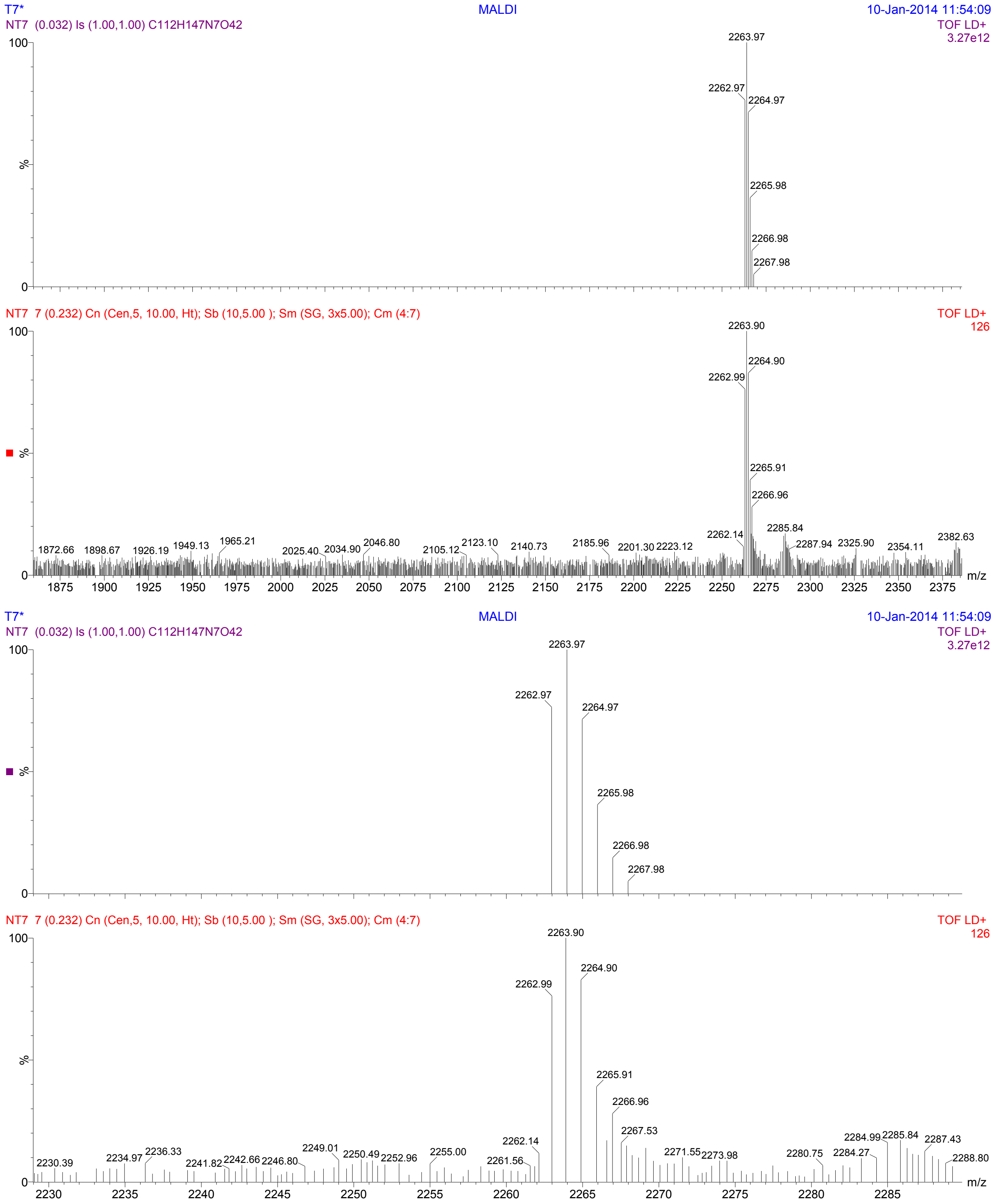

Fig. S9 The MALDI-MS spectrum of **T7*** (matrix: DCTB).

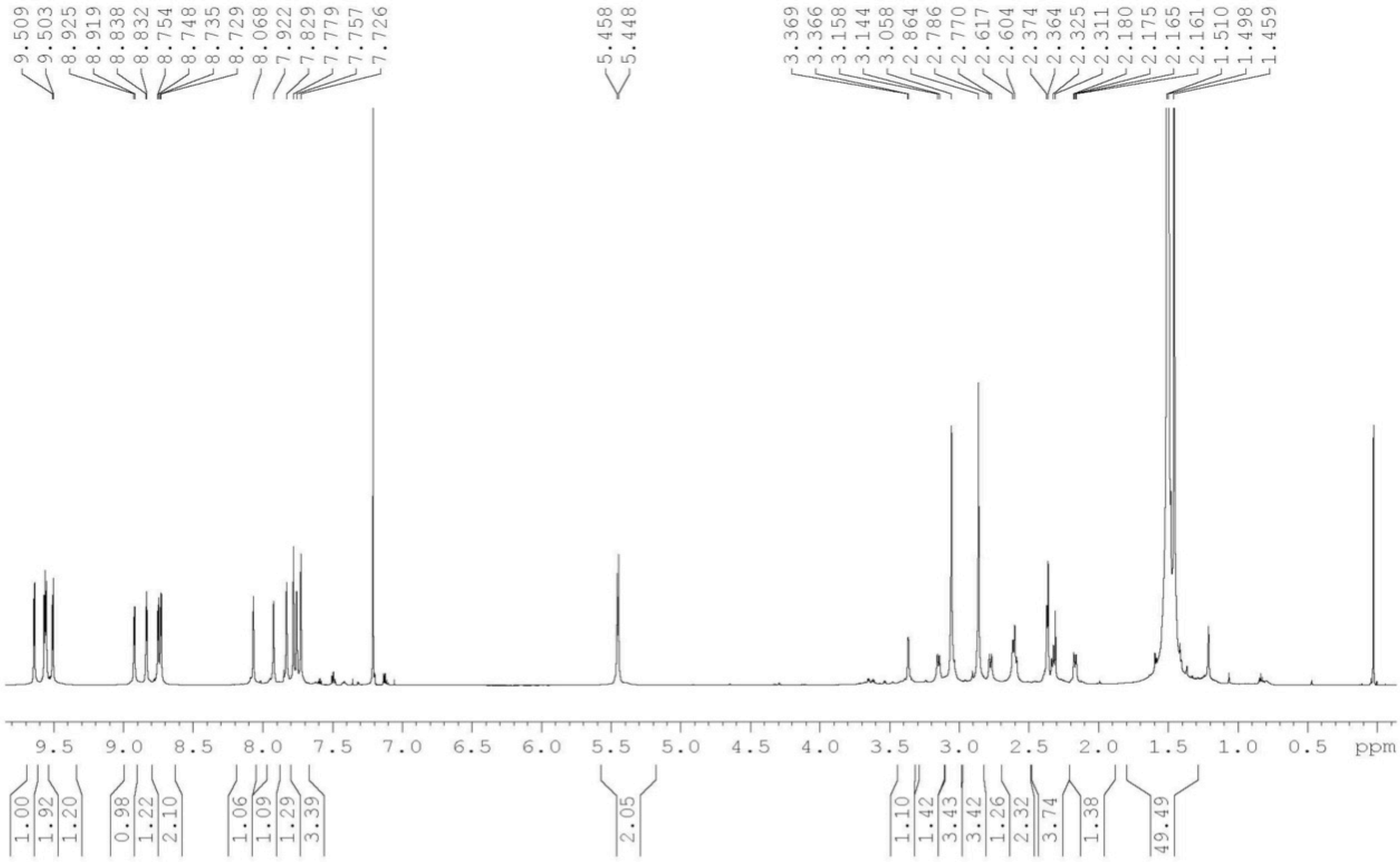

Fig. S10 The ^{1}H-NMR spectrum of ***c*-P6·T6*** (700 MHz, $CDCl_3$).

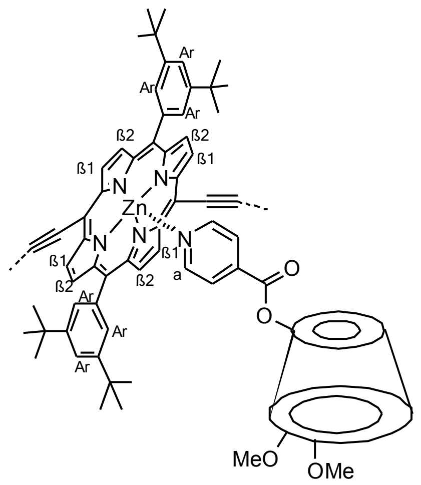

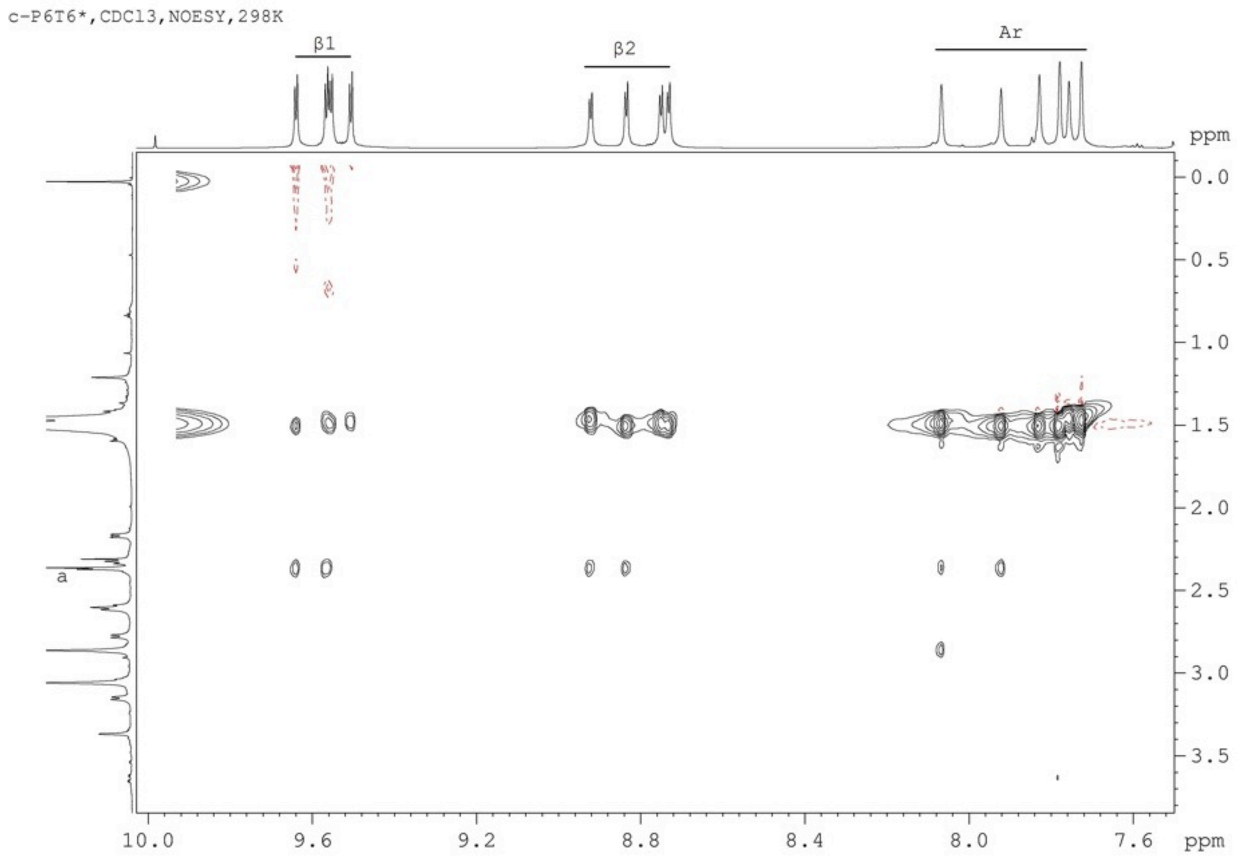

Fig. S11 The ^{1}H-^{1}H NOESY spectrum of ***c*-P6·T6*** (700 MHz, $CDCl_3$, correlation between pyridyl protons and protons on porphyrin, red 2D signals: negative signals). The eight β-protons and six aryl protons on each porphyrin unit are not equivalent.

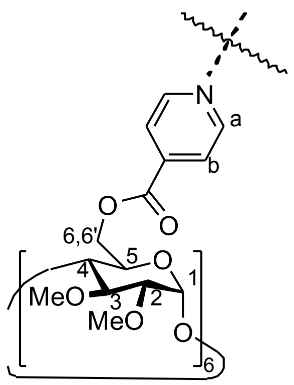

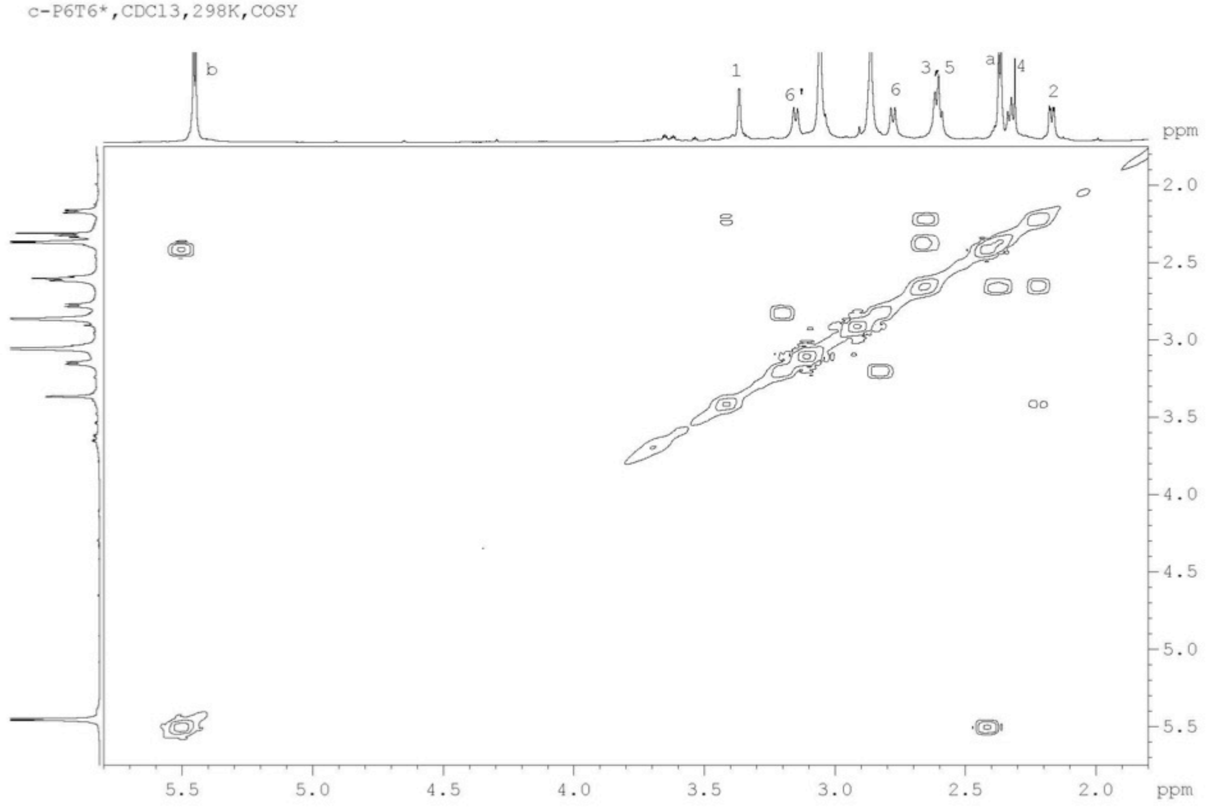

Fig. S12 The ^1H-^1H COSY spectrum of ***c*-P6·T6*** (500 MHz, $CDCl_3$, cyclodextrin region assignments).

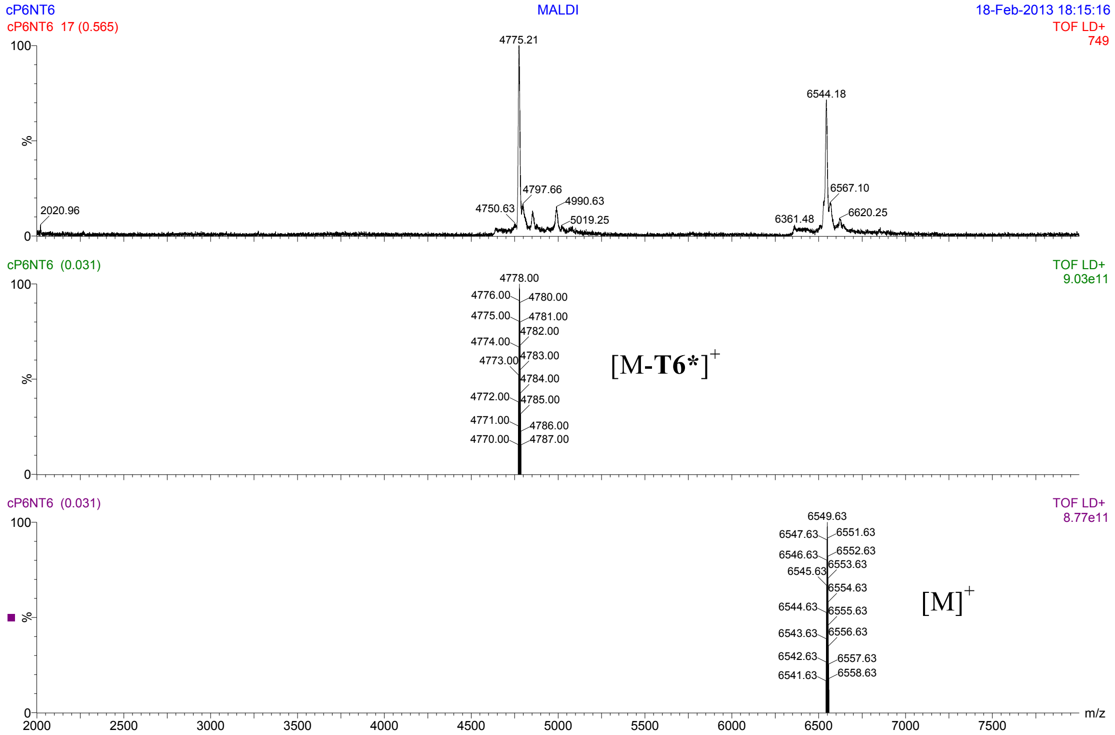

Fig. S13 The MALDI-MS spectrum of ***c*-P6·T6*** (matrix: DCTB).

The ^{1}H-NMR spectrum of ***c*-P12·(T6*)$_2$** is too complicated to be assigned in detail because this complex consists of a mixture of four diastereomers: the ***c*-P12** can exist in two enantiomeric conformations, each of which can bind two **T6*** units with their narrow primary rings both pointing the same way or with their rims pointing in opposite directions. However the structure of ***c*-P12·(T6*)$_2$** is supported by the fact that its ^{1}H NMR, ^{1}H DOSY spectra and UV-vis spectra are similar to those of the previously reported figure-of-eight complex ***c*-P12·(T6)$_2$**.[S6]

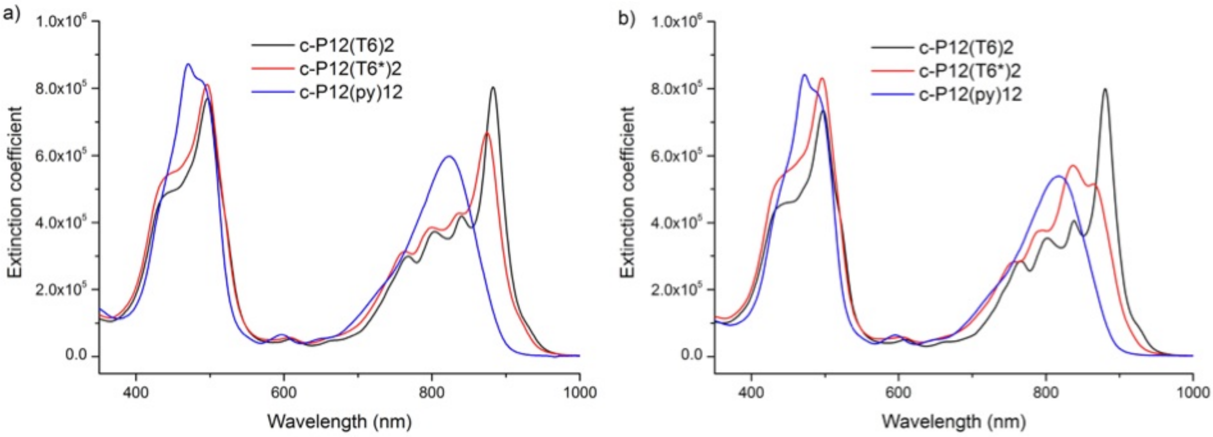

Fig. S14 Comparison of the extinction coefficient spectra (M^{-1} cm^{-1}, 298 K) of ***c*-P12·(T6)$_2$** (black curve), ***c*-P12·(T6*)$_2$** (red curve) and ***c*-P12** coordinated with 12 pyridine ligands (blue curve) in chloroform (a) and in toluene (b).

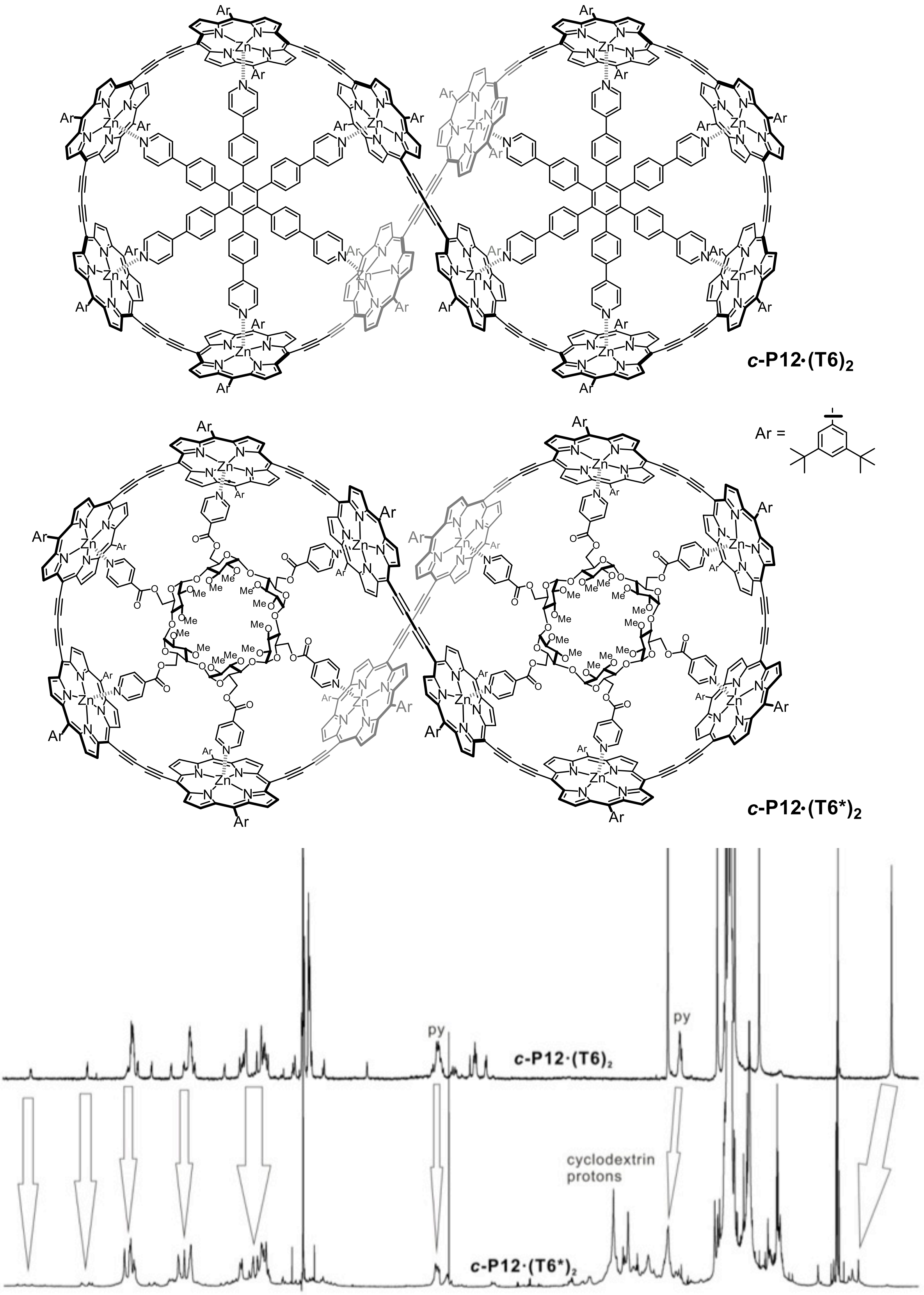

Fig. S15 Comparison of the ^{1}H-NMR spectra of ***c*-P12·(T6)$_2$** (top, 400 MHz, $CDCl_3$) and ***c*-P12·(T6*)$_2$** (bottom, 700 MHz, $CDCl_3$).

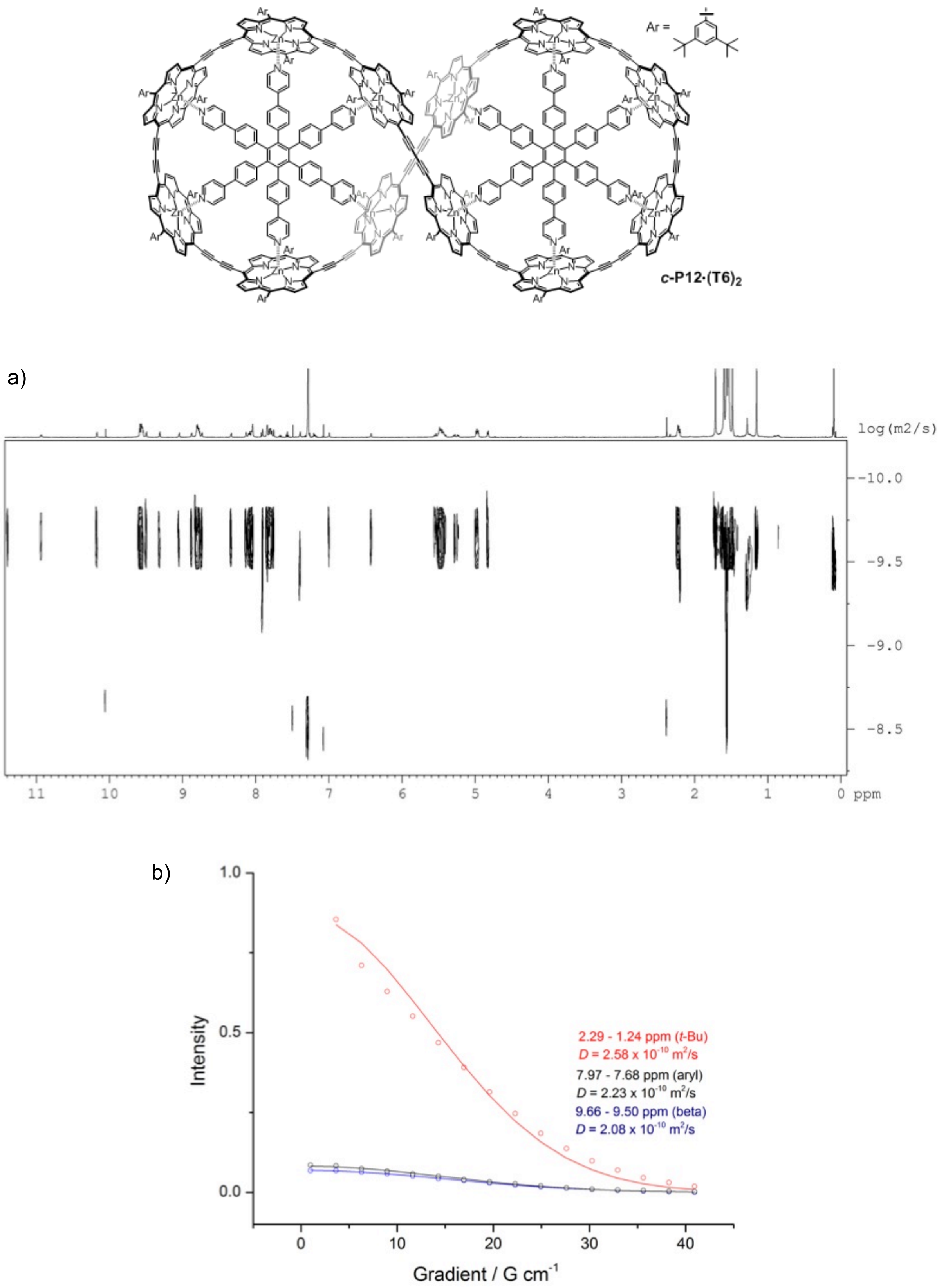

Fig. S16 a) ^{1}H DOSY spectrum of ***c*-P12·(T6)$_2$** ($CDCl_3$, 500 MHz, 298 K, Δ = 100 ms, δ = 4 ms) b) The representative protons from *tert*-butyl, aryl and β-position of porphyrin structures are used for the calculation of the diffusion coefficient of the molecule

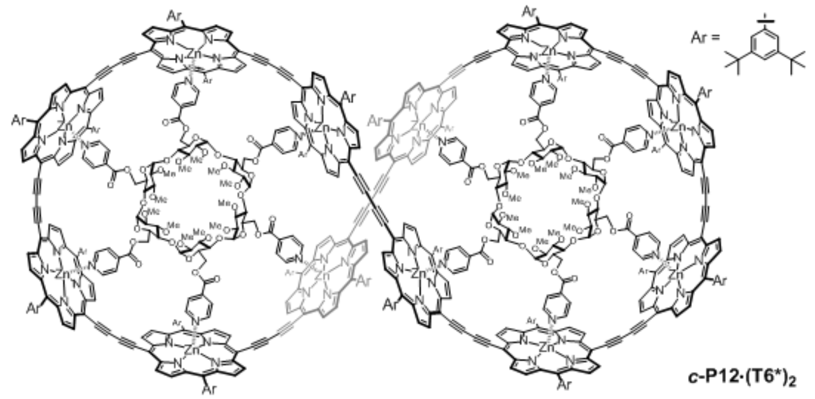

a)

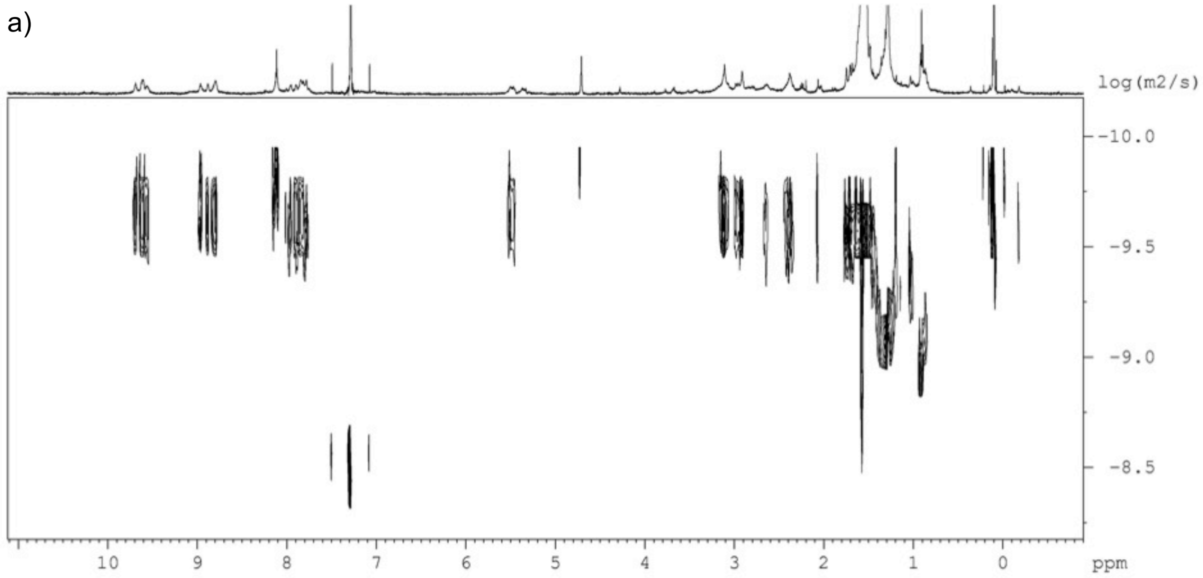

b)

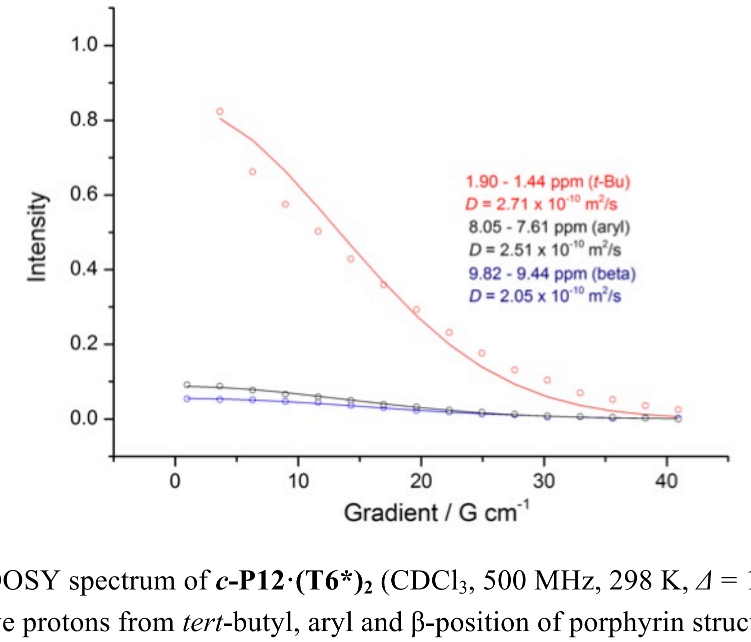

Fig. S17 a) ^{1}H DOSY spectrum of ***c*-P12·(T6*)$_2$** ($CDCl_3$, 500 MHz, 298 K, Δ = 100 ms, δ = 4 ms) b) The representative protons from *tert*-butyl, aryl and β-position of porphyrin structures are used for the calculation of the diffusion coefficient of the molecule. The corresponding protons have similar diffusion coefficients compared to the protons in ***c*-P12·(T6)$_2$**.

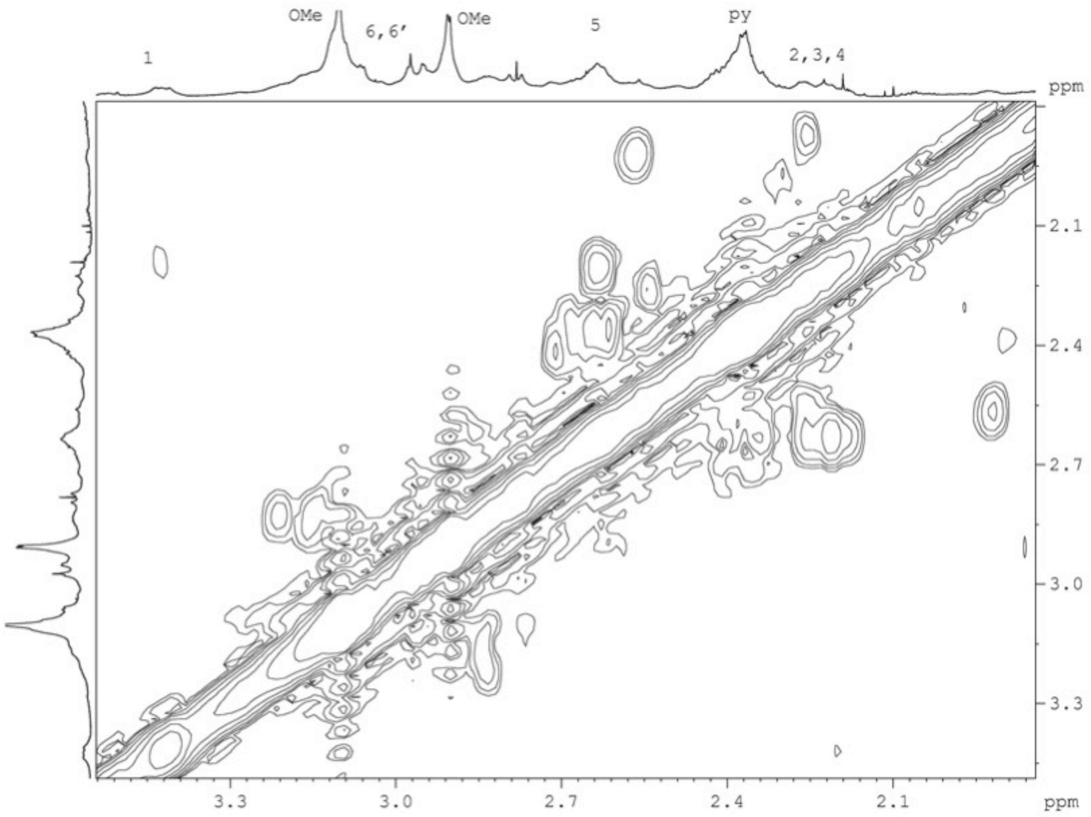

Fig. S18 ^{1}H-^{1}H COSY spectrum of ***c*-P12·(T6*)₂** and assignments of the cyclodextrin protons (aliphatic-aliphatic region, 700 MHz, $CDCl_3$).

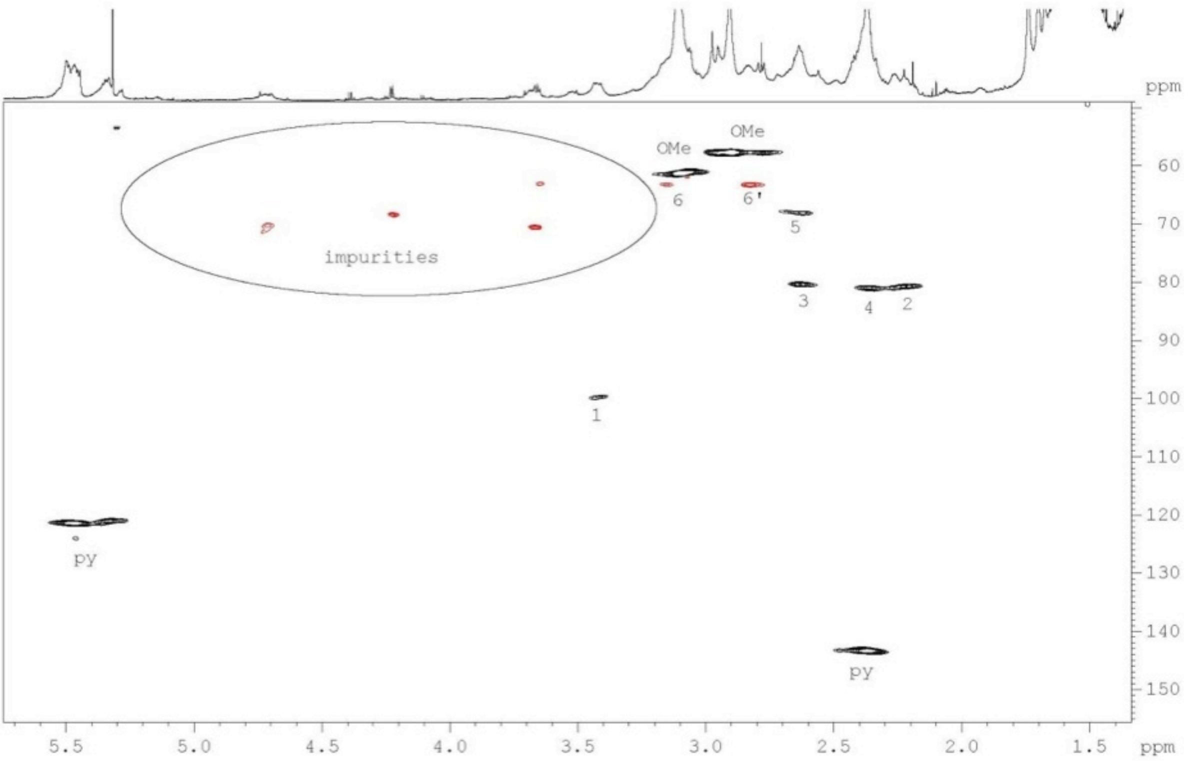

Fig. S19 HSQC spectrum of ***c*-P12·(T6*)$_2$** and assignments of the cyclodextrin and pyridyl protons (700 MHz, $CDCl_3$).

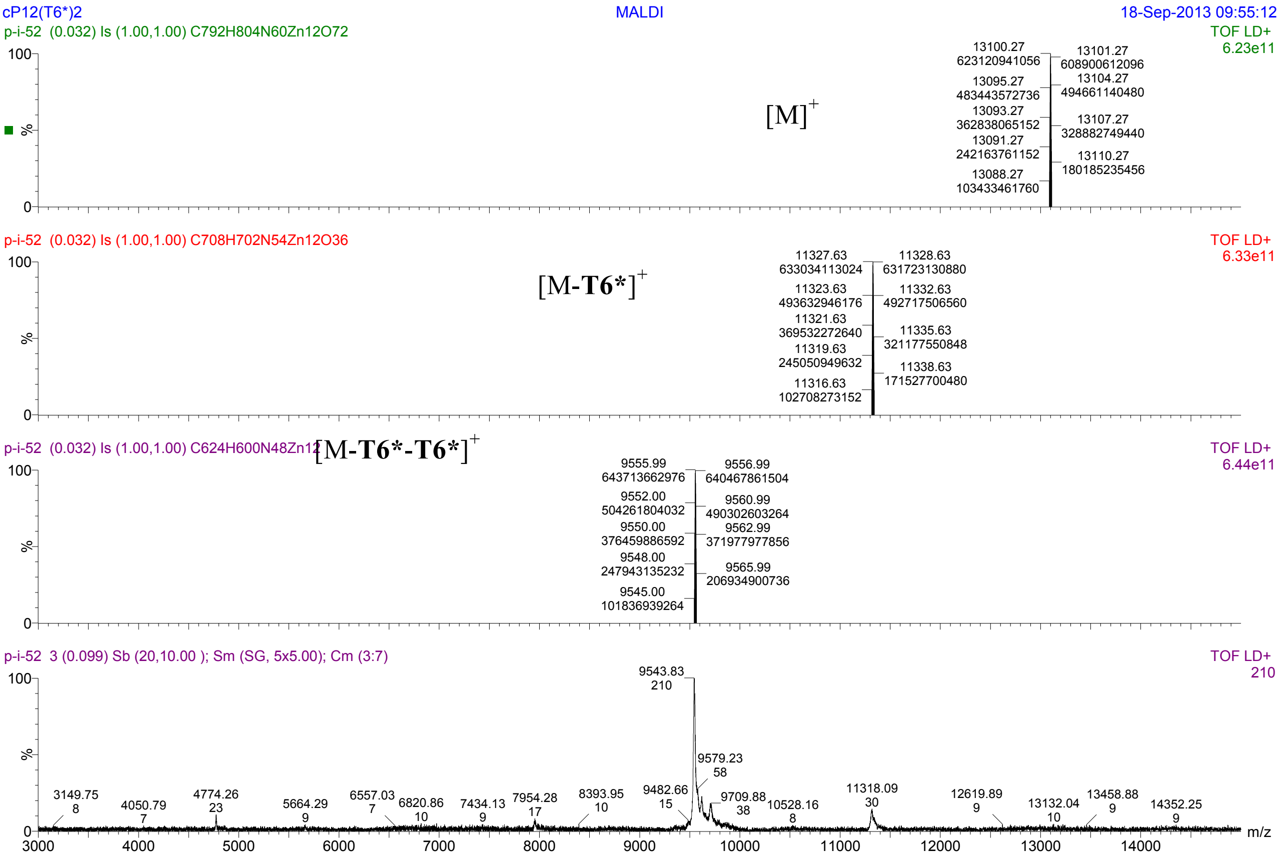

Fig. S20 The MALDI-MS spectrum of ***c*-P12·(T6*)$_2$** (matrix: DCTB).

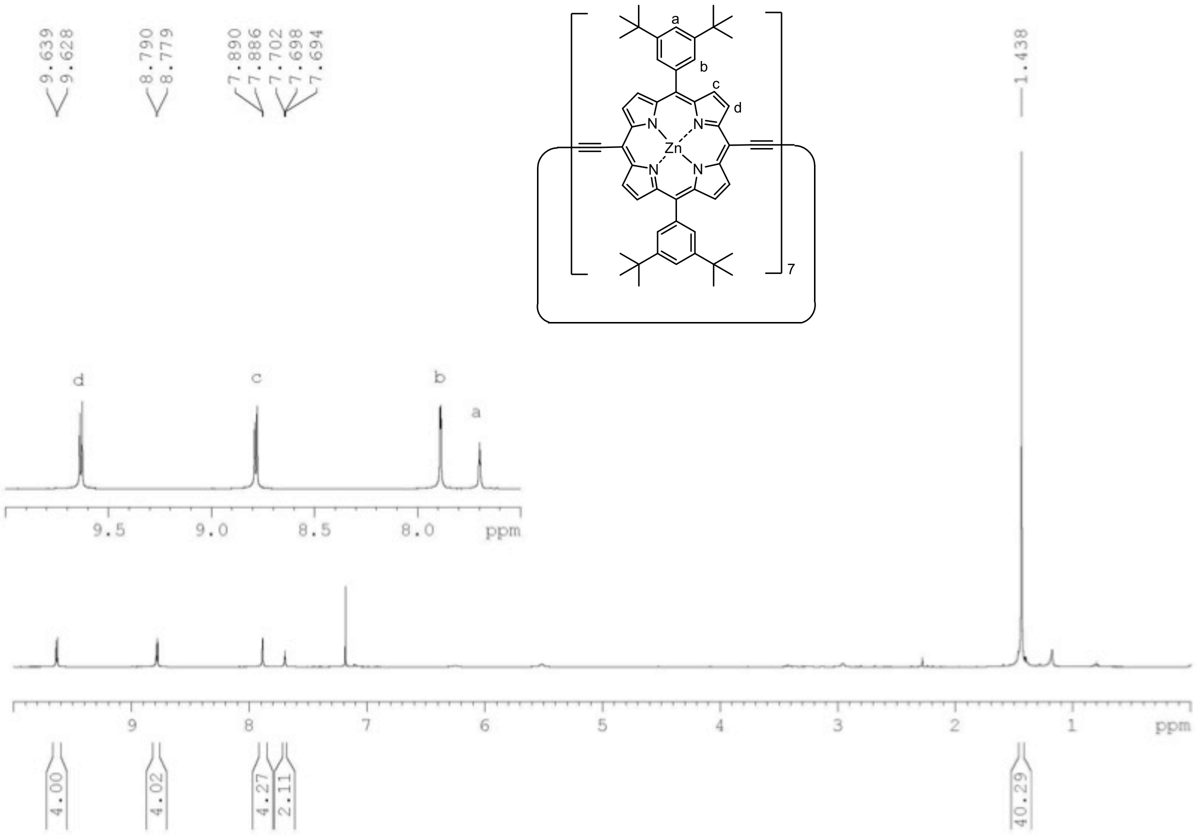

Fig. S21 The ^{1}H-NMR spectrum of ***c*-P7** (400 MHz, $CDCl_3$).

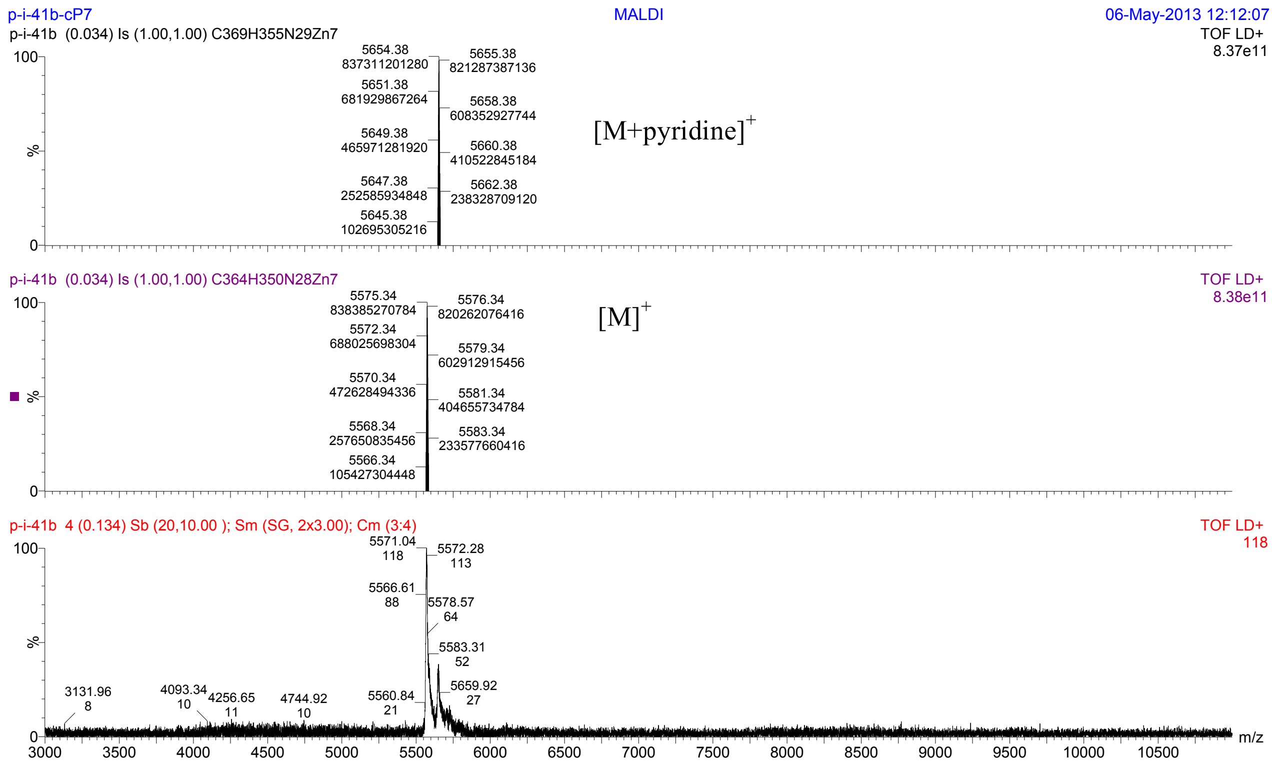

Fig. S22 The MALDI-MS spectrum of ***c*-P7** (matrix: DCTB).

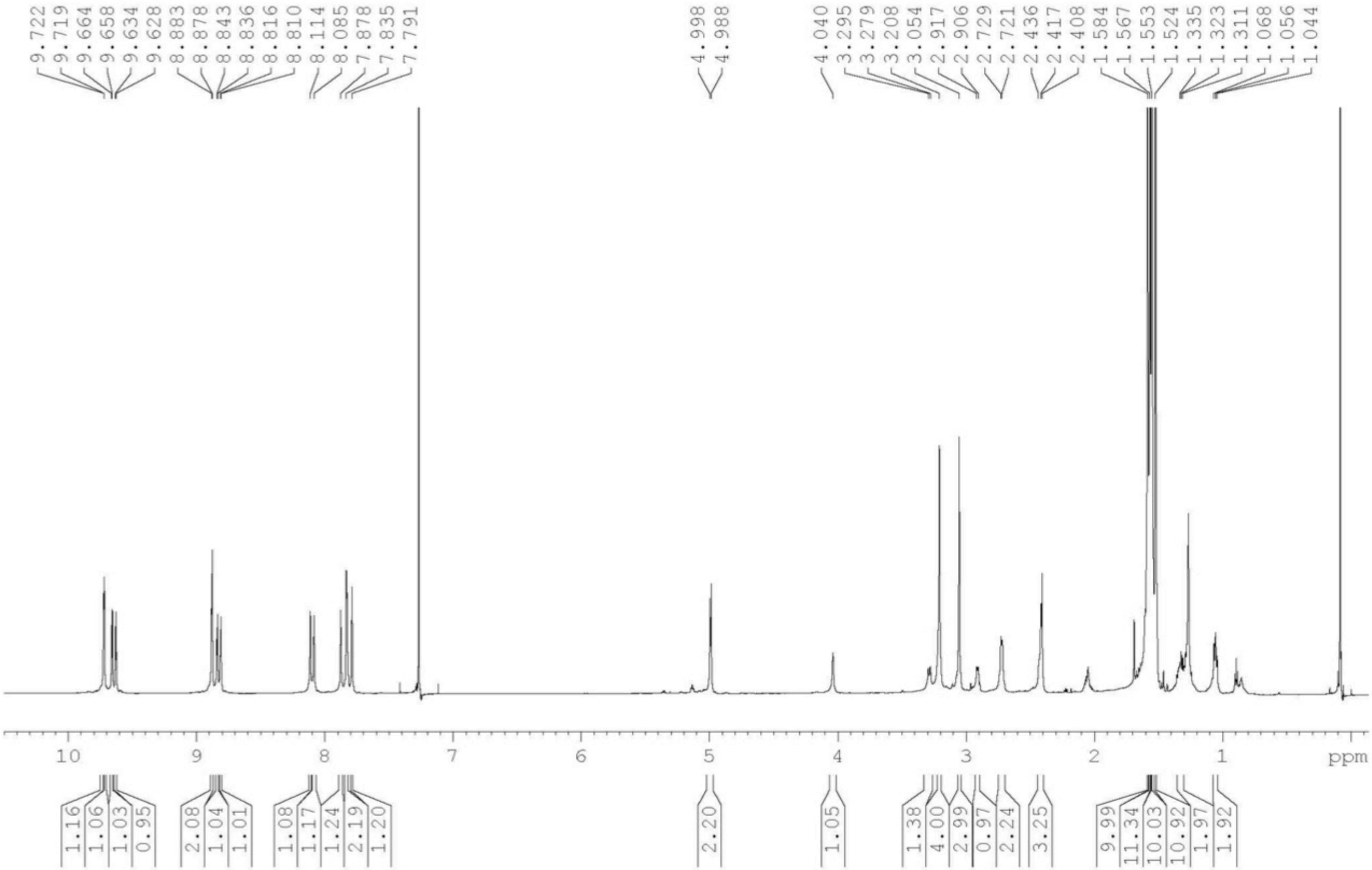

Fig. S23 The ^{1}H-NMR spectrum of ***c*-P7·T7*** (700 MHz, $CDCl_3$).

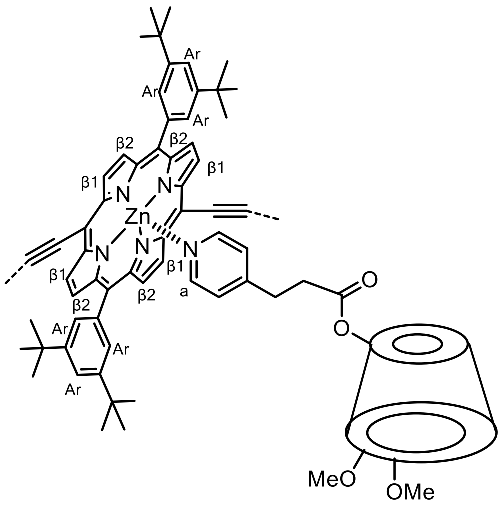

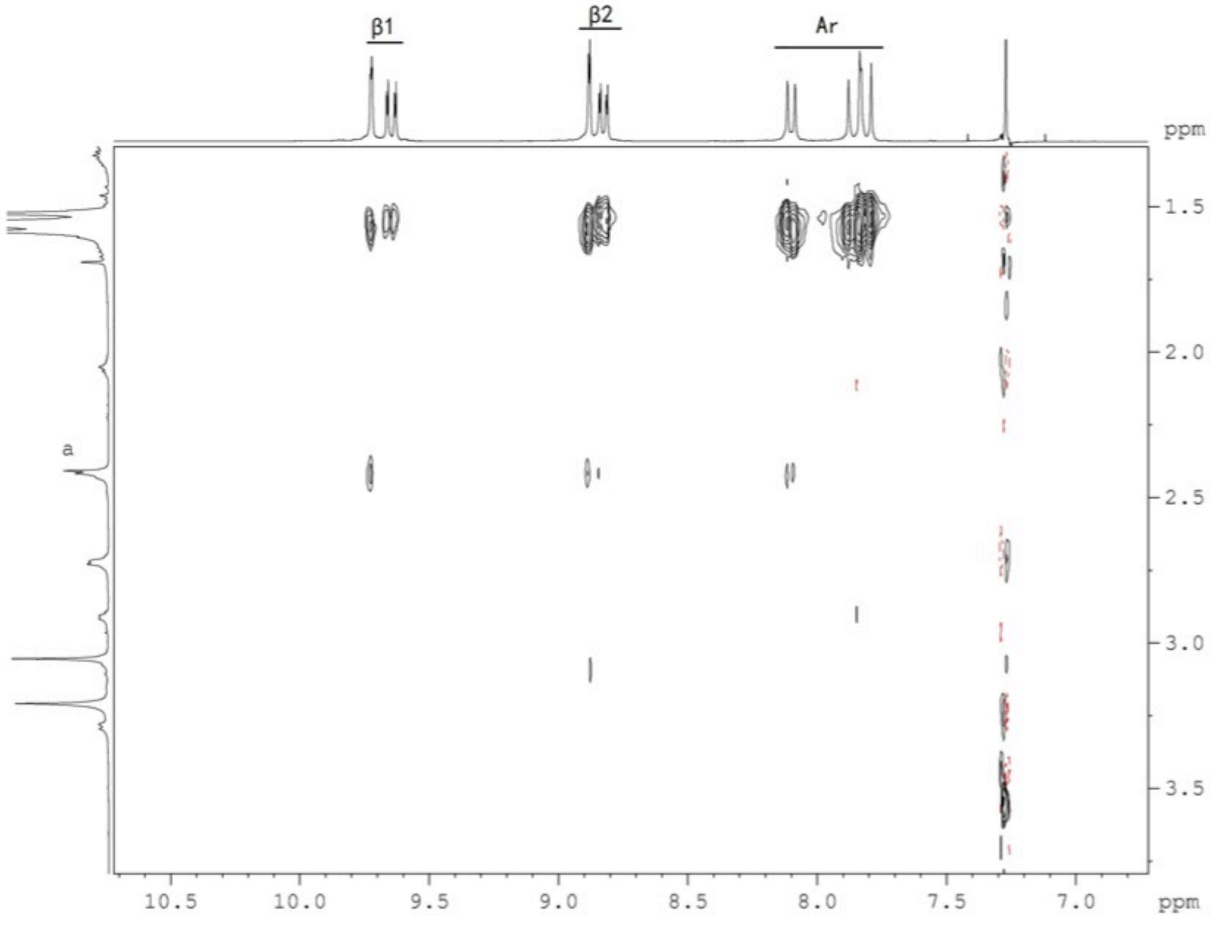

Fig. S24 The ^{1}H-^{1}H NOESY spectrum of ***c*-P7·T7*** (700 MHz, $CDCl_3$, correlation between pyridyl protons and protons on porphyrin, red 2D signals: negative signals). All the protons on the porphyrin units of ***c*-P7·T7*** are non-equivalent, as in ***c*-P6·T6***.

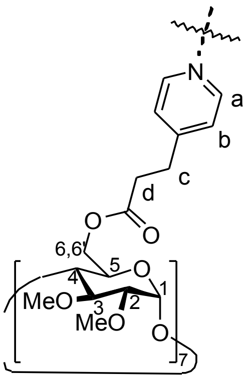

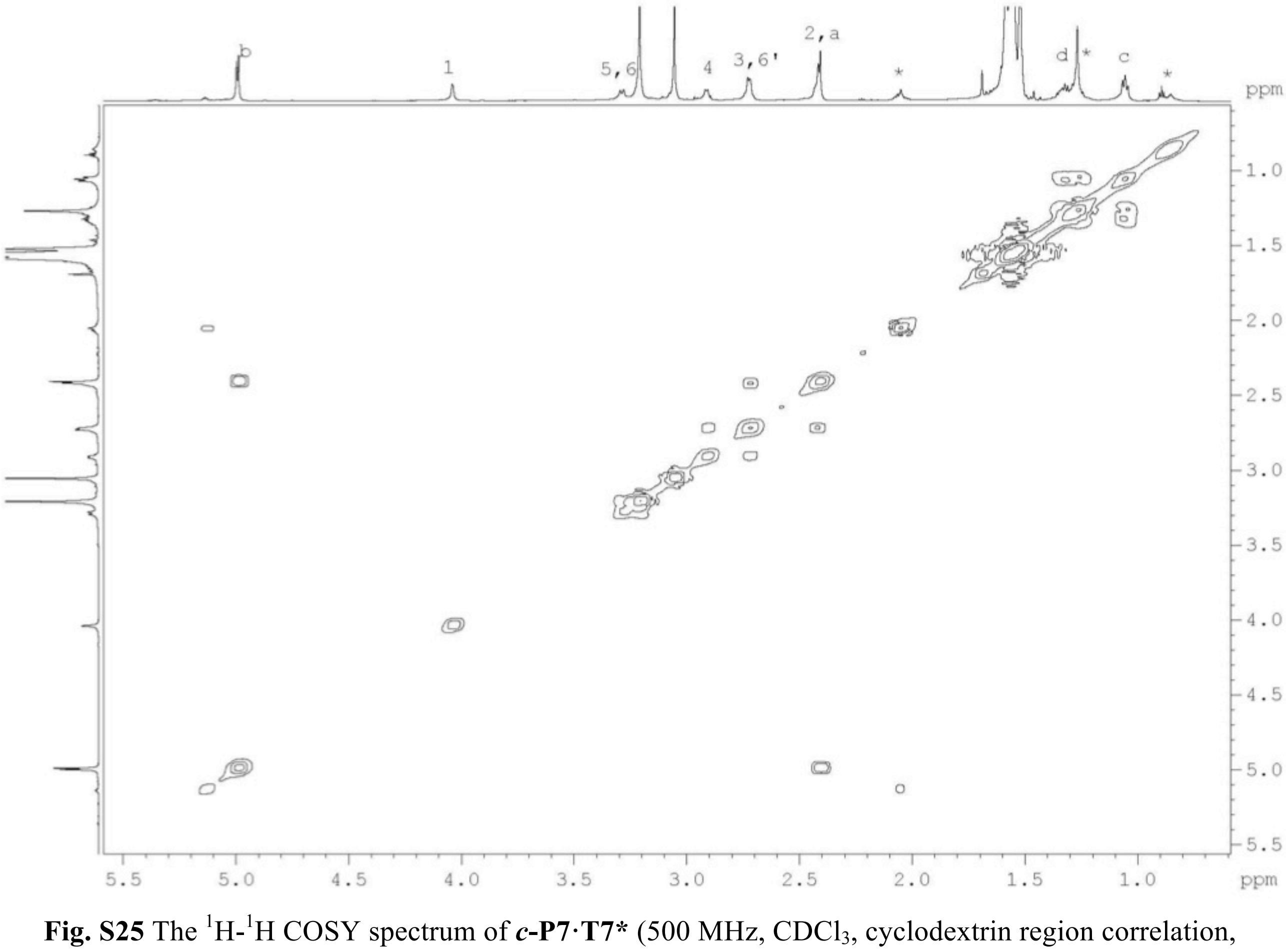

Fig. S25 The ^{1}H-^{1}H COSY spectrum of ***c*-P7·T7*** (500 MHz, $CDCl_3$, cyclodextrin region correlation, * indicates impurity signals).

a)

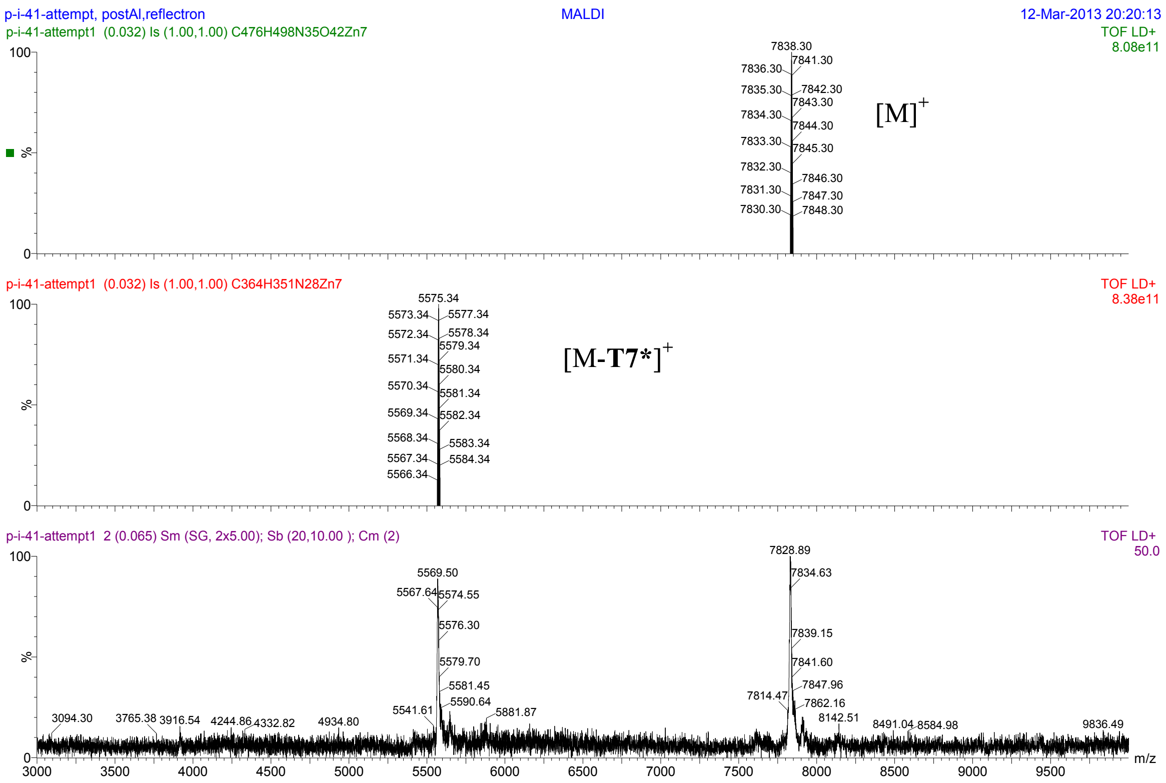

b)

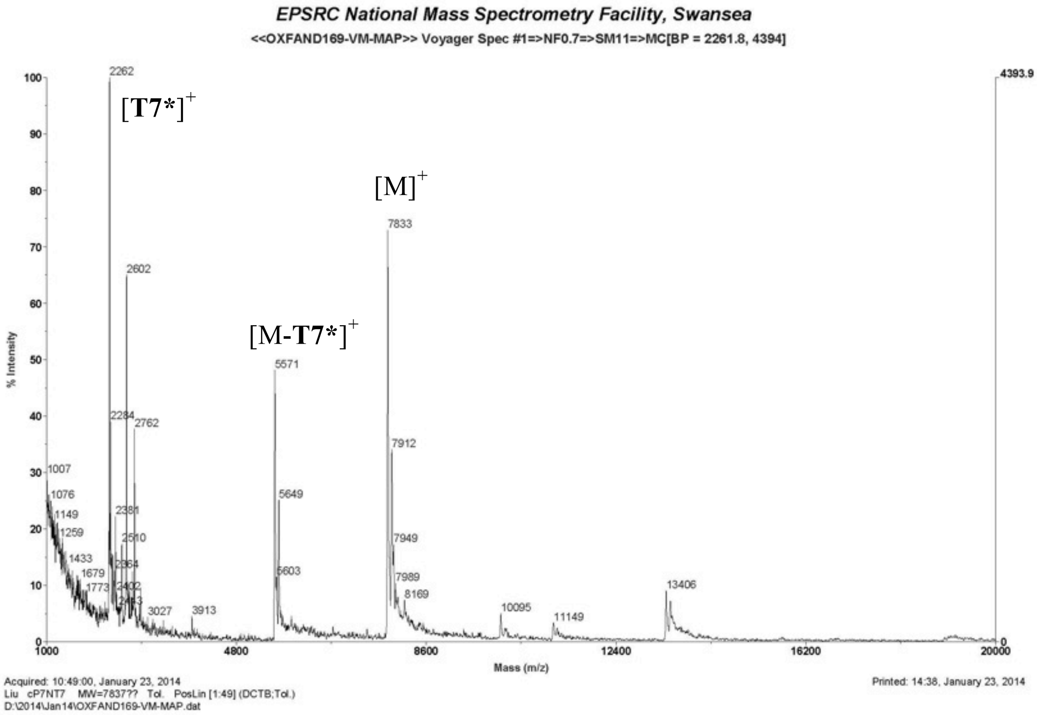

Fig. S26 The MALDI-MS spectra of ***c*-P7·T7*** (matrix: DCTB). a) Spectrum obtained from the MALDI service instrument at the Department of Chemistry, University of Oxford. b) Spectrum obtained from the EPSRC National Mass Spectrometry Facility, Swansea. The isotope pattern is not resolved, but the mass of the molecular ion matches well with that expected.

D. Circular Dichroism Spectroscopy

Circular dichroism spectra and the corresponding UV-Vis spectra were measured on a JASCO 815 instrument.

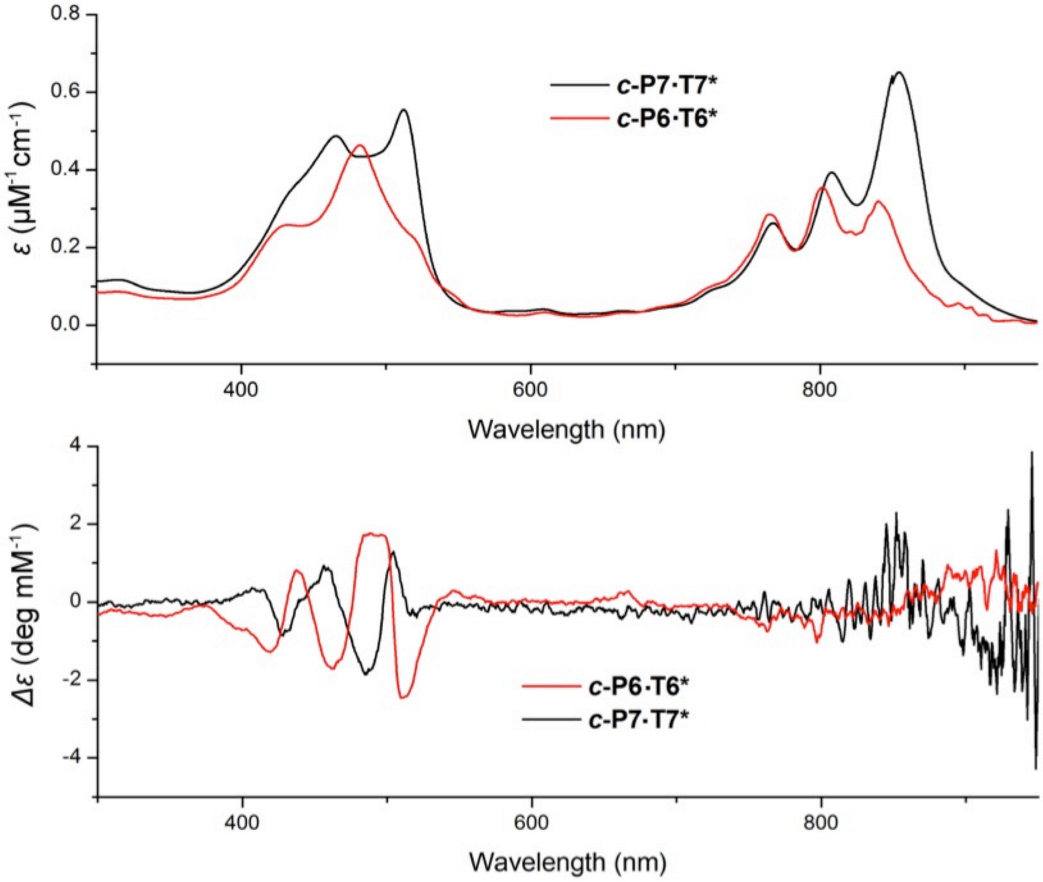

Fig. S27 Extinction coefficient (ε) and molar circular dichroism (Δε) spectra of ***c*-P6·T6*** and ***c*-P7·T7*** in toluene (298 K). The positive Coton effect (red trace) at 440 nm is slightly truncated in order to allow sensitive detection in the NIR.

E. Small-Angle X-ray Scattering Analysis of *c*-P6·T6* and *c*-P7·T7*

Synchrotron radiation SAXS data were collected using standard procedures on the I22 beamline at the Diamond Light Source (UK) equipped with a photon-counting detector. The beam was focused onto the detector placed at a distance of 1.25 m from the sample cell. The covered range of momentum transfer was $0.03 < q < 1.0$ Å^{-1} ($q = 4\pi\sin(\theta)/\lambda$, where 2θ is the scattering angle and $\lambda = 1.00$ Å is the X-ray wavelength). The data were normalized to the intensity of the transmitted beam; the scattering of the solvent was subtracted using an in-house program. To check for radiation damage during the SAXS experiment, the data were collected in 300 successive 1 s frames. Samples of ***c*-P6·T6*** and ***c*-P7·T7*** were dissolved in toluene at known concentrations (~10^{-4} M) and placed in a solution cell with mica windows (1 mm path length). Simulated scattering curves from molecular models were obtained by fitting to the experimental scattering data using the program CRYSOL.[S7] The program GNOM[S8] was used to calculate pair distribution functions (PDF) and radii of gyration (R_g) from experimental and simulated scattering data.

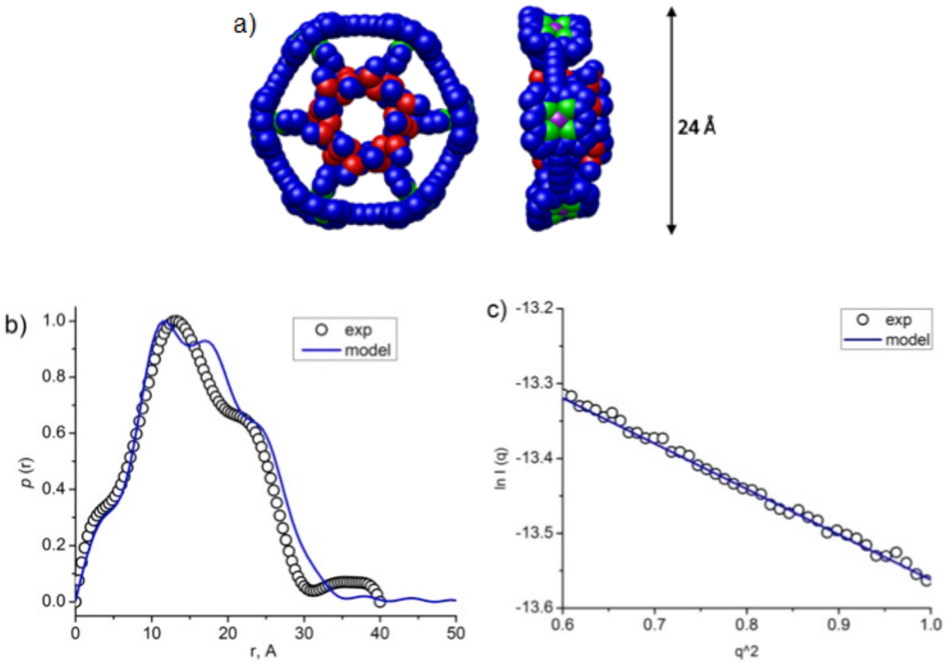

Fig. S28 SAXS analysis data of*c*-**P6·T6*** a) Model from molecular mechanics calculations; b) The pair-distribution function (PDF) from raw scattering data features characteristic 5 Å, 15 Å and 25 Å peaks close to the values in the model of *c*-**P6·T6*** (5 Å, 14 Å and 26 Å, respectively); c) The R_g obtained using the Guinier equation, 13.5 Å (the simulated PDF from the modeled structure $R_{g\,model}$ = 12.5 Å).

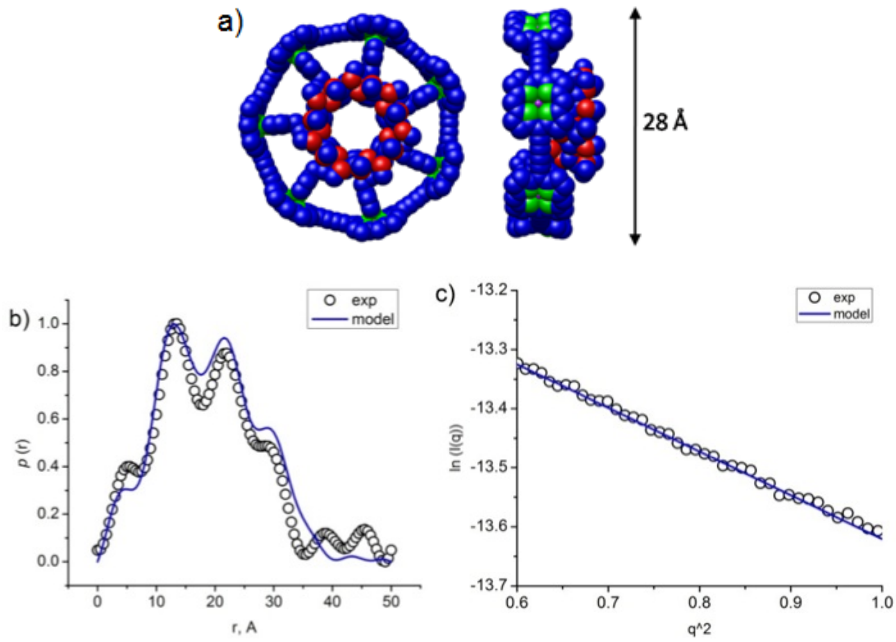

Fig. S29 SAXS analysis data of *c*-**P7·T7*** a) Model from molecular mechanics calculations; b) The pair-distribution function (PDF) from raw scattering data features characteristic 6 Å, 15 Å, 22 Å and 28.5 Å peaks close to the values in the model of *c*-**P7·T7*** (5 Å, 15 Å, 22 Å and 28 Å, respectively); c) The R_g obtained using the Guinier equation, 14.7 Å (the simulated PDF from the modeled structure $R_{g\,model}$ = 14.7 Å).

F. UV-Vis Titrations

F1. Titrations of Monodentate Ligands with Porphyrin Monomer P1'

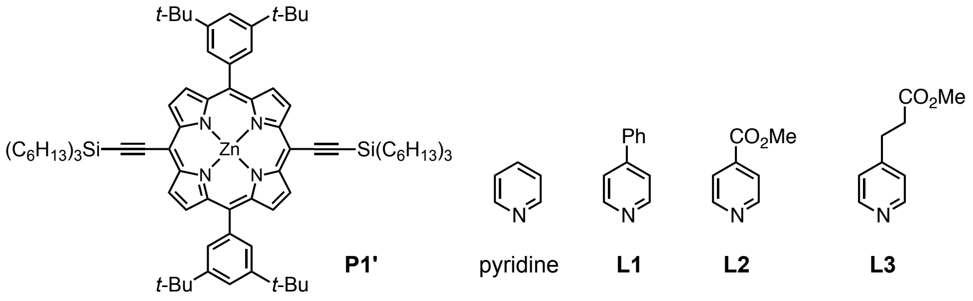

Fig. S30 Structures of monodentate ligands and reference porphyrin monomer **P1'**.

Titrations with porphyrin monomer **P1'** were carried out to compare the Lewis basicity of pyridine and ligands **L1**–**L2**. These results were not used in the calculation of effective molarities.

All titrations were performed in chloroform (passed through short basic alumina column to remove stabilizers) at 298 K. All titrations were carried out at constant porphyrin concentration by adding porphyrin to the ligand stock solution before titrations started. Titration curves were fitted to a 1:1 binding isotherm using the equation:

$$\frac{A-A_{initial}}{A_{\infty}-A_{initial}}=\left(\frac{(K_a([L]+[P]_0)+1)-\sqrt{(K_a([L]+[P]_0)+1)^2-4K_a^2[P]_0[L]}}{2K_a[P]_0}\right) \qquad \text{(S.1)}$$

where A is the observed absorption at a specific wavelength or difference of absorption at two wavelengths; $A_{initial}$ is the starting absorption at this wavelength or difference of absorption in these two wavelengths; A_{∞} is the asymptotic final absorption at this wavelength or difference of absorption in these two wavelengths; K_a is the association constant between ligand and porphyrin host; [L] is the concentration of ligand; $[P]_0$ is the concentration of porphyrin host.

The results are detailed in Table S1 and Figures S31–S38. In the spectra, the bold black lines represent starting points and the red lines represent terminal points.

Table S1. The 1:1 association constants of **P1'** and ligands (1:1 association constants in M^{-1}).

Ligand	Run 1	Run 2	Average	Spectra
pyridine	$(1.3\pm0.1) \times 10^4$	$(1.2\pm0.1) \times 10^4$	$(1.2\pm0.1) \times 10^4$	**Fig. S31, S32**
**L1**	$(7.8\pm0.2) \times 10^3$	$(8.5\pm0.1) \times 10^3$	$(8.1\pm0.4) \times 10^3$	**Fig. S33, S34**
**L2**	$(2.8\pm0.1) \times 10^3$	$(2.5\pm0.1) \times 10^3$	$(2.6\pm0.2) \times 10^3$	**Fig. S35, S36**
**L3**	$(1.2\pm0.1) \times 10^4$	$(1.4\pm0.1) \times 10^4$	$(1.3\pm0.1) \times 10^4$	**Fig. S37, S38**

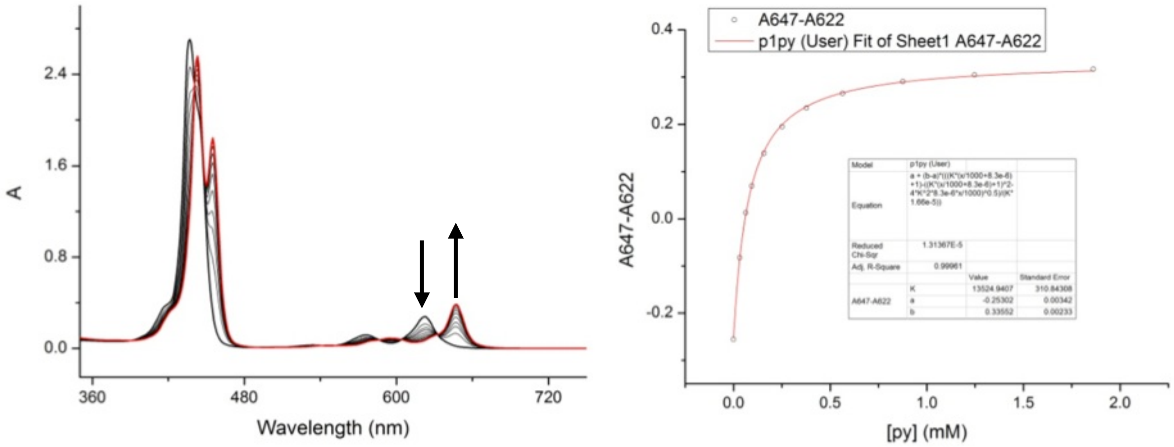

Fig. S31 UV-vis titration of pyridine and **P1'**, R^2 = 0.999.
(Run 1, $CHCl_3$, 298 K, [**P1'**] = 8.3 μM).

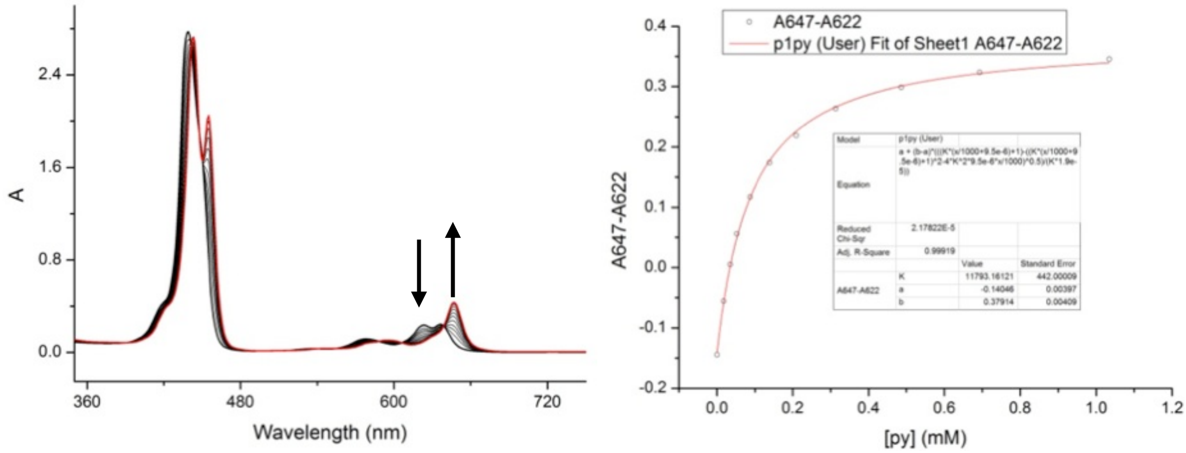

Fig. S32 UV/Vis titration of pyridine and **P1'**, R^2 = 0.999.
(Run 2, $CHCl_3$, 298 K, [**P1'**] = 9.5 μM).

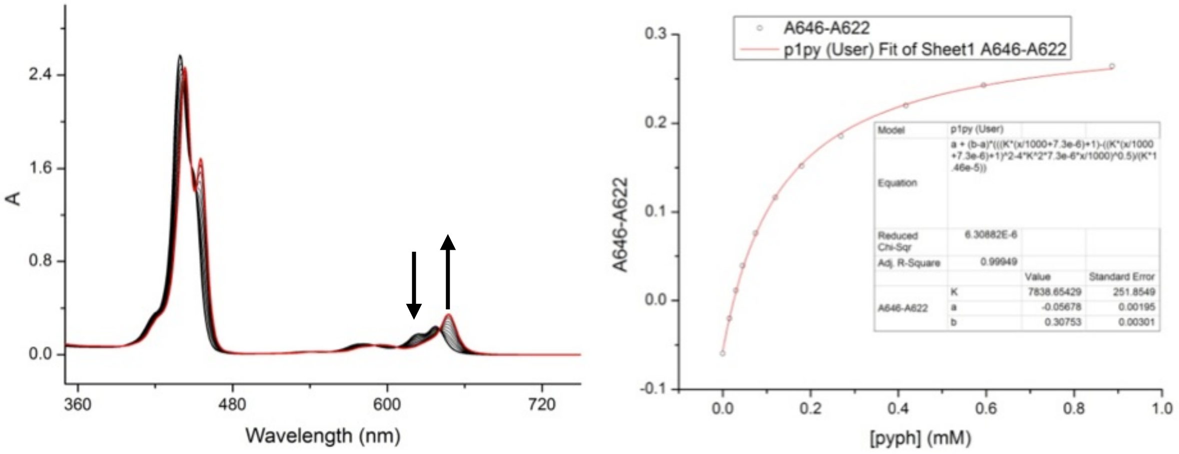

Fig. S33 UV/Vis titration of 4-phenylpyridine and **P1'**, R^2 = 0.999.
(Run 1, $CHCl_3$, 298 K, [**P1'**] = 7.3 μM).

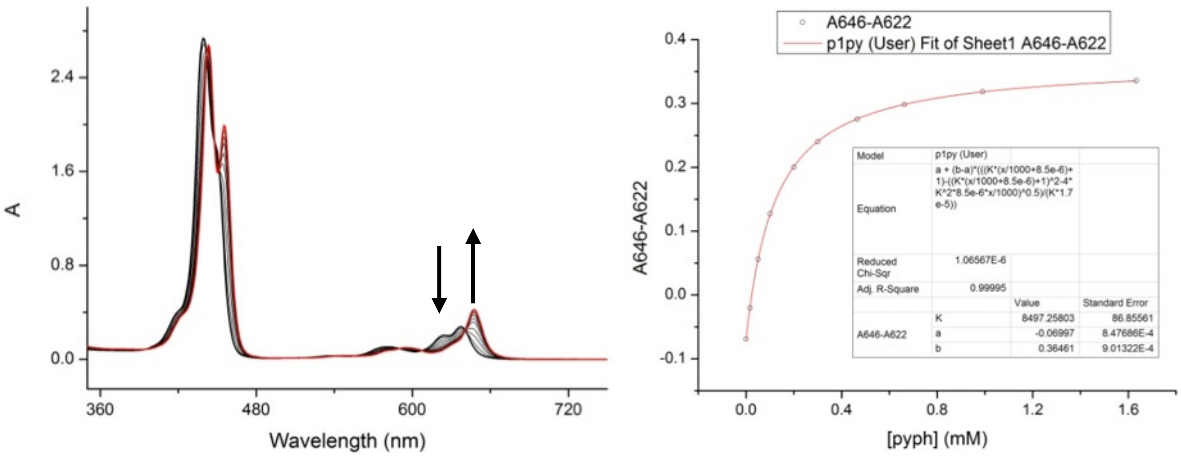

Fig. S34 UV/Vis titration of 4-phenylpyridine and **P1'**, R^2 = 0.999.
(Run 2, $CHCl_3$, 298 K, [**P1'**] = 8.5 μM).

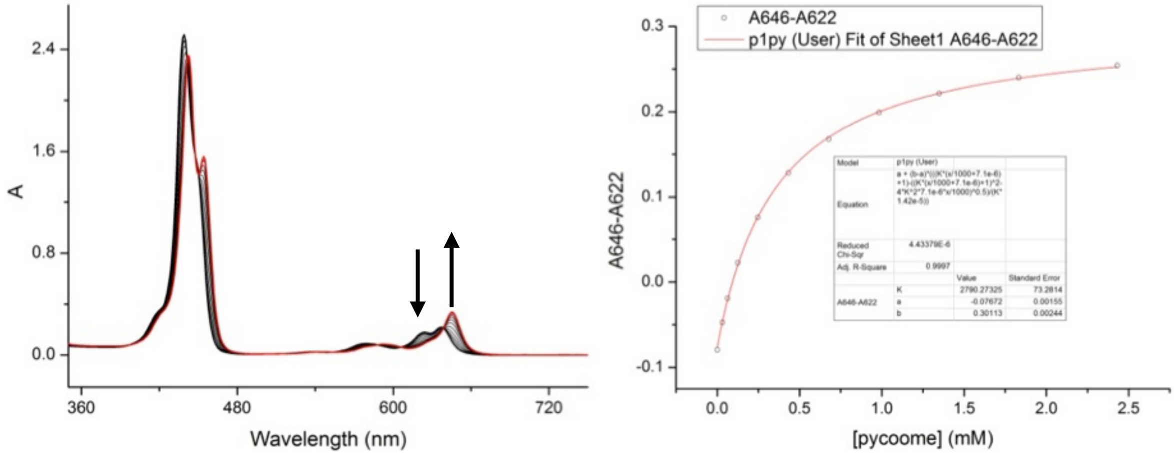

Fig. S35 UV/Vis titration of methyl isonicotinate and **P1'**, R^2 = 0.999.
(Run 1, $CHCl_3$, 298 K, [**P1'**] = 7.1 μM).

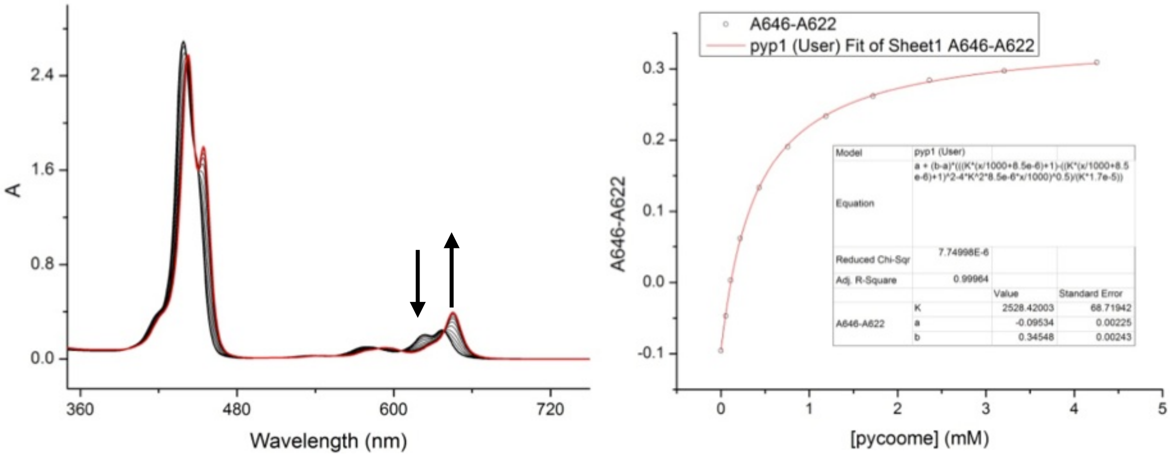

Fig. S36 UV/Vis titration of methyl isonicotinate and **P1'**, R^2 = 0.999.
(Run 2, $CHCl_3$, 298 K, [**P1'**] = 8.5 μM).

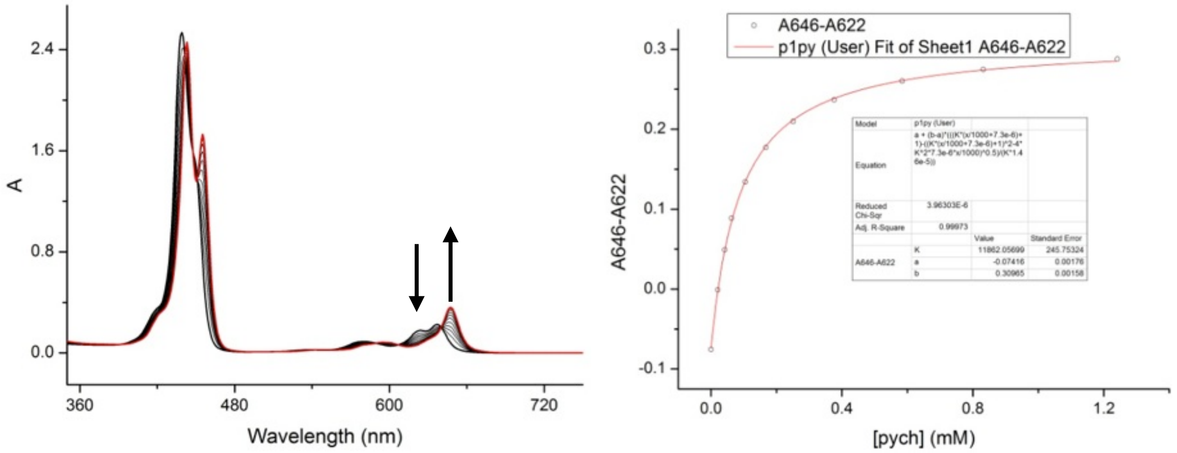

Fig. S37 UV/Vis titration of 4-pyridinepropanoic acid methyl ester and **P1'**, R^2 = 0.999. (Run 1, $CHCl_3$, 298 K, [**P1'**] = 7.3 μM).

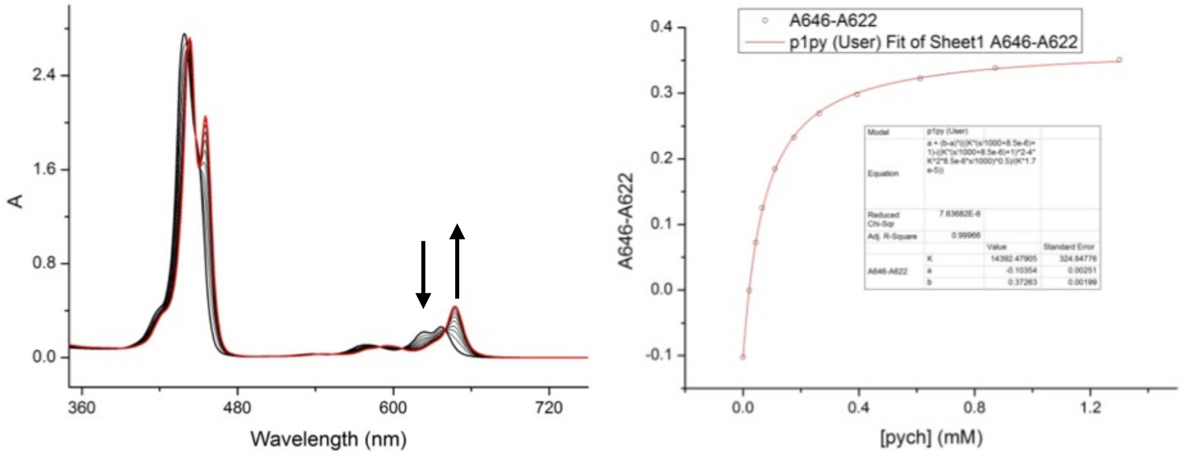

Fig. S38 UV/Vis titration of 4-pyridinepropanoic acid methyl ester and **P1'**, R^2 = 0.999. (Run 2, $CHCl_3$, 298 K, [**P1'**] = 8.5 μM).

F2. Titrations of Monodentate Ligands with Nanorings c-P6 and c-P7

***c*-P6** was titrated with the ligands shown in **Fig. S39** (quinuclidine, pyridine, 4-phenylpyridine and methyl isonicotinate) to measure their association constants. Unless stated otherwise, all the titrations were performed in chloroform (passed through short basic alumina column) at 298 K and the concentrations of porphyrin nanorings were 1.8 μM. All titrations were carried out at constant porphyrin concentration by adding porphyrin to the ligand stock solution before titrations started.

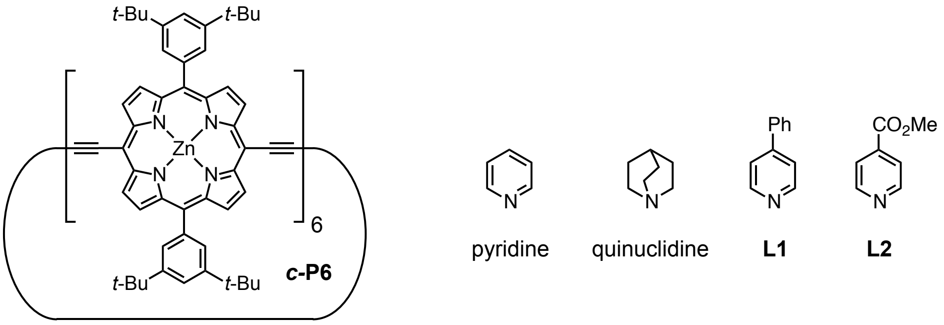

Fig. S39 Ligands used for measurements of reference association constants of ***c*-P6**.

***c*-P7** was titrated with the ligands shown in **Fig. S40** (quinuclidine, pyridine and 4-pyridinepropanoic acid methyl ester) to measure their association constants.

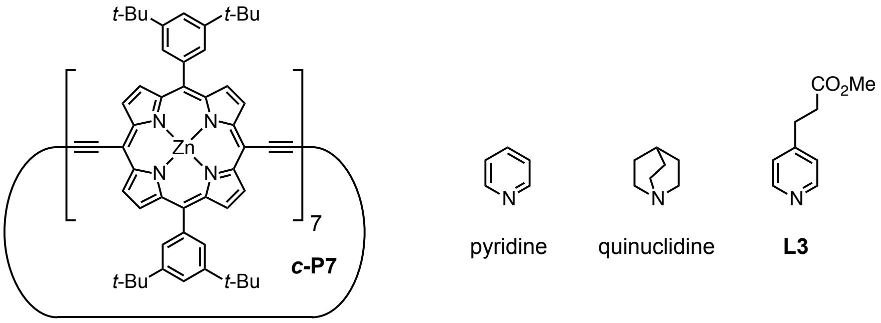

Fig. S40 Ligands used for measurements of reference association constants of ***c*-P7**.

The spectra for titration of ***c*-P6** with monodentate ligands are slightly nonisobestic, which can lead to uncertainly in the association constant. This problem was minimized by taking the differences between absorption at 828 nm (the right shoulder of titration terminal spectra) and absorption at 736 nm (the left shoulder of titration starting spectra) to give the largest yet reliable change in absorption. The results were found to be self-consistent and consistent with previously published data.[S9]

Titration curves were fitted to a 1:1 binding isotherm using the equation (S.1) similar to Section F1. However, the $[P]_0$ is a 6-fold multiply for the concentration of ***c*-P6** and 7-fold multiply for the concentration of ***c*-P7**, as they have multiple porphyrin units inside each molecule.

The results are listed in the tables and figures below. In the spectra, the bold black lines represent starting points and the red lines represent terminal points.

Table S2 The association constants of ***c*-P6** and ligands (1:1 association constants in M^{-1}).

Ligand	Run 1	Run 2	Average	Comments	Spectra
quinuclidine	$(3.2\pm0.1) \times 10^5$	$(2.8\pm0.1) \times 10^5$	$(3.0\pm0.2) \times 10^5$	In Run 2, [***c*-P6**] = 1.9 μM	**Fig. S41, S42**
pyridine	$(1.1\pm0.1) \times 10^4$	$(8.0\pm0.3) \times 10^3$	$(9.5\pm1.5) \times 10^3$		**Fig. S43, S44**
**L1**	$(8.4\pm0.3) \times 10^3$	$(8.4\pm0.3) \times 10^3$	$(8.4\pm0.3) \times 10^3$	In Run 2, [***c*-P6**] = 1.6 μM	**Fig. S45, S46**
**L2**	$(1.4\pm0.1) \times 10^3$	$(1.2\pm0.1) \times 10^3$	$(1.3\pm0.1) \times 10^3$	In Run 2, [***c*-P6**] = 1.6 μM	**Fig. S47, S48**

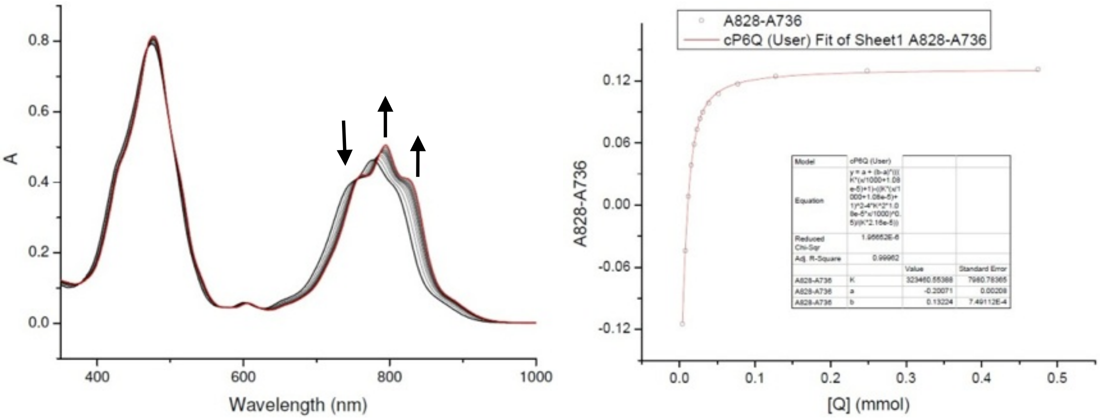

Fig. S41 UV-vis titration of quinuclidine and ***c*-P6**, R^2 = 0.999.
(Run 1, $CHCl_3$, 298 K, [***c*-P6**] = 1.8 μM).

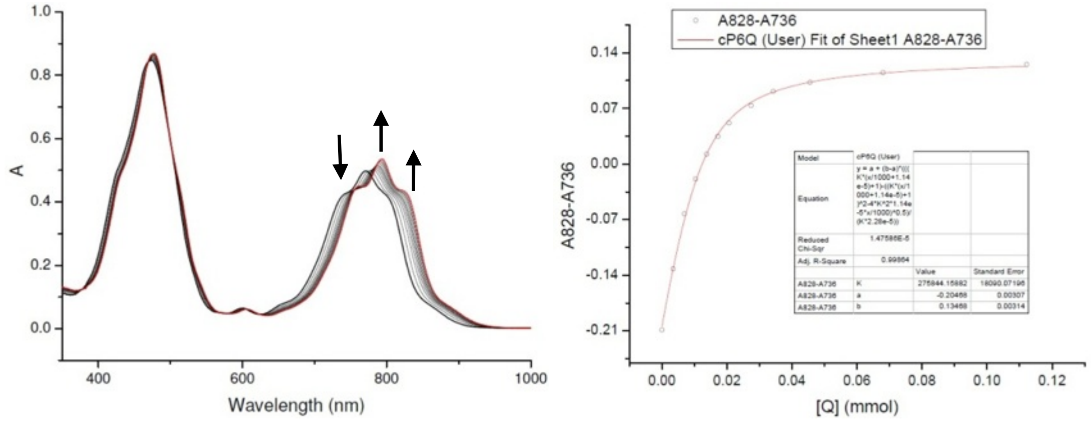

Fig. S42 UV-vis titration of quinuclidine and ***c*-P6**, R^2 = 0.998.
(Run 2, $CHCl_3$, 298 K, [***c*-P6**] = 1.9 μM).

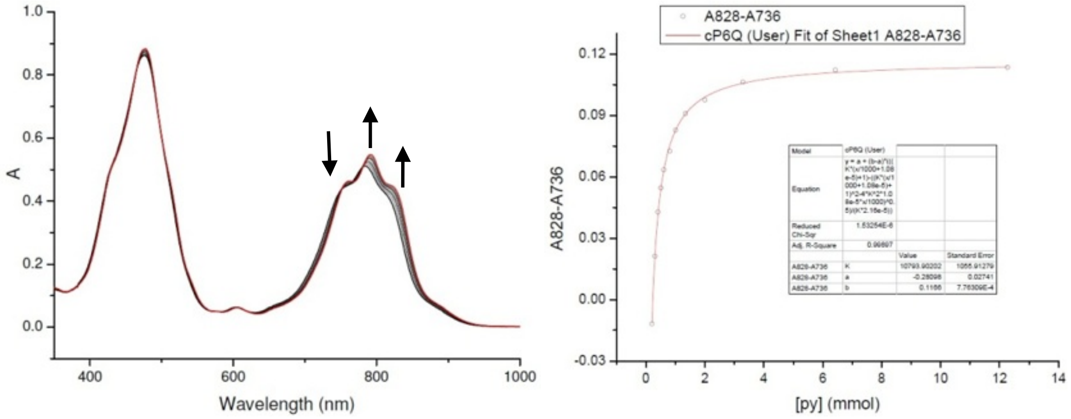

Fig. S43 UV-vis titration of pyridine and ***c*-P6**, R^2 = 0.998.
(Run 1, $CHCl_3$, 298 K, [***c*-P6**] = 1.8 μM).

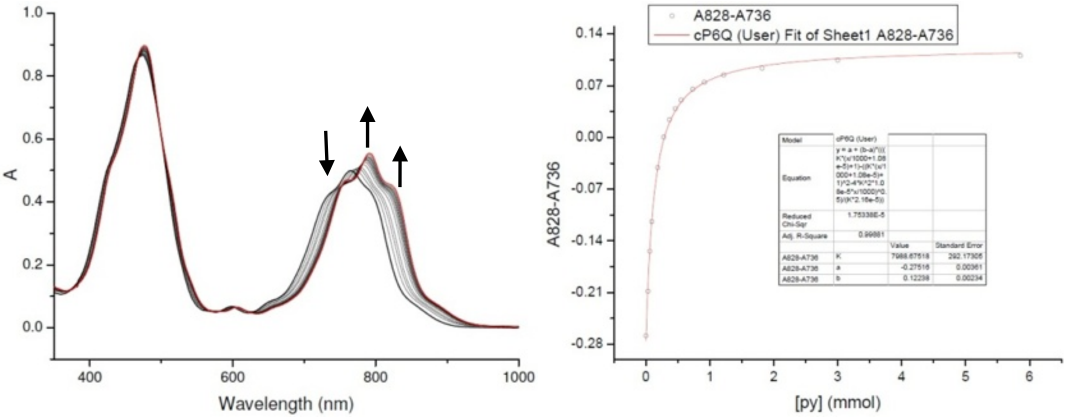

Fig. S44 UV-vis titration of pyridine and ***c*-P6**, R^2 = 0.998.
(Run 2, $CHCl_3$, 298 K, [***c*-P6**] = 1.8 μM).

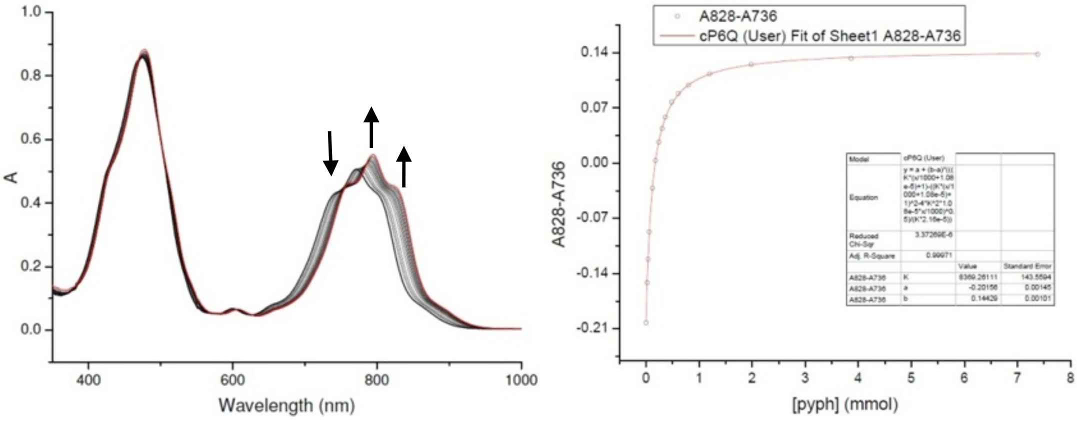

Fig. S45 UV-vis titration of 4-phenylpyridine and ***c*-P6**, R^2 = 0.999.
(Run 1, $CHCl_3$, 298 K, [***c*-P6**] = 1.8 μM).

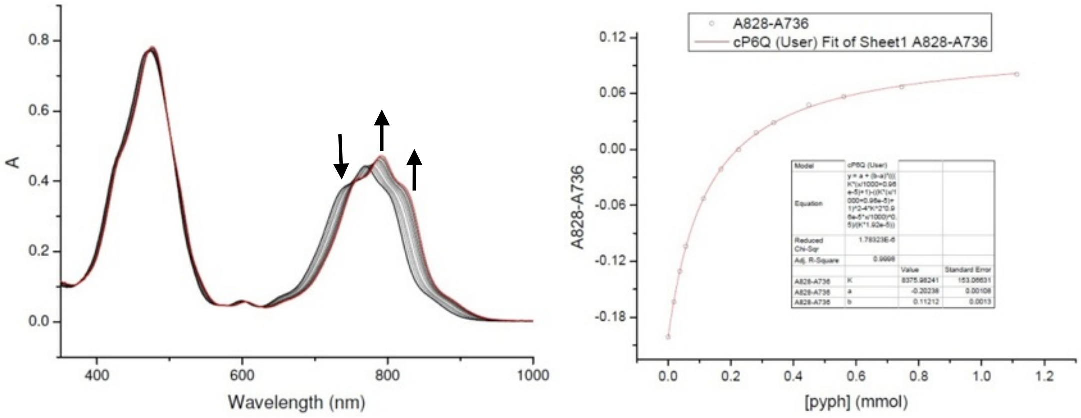

Fig. S46 UV-vis titration of 4-phenylpyridine and ***c*-P6**, R^2 = 0.999.
(Run 2, $CHCl_3$, 298 K, [***c*-P6**] = 1.6 μM).

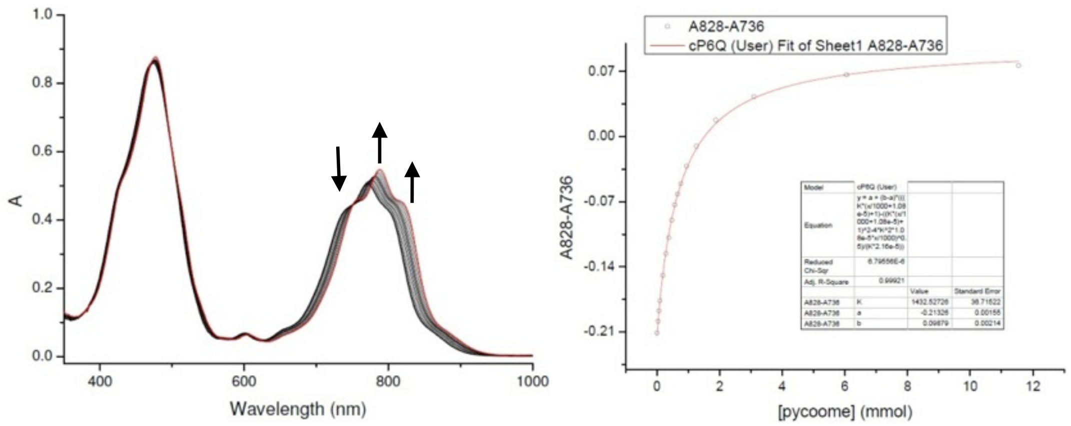

Fig. S47 UV-vis titration of methyl isonicotinate and ***c*-P6**, R^2 = 0.999.
(Run 1, $CHCl_3$, 298 K, [***c*-P6**] = 1.8 μM).

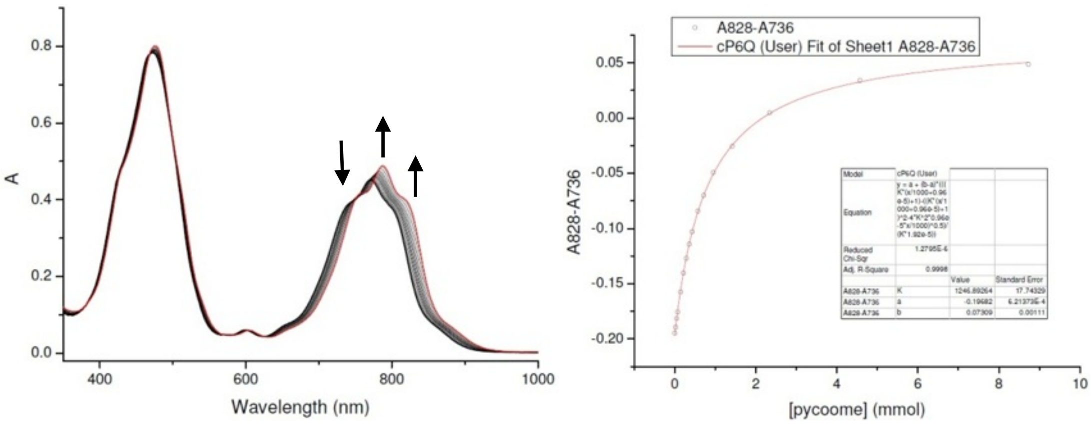

Fig. S48 UV-vis titration of methyl isonicotinate and ***c*-P6**, R^2 = 0.999.
(Run 2, $CHCl_3$, 298 K, [***c*-P6**] = 1.6 μM).

Table S3 The association constants of ***c*-P7** and ligands (1:1 association constants in M^{-1}).

Ligand	Run 1	Run 2	Average	Comments	Spectra
quinuclidine	$(1.2\pm0.2) \times 10^6$	$(1.0\pm0.4) \times 10^6$	$(1.1\pm0.3) \times 10^6$	[***c*-P7**] = 1.3 μM in both runs	**Fig. S49, S50**
pyridine	$(2.3\pm0.1) \times 10^4$	$(1.9\pm0.1) \times 10^4$	$(2.1\pm0.2) \times 10^4$	[***c*-P7**] = 0.5 μM in Run 1 and 1.3 μM in Run 2	**Fig. S51, S52**
**L3**	$(3.8\pm0.1) \times 10^4$	$(3.2\pm0.1) \times 10^4$	$(3.5\pm0.3) \times 10^4$	[***c*-P7**] = 1.1 μM in Run 1 and 1.4 μM in Run 2	**Fig. S53, S54**

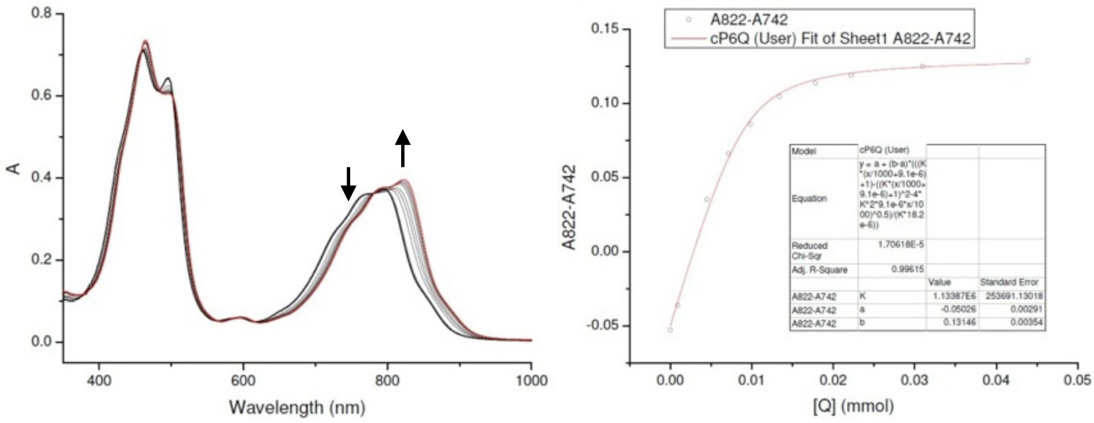

Fig. S49 UV-vis titration of quinuclidine and ***c*-P7**, R^2 = 0.996.
(Run 1, $CHCl_3$, 298 K, [***c*-P7**] = 1.3 μM).

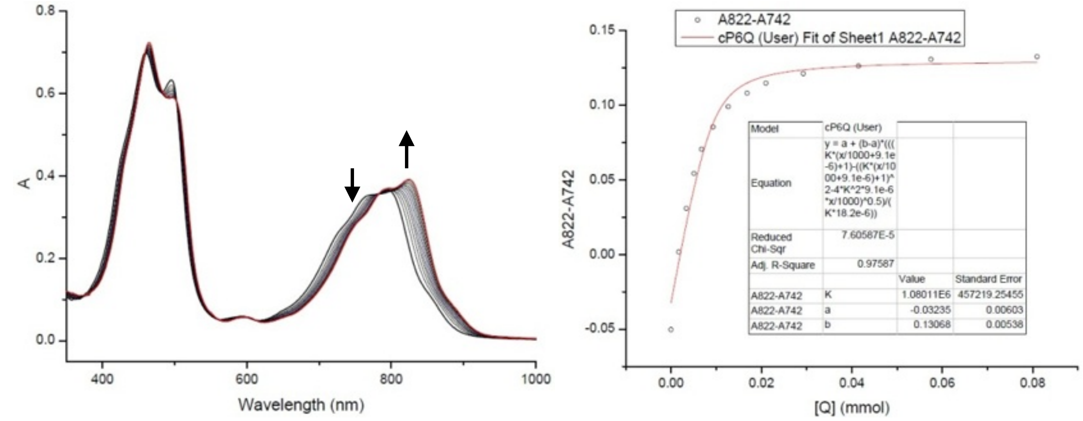

Fig. S50 UV-vis titration of quinuclidine and ***c*-P7**, R^2 = 0.975.
(Run 2, $CHCl_3$, 298 K, [***c*-P7**] = 1.3 μM).

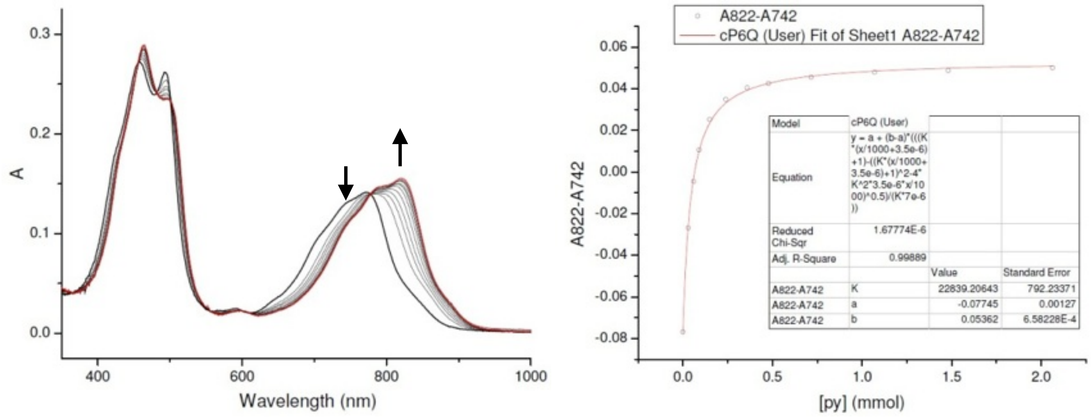

Fig. S51 UV-vis titration of pyridine and ***c*-P7**, R^2 = 0.998.
(Run 1, $CHCl_3$, 298 K, [***c*-P7**] = 0.5 μM).

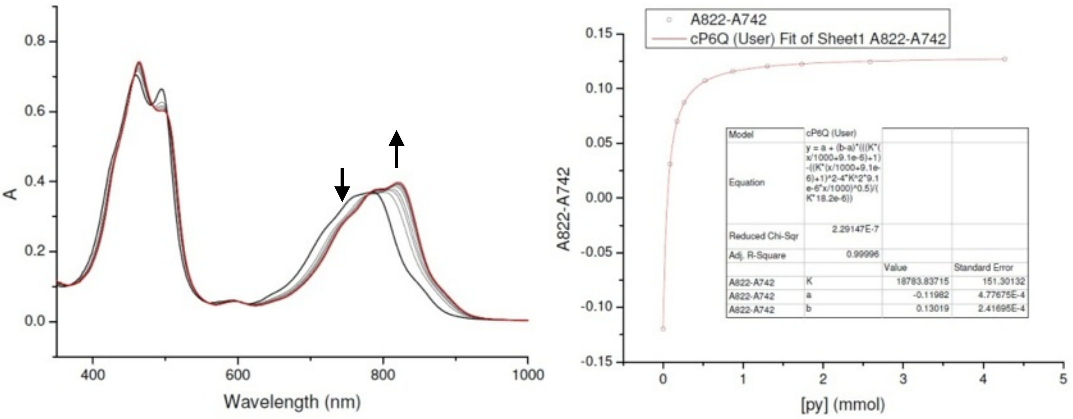

Fig. S52 UV-vis titration of pyridine and ***c*-P7**, $R^2 = 0.999$.
(Run 2, $CHCl_3$, 298 K, [***c*-P7**] = 1.3 μM).

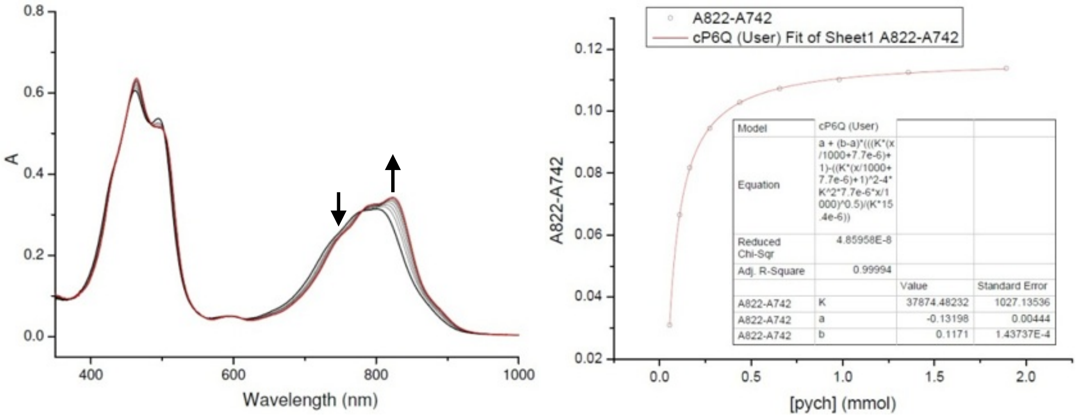

Fig. S53 UV-vis titration of 4-pyridinepropanoic acid methyl ester and ***c*-P7**, $R^2 = 0.999$.
(Run 1, $CHCl_3$, 298 K, [***c*-P7**] = 1.1 μM).

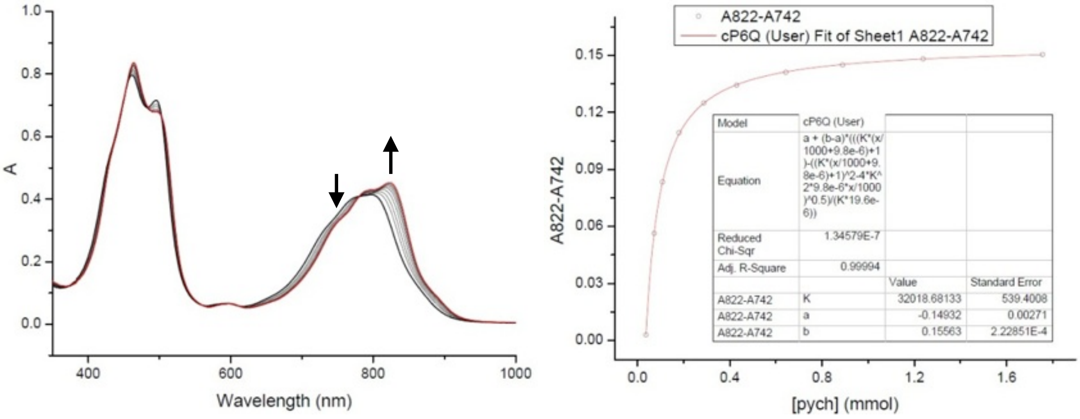

Fig. S54 UV-vis titration of 4-pyridinepropanoic acid methyl ester and ***c*-P7**, $R^2 = 0.999$.
(Run 2, $CHCl_3$, 298 K, [***c*-P7**] = 1.4 μM).

F3. Denaturation Titrations

c*-P6·T6** was titrated with quinuclidine only, because quinuclidine is the only commonly used ligand to displace the template from ***c*-P6·T6**. The corresponding denaturation constant is labeled as K_{qp6t6}. ***c*-P6·T6 and ***c*-P7·T7*** were titrated both with quinuclidine and pyridine. The corresponding denaturation constants are labeled as $K_{\mathrm{qp6t6*}}$, $K_{\mathrm{pyp6t6*}}$, $K_{\mathrm{qp7t7*}}$ and $K_{\mathrm{pyp7t7*}}$, respectively.

Unless stated otherwise, all the titrations were performed in chloroform (passed through short basic alumina column) at 298 K. All titrations were carried out at constant porphyrin concentration by adding porphyrin to the ligand stock solution before titrations started.

All the data were fitted to the *n*-dentate breaking-up binding isotherm, equation S.2.

$$\frac{A-A_{initial}}{A_{\infty}-A_{initial}}=\left(\frac{-K_{\mathrm{dn}}[\mathrm{L}]^{n}+\sqrt{K_{\mathrm{dn}}^{2}[\mathrm{L}]^{2n}+4K_{\mathrm{dn}}[\mathrm{L}]^{n}[\mathrm{P}]_{0}}}{2[\mathrm{P}]_{0}}\right) \quad (\mathrm{S.2})$$

where A is the observed absorption at a specific wavelength or difference of absorption in two wavelengths; $A_{initial}$ is the starting absorption at a specific wavelength or difference of absorption in two wavelengths; A_{∞} is the terminal absorption at a specific wavelength or difference of absorption in two wavelengths; K_{dn} is the dissociation constant between ligand and porphyrin nanoring complex; [L] is the concentration of ligand; $[\mathrm{P}]_0$ is the concentration of porphyrin nanoring complex; n is the number of binding sites of nanoring complexes. For ***c*-P6·T6** and ***c*-P6·T6***, n = 6; for ***c*-P7·T7***, n = 7.

The titration results are listed in the tables and figures below. In the spectra, the bold black lines represent starting points and the red lines represent terminal points.

Table S4. Denaturation constants of nanoring-template complexes with quinuclidine and pyridine.

Ligand, complex	Equilibrium constant	Run 1	Run 2	Average	Spectra
quinuclidine, ***c*-P6·T6**	K_{qp6t6} (M^{-5})	(1.0±0.1) × 10^{-3}	(8.4±0.4) × 10^{-4}	(9.2±0.8) × 10^{-4}	**Fig. S55, S56**
quinuclidine, ***c*-P6·T6***	$K_{\mathrm{qp6t6*}}$ (M^{-5})	(7.6±0.6) × 10^{3}	(8.3±0.7) × 10^{3}	(8.0±0.7) × 10^{3}	**Fig. S57, S58**
pyridine, ***c*-P6·T6***	$K_{\mathrm{pyp6t6*}}$ (M^{-5})	(3.9±0.4) × 10^{-6}	(4.7±0.4) × 10^{-6}	(4.3±0.4) × 10^{-6}	**Fig. S59, S60**
quinuclidine, ***c*-P7·T7***	$K_{\mathrm{qp7t7*}}$ (M^{-6})	(1.6±0.1) × 10^{10}	(2.3±0.2) × 10^{10}	(2.0±0.3) × 10^{10}	**Fig. S61, S62**
pyridine, ***c*-P7·T7***	$K_{\mathrm{pyp7t7*}}$ (M^{-6})	(1.9±0.1) × 10^{-2}	(2.4±0.1) × 10^{-2}	(2.2±0.3) × 10^{-2}	**Fig. S63, S64**

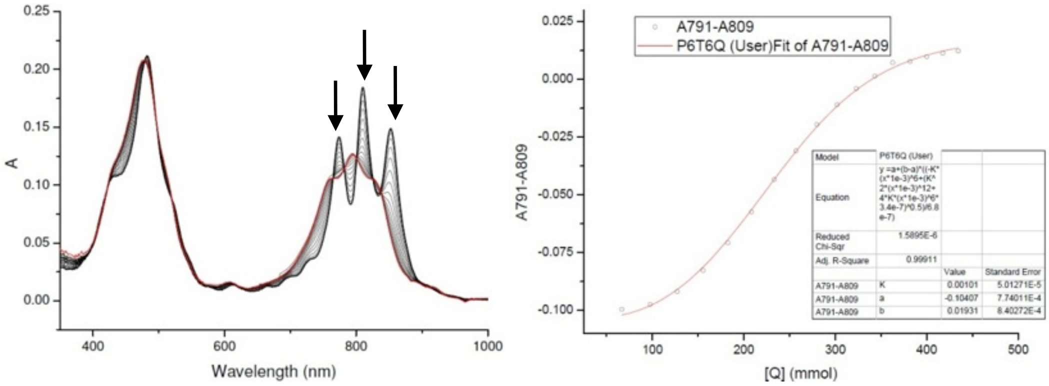

Fig. S55 UV-vis titration of quinuclidine and ***c*-P6·T6**, R^2 = 0.999.
(Run 1, $CHCl_3$, 297 K, [***c*-P6·T6**] = 0.34 μM).

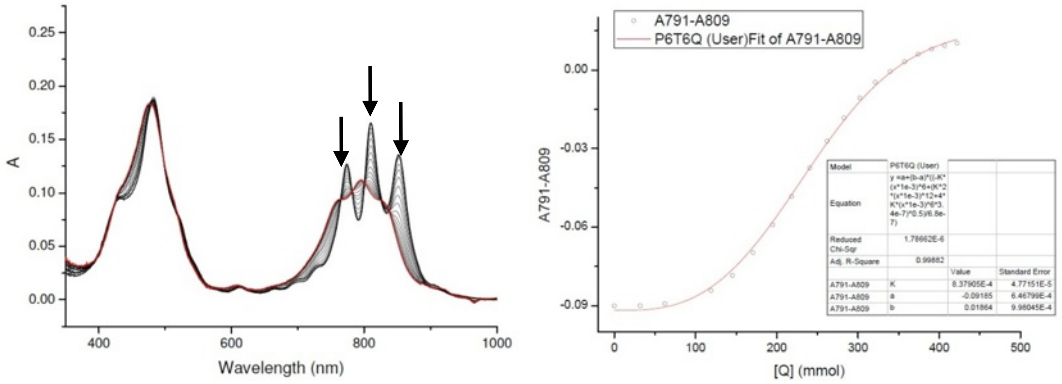

Fig. S56 UV-vis titration of quinuclidine and ***c*-P6·T6**, R^2 = 0.998.
(Run 2, $CHCl_3$, 298 K, [***c*-P6·T6**] = 0.34 μM).

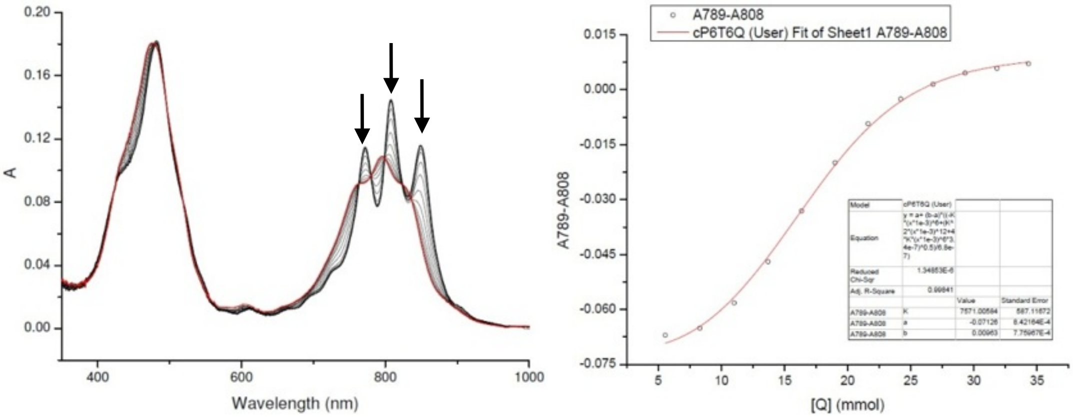

Fig. S57 UV-vis titration of quinuclidine and ***c*-P6·T6***, R^2 = 0.998.
(Run 1, $CHCl_3$, 298 K, [***c*-P6·T6***] = 0.34 μM).

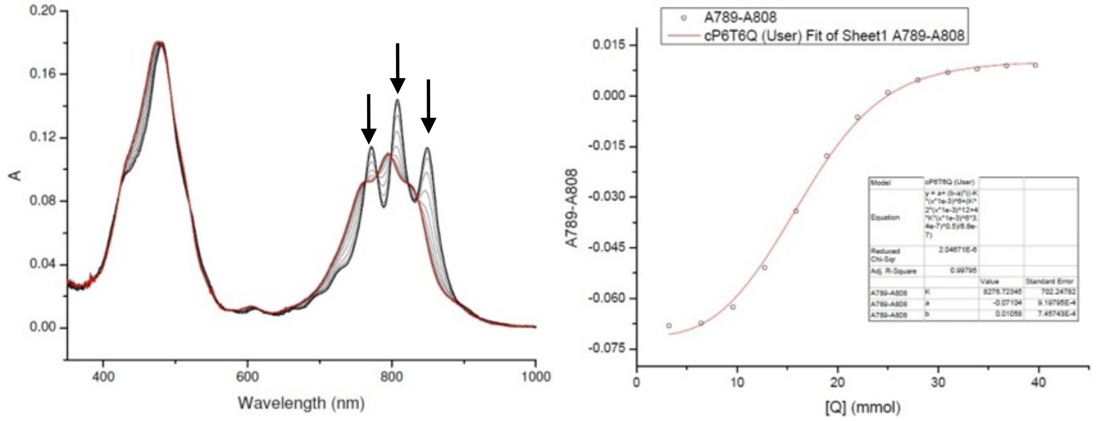

Fig. S58 UV-vis titration of quinuclidine and ***c*-P6·T6***, R^2 = 0.998.
(Run 2, $CHCl_3$, 298 K, [***c*-P6·T6***] = 0.34 μM).

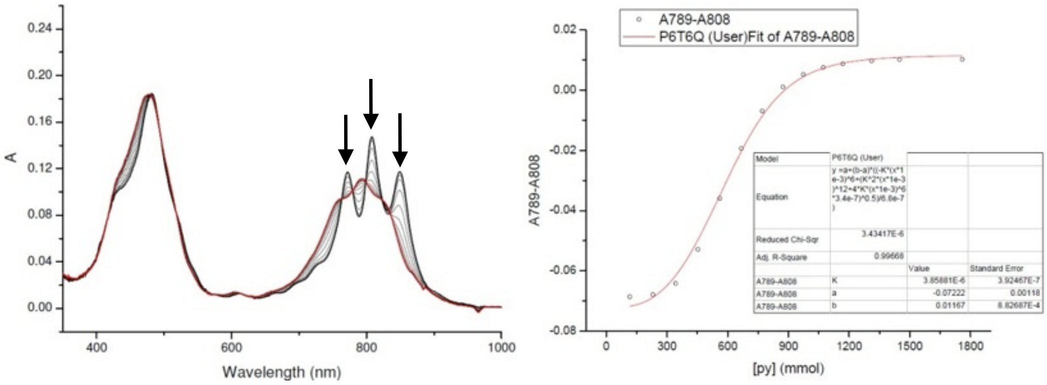

Fig. S59 UV-vis titration of pyridine and***c*-P6·T6***, R^2 = 0.996.
(Run 1, $CHCl_3$, 298 K, [***c*-P6·T6***] = 0.34 μM).

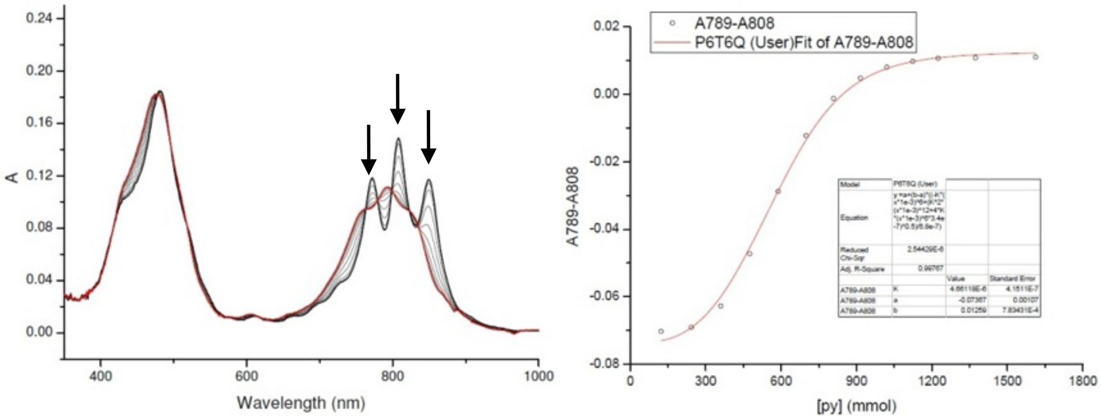

Fig. S60 UV-vis titration of pyridine and ***c*-P6·T6***, R^2 = 0.997.
(Run 2, $CHCl_3$, 298 K, [***c*-P6·T6***] = 0.34 μM).

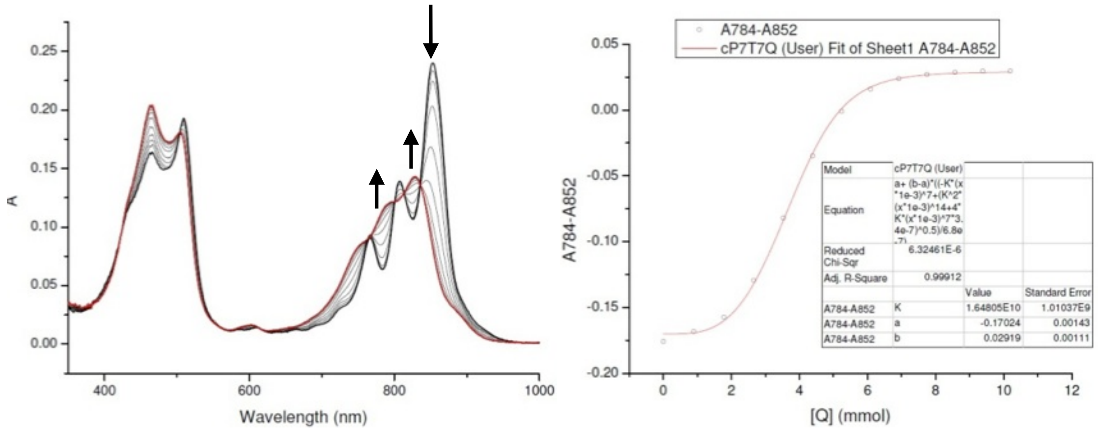

Fig. S61 UV-vis titration of quinuclidine and ***c*-P7·T7***, R^2 = 0.999.
(Run 1, $CHCl_3$, 298 K, [***c*-P7·T7***] = 0.34 μM).

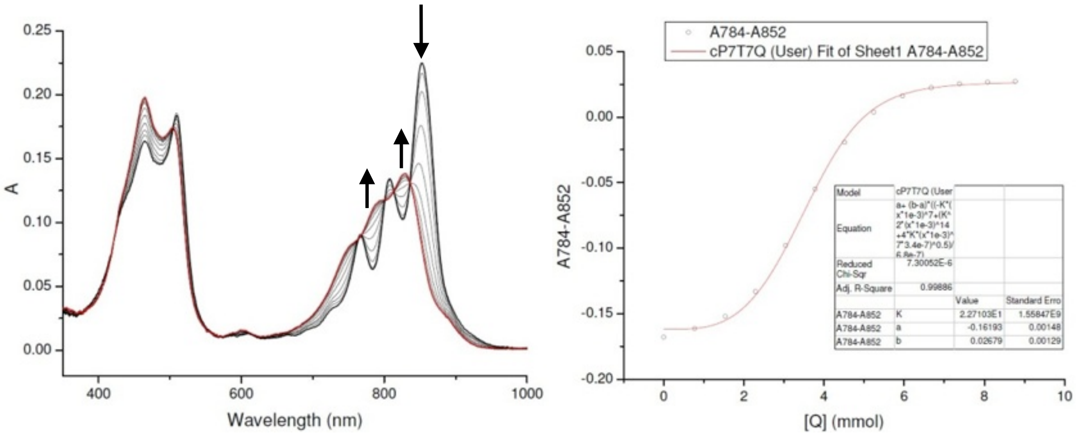

Fig. S62 UV-vis titration of quinuclidine and ***c*-P7·T7***, R^2 = 0.998.
(Run 2, $CHCl_3$, 298 K, [***c*-P7·T7***] = 0.34 μM).

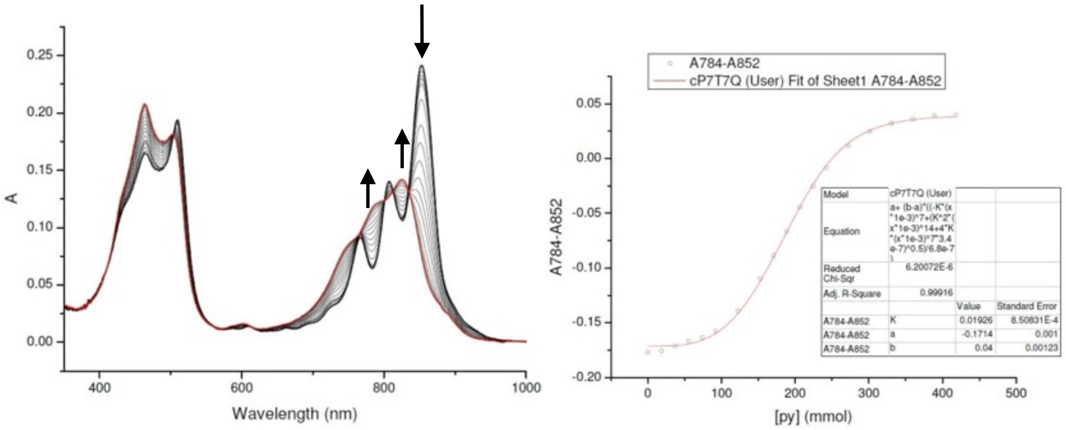

Fig. S63 UV-vis titration of pyridine and ***c*-P7·T7***, R^2 = 0.999.
(Run 1, $CHCl_3$, 298 K, [***c*-P7·T7***] = 0.34 μM).

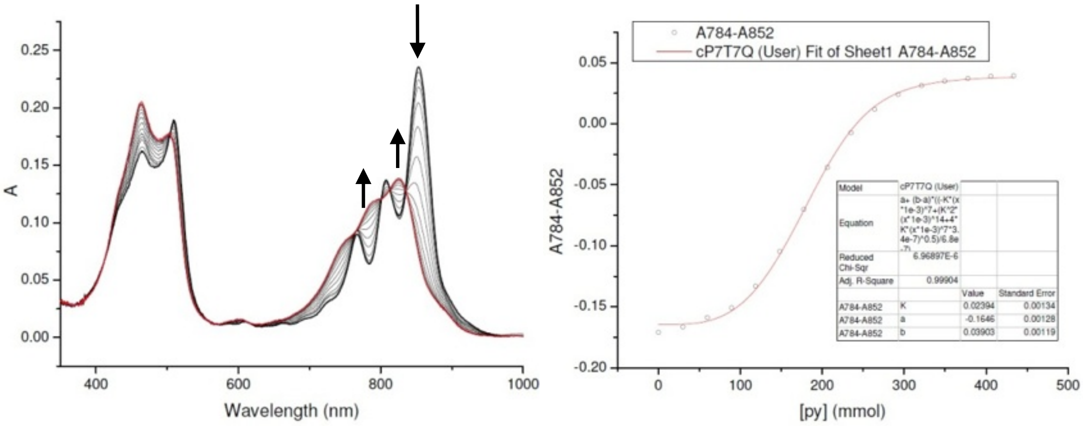

Fig. S64 UV-vis titration of pyridine and ***c*-P7·T7***, $R^2 = 0.999$.
(Run 2, $CHCl_3$, 298 K, [***c*-P7·T7***] = 0.34 μM).

F4. Calculation of Effective Molarities[S9]

The formation constants K_f of nanoring-template complexes were calculated from equation S.3,

$$\log K_{\mathrm{f}} = \log \frac{{K_{\mathrm{L}}}^{n}}{K_{\mathrm{dn,L}}} = n\log K_{\mathrm{L}} - \log K_{\mathrm{dn,L}} \tag{S.3}$$

where K_L is the association constants of the template-free ring ***c*-P*n*** with ligand L; $K_{dn,L}$ is the denaturation constants of the nanoring-template complex with this ligand; n is the chelation number of nanoring-template complexes. For ***c*-P6·T6** and ***c*-P6·T6***, $n = 6$; for ***c*-P7·T7***, $n = 7$. The uncertainty in LogK_f was calculated from equation S.4:

$$\Delta(\log K_{\mathrm{f}}) = \frac{1}{\ln 10}\sqrt{\left(\frac{n\Delta K_{\mathrm{L}}}{K_{\mathrm{L}}}\right)^2 + \left(\frac{\Delta K_{\mathrm{dn,L}}}{K_{\mathrm{dn,L}}}\right)^2} \tag{S.4}$$

The resulting values of log K_f are listed in Table S5:

Table S5 Formation constants of nanoring-template complexes.

Complex	log K_f (from quinuclidine denaturation)	log K_f (from pyridine denaturation)
***c*-P6·T6**	35.9 ± 0.2	—
c*-P6·T6	29.0 ± 0.2	29.2 ± 0.4
c*-P7·T7	32.0 ± 0.8	31.9 ±0.3

The geometric average effective molarities ($\overline{\mathrm{EM}}$) of the nanoring complexes were calculated from equation S.5,

$$\log\overline{\mathrm{EM}} = \log \sqrt[n-1]{\frac{K_{\mathrm{chem},n}}{K_1^n}} = \log \sqrt[n-1]{\frac{K_{\mathrm{f}}}{K_\sigma K_1^n}} = \frac{(\log K_{\mathrm{f}} - \log K_\sigma - n\log K_1)}{(n-1)} \tag{S.5}$$

where $K_{chem,n}$ is the statistically corrected value of K_f. As is shown in **Fig. S65**, for ***c*-P6·T6** and ***c*-P6·T6***, $K_\sigma = 768$; for ***c*-P7·T7***, $K_\sigma = 1792$. K_1 is the reference single-site microscopic binding constant statistically corrected for binding to one face of a porphyrin (i.e. half of the binding constants measured in reference association titrations in Section F2). The uncertainties in the values of log$\overline{\mathrm{EM}}$ were calculated from equation S.6:

$$\Delta(\log EM) = \frac{1}{(n-1)}\sqrt{\left(\Delta(\log \Delta K_{\mathrm{F}})\right)^2 + \left(\frac{n\Delta K_1}{\ln 10 K_1}\right)^2} \tag{S.6}$$

The geometric average effective molarities of the nanoring complexes are listed in Table S6:

Table S6 The geometric average effective molarities of nanoring-template complexes.

Complex	K_1 (M^{-1})	K_σ	$\log\overline{EM}$ (quinuclidine data)	$\log\overline{EM}$ (pyridine data)	$\overline{EM}$ (M)
***c*-P6·T6**	(4.2±0.2) × 10^3	768	2.25 ± 0.05	—	180 ± 20
c*-P6·T6	(6.5±0.5) × 10^2	768	1.85± 0.06	1.89± 0.09	74 ± 20
c*-P7·T7	(1.8±0.1) × 10^4	1792	–0.16±0.14	–0.17±0.07	0.7 ± 0.1

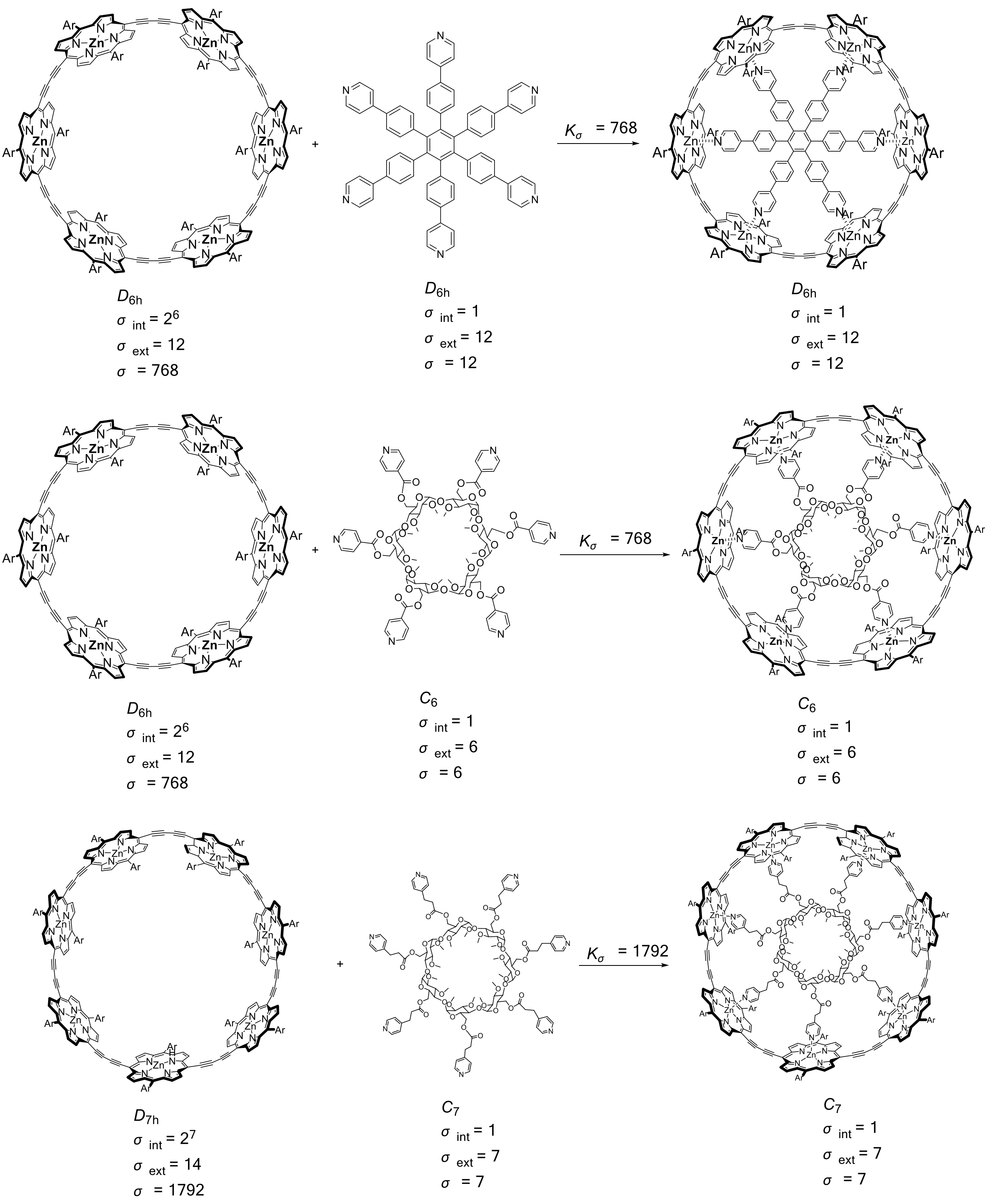

Fig. S65 Statistical factors of ***c*-P6·T6**, ***c*-P6·T6*** and ***c*-P7·T7***.

G. References

S1 P. N. Taylor, H. L. Anderson, *J. Am. Chem. Soc.* **1999**, *121*, 11538–11545.

S2 M. Hoffmann, J. Kärnbratt, M.-H. Chang, L. M. Herz, B. Albinsson, H. L. Anderson, *Angew. Chem. Int. Ed.* **2008**, *47*, 4993–4996.

S3 K. Takeo, K. Ueraura, H. Mitoh, *J. Carbohydr. Chem.* **1988**, *7*, 293–308.

S4 P. R. Ashton, E. Y. Hartwell, D. Philp, N. Spencer, J. F. Stoddart, *J. Chem. Soc., Perkin Trans. 2* **1995**, 1263–1277.

S5 L. A. Babadzhanova, N. V. Kirij, Y. L. Yagupolskii, W. Tyrra, D. Naumann, *Tetrahedron* **2005**, *61*, 1813–1819.

S6 M. C. O'Sullivan, J. K. Sprafke, D. Kondratuk, C. Rinfray, T. D. W. Claridge, A. Saywell, M. O. Blunt, J. N. O'Shea, P. H. Beton, M. Malfois, H. L. Anderson, *Nature* **2011**, *469*, 72–75.

S7 D. I. Svergun, C. Berbato, M. H. J. Koch, *J. Appl. Cryst.* **1995**, *28*, 768–773.

S8 D. I. Svergun, *J. Appl. Cryst.* **1992**, *25*, 495–503.

S9 H. J. Hogben, J. K. Sprafke, M. Hoffmann, M. Pawlicki, H. L. Anderson, *J. Am. Chem. Soc.* **2011**, *133*, 20962–20969.
